# Cholesterol-Based Compounds: Recent Advances in Synthesis and Applications

**DOI:** 10.3390/molecules24010116

**Published:** 2018-12-29

**Authors:** Hélio M. T. Albuquerque, Clementina M. M. Santos, Artur M. S. Silva

**Affiliations:** 1 QOPNA & LAQV-REQUIMTE, Department of Chemistry, University of Aveiro, Campus de Santiago, 3810-193 Aveiro, Portugal; clems@ipb.pt; 2 Centro de Investigação de Montanha (CIMO) Instituto Politécnico de Bragança, Campus de Santa Apolónia, 5300-253 Bragança, Portugal

**Keywords:** cholesterol, drug delivery, bioactive compounds, liquid crystals, gelators, bioimaging, synthesis

## Abstract

This review reports on the latest developments (since 2014) in the chemistry of cholesterol and its applications in different research fields. These applications range from drug delivery or bioimaging applications to cholesterol-based liquid crystals and gelators. A brief overview of the most recent synthetic procedures to obtain new cholesterol derivatives is also provided, as well as the latest anticancer, antimicrobial, and antioxidant new cholesterol-based derivatives. This review discusses not only the synthetic details of the preparation of new cholesterol derivatives or conjugates, but also gives a short summary concerning the specific application of such compounds.

## Index

1. Introduction to Cholesterol-Based Compounds12. Drug Delivery Applications……………………………………………………………………33. Anticancer, Antimicrobial, and Antioxidant Compounds…………………………………154. Cholesterol-Based Liquid Crystals……………………………………………………………275. Cholesterol-Based Gelators…………………………………………………………………….356. Bioimaging Applications……………………………………………………………………….417. Synthetic Applications………………………………………………………………………….488. Miscellaneous……………………………………………………………………………………569. Conclusions………………………………………………………………………………………59Funding………………………………………………………………………………………………..60Conflicts of Interest………………………………………………………………………………….60Abbreviations List…………………………………………………………………………………….60References………………………………………………………………………………………………62

## 1. Introduction to Cholesterol-Based Compounds

**Figure 1:**
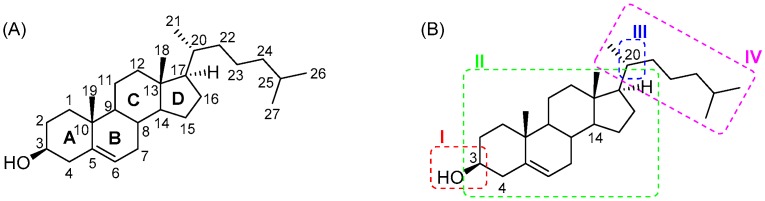
(**A**) Numbering and ring-labeling convention in cholesterol; (**B**) four domains of cholesterol.

Cholesterol (cholest-5-en-3β-ol) is considered to be a lipid-type molecule, being one of the most important structural components of cell membranes. Chemically, cholesterol is a rigid and almost planar molecule with a steroid skeleton of four fused rings, three six-membered and one five-membered, conventionally lettered from A to D (1,2-cyclopentanoperhydrophenanthrene ring system) (Figure 1A). Therefore, the cholesterol molecule comprises four essential domains (Figure 1B). In domain I, the polarity of the 3-hydroxy group constitutes an active site for hydrogen bond interactions with a myriad of biological molecules (e.g., phospholipids in membranes) [1]. In domain II, the absence of methyl groups at C-4 and C-14 influences directly the planarity of the molecule, while in domain III, the natural (*R*) configuration at C-20 determines the “right-handed” conformation of the side chain. Finally, in domain IV, the conformation and length of the side chain is of prime relevance to intermolecular contacts [2]. The presence of a hydrophilic 3-hydroxy headgroup on the A-ring, together with a hydrophobic hydrocarbon body, give the molecule an amphiphilic nature, which makes cholesterol the most recognized sterol.

Cholesterol plays a vital role in life, particularly in cell membranes and as a precursor to the biosynthesis of several steroid hormones. In cell membranes, which are essentially constituted by a double layer of phospholipids, cholesterol has great influence on membrane fluidity, microdomain structure (lipid rafts), and permeability by interacting with both the hydrophilic headgroups and the hydrophobic tails of phospholipids. In addition, modifications of the stereochemistry and oxidation states of the fused rings, the side chain, as well as the functional groups of cholesterol, lead to a wide variety of biologically important molecules, such as bile acids, vitamin D, and several steroid hormones [1,2]. Interestingly, 13 Nobel Prizes have been awarded to scientists who studied the structure of cholesterol, its biosynthetic pathway, and metabolic regulation. Unfortunately, cholesterol has gained a bad reputation because it is increasingly associated with several cardiovascular and neurodegenerative diseases, among others [1,3].

**Figure 2:**
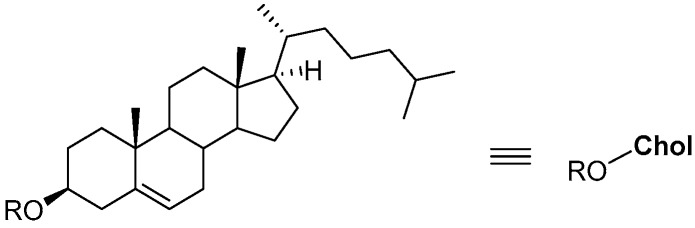
Schematic representation of cholesterol-based molecules throughout the manuscript.

Over the years, cholesterol has risen as an attractive starting material or a model system for organic synthesis due to its easily derivatized functional groups, availability, and low cost. Many useful chemical and enzymatic reactions are now widely used for multistep steroid transformations, leading to products of practical importance. The chemical transformations range from simple ones, such as manipulations of functional groups, to more complex ones, such as C­-H activation or C-C bond formation with organometallic reagents. In 2014, a purely synthetic chemistry review was published, dealing only with the advances in cholesterol chemistry since 2000, focusing on cholesterol oxidation reactions, substitution of the 3β-hydroxy group, addition to the C5=C6 double bond, C-H functionalization, and C-C bond forming reactions. However, this review paper excluded simple derivatization reactions of cholesterol such as the preparation of carboxylic and inorganic acid esters, aliphatic and aromatic ethers, simple acetals, or glycosides [4]. From our perspective, the simpler chemical transformations very often lead to the preparation of new cholesterol-based molecules with potential applications in several important research fields. Therefore, in this review, we focused our attention on publications from 2014 to date and described not only the synthesis of cholesterol-based new molecules, but also the application of these molecules in different fields, such as drug delivery; bioimaging; liquid crystals; gelators; anticancer, antimicrobial, and antioxidant applications; as well as purely synthetic applications. However, some interesting papers published before 2014 were included to fill some of the lacking papers from the 2014 review paper. Throughout the text, several reaction schemes will be depicted to describe the chemical reaction involved in the preparation of the cholesterol-based compounds. For simplification purposes, the structures of cholesterol will consistently be represented using the abbreviations depicted in Figure 2.

## 2. Drug Delivery Applications

Drug delivery is a method or process of administering a pharmaceutical compound to achieve a therapeutic effect in humans or animals. Drug delivery systems can in principle provide enhanced efficacy, reduced toxicity, or both for various types of drugs. Liposomes are the most common and well-investigated nanocarriers for targeted drug delivery because they have demonstrated efficiency in several biomedical applications by stabilizing therapeutic compounds, overcoming obstacles to cellular and tissue uptake, and improving the biodistribution of compounds to target sites in vivo [5]. 

**Scheme 1:**
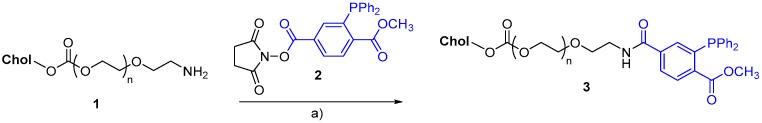
Synthesis of anchoring lipid Chol-PEG_2000_-triphenylphosphine. Reagents and conditions: **a**) Et_3_N, CH_2_Cl_2_, room temperature (rt), overnight.

In 2014, Vabbilisetty and Sun reported a study of terminal triphenylphosphine carrying anchor lipid effects on a liposome surface by postchemically selective functionalization via Staudinger ligation, using lactosyl azide as a model ligand. They synthesized two different anchor lipids, one of them based on the cholesterol molecule (Chol-PEG_2000_-thiphenylphosphine **3**), which was synthesized through an amidation reaction of synthetic Chol-PEG_2000_-NH_2_ **1** with 3-diphenylphosphino-4-methoxycarbonylbenzoic acid *N*-hydroxysuccinimide (NHS) active ester **2** (Scheme 1) [6].

The authors verified that the Staudinger ligation could be carried out under mild reaction conditions in aqueous buffers without a catalyst and in high yields. The encapsulation and releasing capacity of the glycosylated liposome based on cholesterol were evaluated, respectively, by entrapping 5,6-carboxyfluorescein (CF) dye and monitoring the fluorescence leakage. It was concluded that Chol-PEG_2000_-thiphenylphosphine **3** is particularly suitable for the ligation of water-soluble molecules and can accommodate many chemical functions, being potentially useful in the coupling of many other ligands onto liposomes for drug delivery purposes [6].

**Scheme 2:**
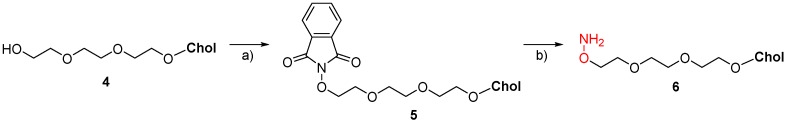
Synthesis of oxyamine-terminated cholesterol conjugate. Reagents and conditions: **a**) PPh_3_, *N*-hydroxy-phthalimide, diisopropyl azodicarboxylate (DIAD), tetrahydrofuran (THF), rt, 16 h; **b**) NH_2_NH_2_·H_2_O, CH_2_Cl_2_, rt, 18 h.

In 2015, a new method was reported for the deposition of a single lipid bilayer onto a hard polymer bead starting from discoidal bicelles and using chemoselective chemistry to hydrophobically anchor the lipid assemblies, using cholesterol bearing an oxyamine linker. The synthesis of oxyamine-terminated cholesterol **6** involved two steps, starting with a Mitsunobu reaction of compound **4**, followed by a reaction of **5** with hydrazine hydrate (Scheme 2) [7].

The discoidal bicelles were prepared in water media upon mixing dimyristoylphosphatidylcholine (DMPC), dihexanoylphosphatidylcholine (DHPC), dimyristoyltrimethylammonium propane (DMTAP), and the oxyamine-terminated cholesterol derivative **6**, in a specific molar ratio. These bicelles were exposed to aldehyde-bearing polystyrene (PS) beads and readily underwent a change to a stable single lipid bilayer coating at the bead surface. This approach may be advantageous in depositing membrane proteins at such surfaces for analytical, diagnostic, or therapeutic applications (namely drug delivery) [7].

**Scheme 3:**
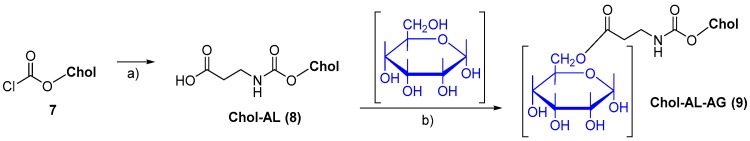
Synthesis of **Chol**-**AL**-**AG**. Reaction conditions: **a**) β-alanine, soluphor-P, NaOH, THF, rt, 2 h; **b**) arabinogalactan, carbonyldiimidazole (CDI), dimethylaminopyridine (DMAP), dimethyl sulfoxide (DMSO), 80 °C, 24 h.

Cholesterol chloroformate **7** was used as a lipid anchor for hydrophobization of arabinogalactan (AG), a liver-specific high galactose containing a branched polysaccharide, through a two-step reaction sequence that yielded a novel polysaccharide lipid, conjugated ligand **9** (**Chol**-**AL**-**AG**), with a bifunctional spacer β-alanine (AL) (Scheme 3) [8].

Ligand **9** was used to prepare conventional liposomes (CLs) and surface-modified liposomes (SMLs) through the reverse phase evaporation technique. These new liposomes were characterized by different techniques exhibiting the required particle size for targeting tumor and infectious cells. In vitro biological studies showed an enhanced binding affinity and cellular uptake of SMLs compared to CLs by HepG2 cells, making SMLs an interesting new approach for targeted drug delivery in liver cancer therapeutics [8].

**Scheme 4:**
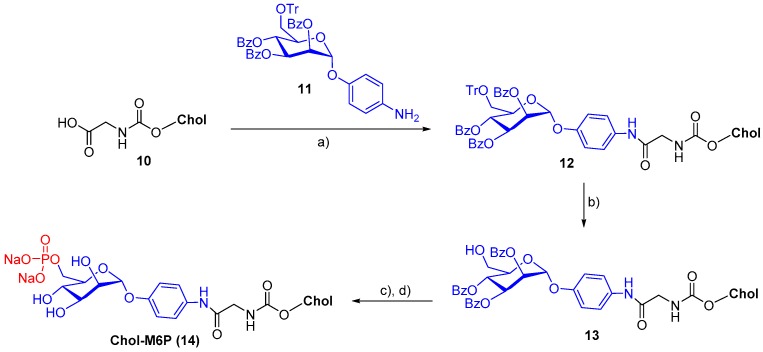
Synthesis of M6P cholesteryl conjugate (**Chol**-**M6P**). Reagents and conditions: **a**) *N*,*N*′-dicyclohexylcarbodiimide (DCC), DMAP, CH_2_Cl_2_, rt; **b**) *p*-TsOH, MeOH, CHCl_3_; **c**) POCl_3_, Et_3_N, dry CH_2_Cl_2_, 0 °C, rt; **d**) NaOMe, dry MeOH, then Dowex Na cation exchange resin.

Crucianelli et al. reported in 2014 a new delivery system based on liposomes containing dioleoylphosphatidilcholine (DOPC) and mannose 6-phosphate (M6P)-functionalized cholesterol **14**. For this purpose, M6P cholesteryl conjugate **14** (**Chol**-**M6P**) was synthesized following a three-step route starting from cholesterol derivative **10**, as depicted in Scheme 4 [9].

This novel vector system, designed to target lysosomes, was loaded with a model compound calcein to investigate intracellular trafficking in a 3T3-NIH cell line using a confocal and fluorescence microscopy technique. The affinity of the M6P group for the **CI-M6PR** receptor enabled these liposomes to carry calcein along the route leading to lysosomes, in opposition to calcein itself, which did not internalize into cells. These results suggest that liposomes containing **Chol-M6P 14** appear to be promising vectors in the selective targeting of lysosomes for enzyme replacement therapy or anticancer therapy [9].

**Scheme 5:**
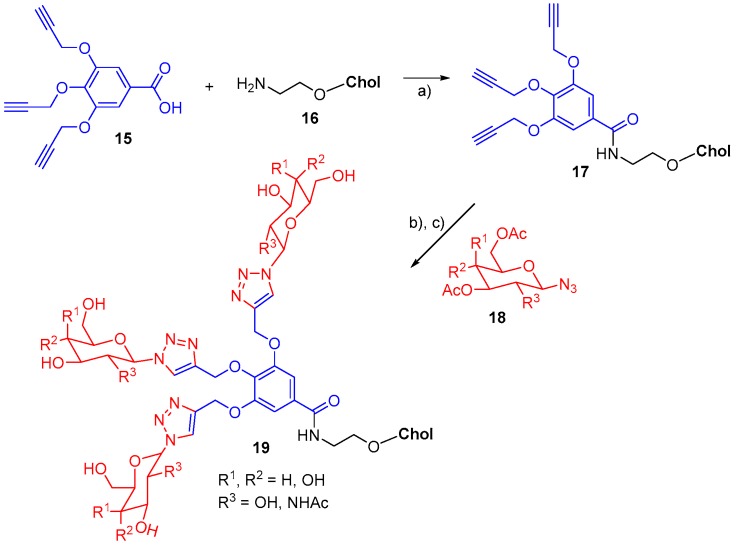
Synthesis of cholesterol-based neoglycoconjugates derived from d-galactose and *N*-acetylglucosamine. Reagents and conditions: **a**) 1-ethyl-3-(3-dimethylaminopropyl)carbodiimide hydrochloride (EDAC), DMAP, acetone/THF, rt, 4 h; **b**) glycosyl azide, CuSO_4_·5H_2_O, AscONa, THF/H_2_O, rt, 4 h; **c**) NaOMe, MeOH, 0 °C, 1 h.

The importance of liposomes in drug delivery applications is well recognized. In this context, Silva and coworkers reported the synthesis of cholesterol-based neoglycoconjugates of **19** (galactose-Gal and *N*-acetylglucosamine-GlcNAc) for further incorporation into liposomes. The glycoconjugates were synthesized through a copper-catalyzed 1,3-dipolar cycloaddition (CuAAC) reaction of glycosyl azides (**18**) with cholesterol derivative **17** (Scheme 5) [10]. The authors carried out biodistribution in vivo studies to evaluate the targeting of these carbohydrate-coated liposomes, concluding that they showed high uptake by the liver, spleen, and kidneys and no significant accumulation into other organs. Furthermore, it was demonstrated that liposomes with galactose in the surface preferentially target the liver cells. The results suggest that this kind of liposome might be a promising delivery system for therapeutic agents in hepatic diseases [10].

**Scheme 6:**
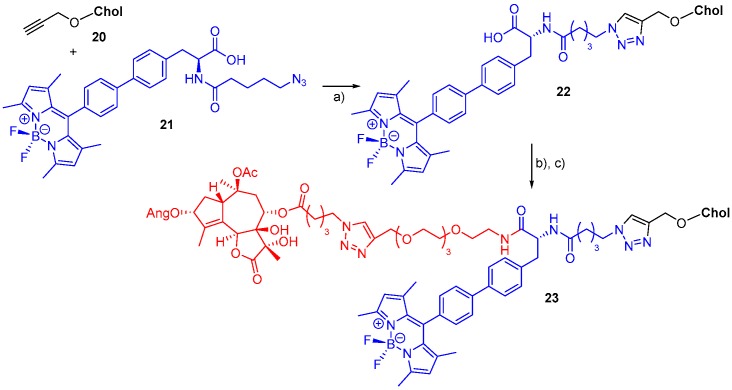
Synthesis of cholesterol-trilobolide conjugate. Reaction conditions: **a**) CuSO_4_·5H_2_O, AscONa, tris[(1-benzyl-1*H*-1,2,3-triazol-4-yl)methyl]amine (TBTA), dimethylformamide (DMF), microwave irradiation (MW), 60 °C, 90 min; **b**) amino-PEG_4_-acetylene, *N*-(3-dimethylaminopropyl)-*N*′-ethylcarbodiimide hydrochloride (EDCl), DMAP, *N*-hydroxybenzotriazole (HOBt), DMF, rt, 24 h; **c**) Tb-N_3_VA, CuSO_4_·5H_2_O, AscONa, TBTA, DMF, MW, 60 °C, 5 h.

To develop a drug delivery system for potential theranostic applications, Škorpilová et al. synthesized the fluorescent macrostructure **23** containing sesquiterpene lactone trilobolide (Tb), cholesterol, and a green-emitting boron dipyrromethene (BODIPY) dye. The synthesis of compound **23** involved a three-step sequence, starting from the CuAAC reaction of propargyl cholesterol **20** with BODIPY dye **21**, followed by functionalization with sesquiterpene lactone trilobolide (Scheme 6) [11]. This fluorescent cholesterol conjugate **23** was successfully incorporated into liposome formulations, which showed promising immunomodulatory properties in primary rat macrophages and improved drug distribution in U-2 OS and HeLa cancer cells. The study of the intracellular trafficking pattern of liposomes revealed two populations: One localized on the cell membrane and the other inside the cell, this last one closely related to cell death. This new liposomal cholesterol conjugate **23** not only retains the biological properties of pure trilobolide, but also enhances bioavailability, and thus has potential for use in theranostic applications [11].

**Scheme 7:**
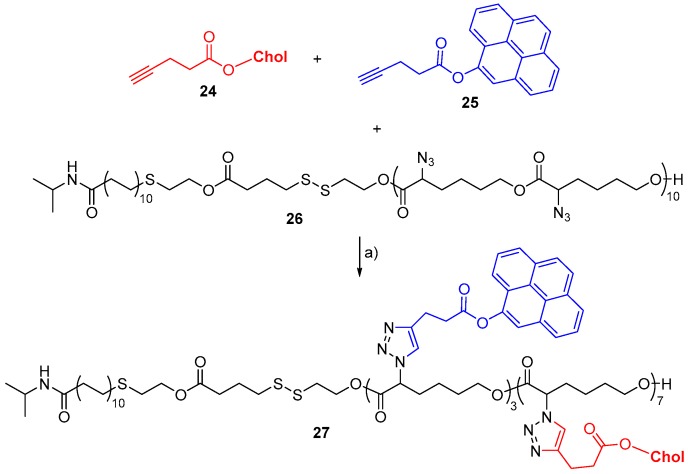
Synthesis of fluorescent triple-responsive block-graft copolymer. Reagents and conditions: **a**) CuI, Et_3_N, THF, 40 °C, 24 h.

Recently, Lin et al. reported the synthesis of a fluorescent triple-responsive block-graft copolymer **27**, bearing cholesteryl- and pyrenyl-side groups, with a disulfide (S-S) bridging point joining the hydrophilic and hydrophobic chains. The synthesis of such polymers relied on a typical click reaction between PNiPAAm_10_-S-S-P(αN_3_CL)_10_ **26**, pyrenylmethyl 4-pentynoate **25**, and cholesteryl 4-pentynoate **24**, affording PNiPAAm_10_-S-S-P(αN_3_CL_10_-g-PyrePA_3_/-CholPA_7_) **27** (Scheme 7) [12]. Experimental results indicated that copolymer **27** could undergo self-assembly into polymeric micelles with excellent fluorescence performance in aqueous solution. The drug-loading capacity of cholesteryl-grafted copolymer **27** was evaluated using doxorubicin (DOX) as a template drug, and the results showed reasonable DOX-loading capacity. The authors also demonstrated that DOX-loaded micelles enter the cells at a substantially faster rate than their free-form counterparts, effectively inhibiting HeLa cell proliferation [12].

**Scheme 8:**
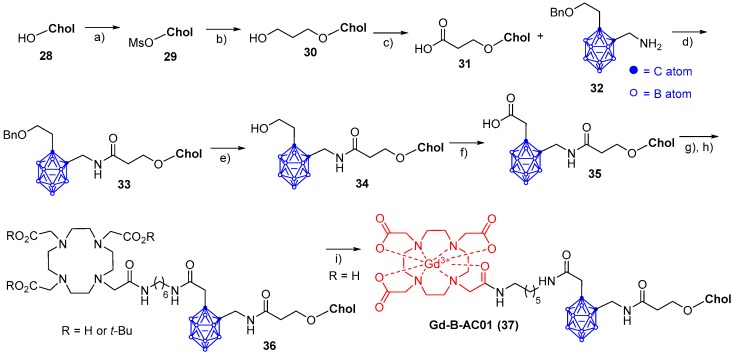
Synthesis of **Gd-B-AC01**. Reagents and conditions: **a**) MsCl, Et_3_N, Et_2_O, 0 °C to rt, 1 h; **b**) HO(CH_2_)_3_OH, 1,4-dioxane, 120 °C, overnight; **c**) 3 M CrO_3_ in H_2_SO_4_, acetone, rt, overnight; **d**) DCC, CH_2_Cl_2_, rt, overnight; **e**) H_2_, Pd/C, EtOH/CH_2_Cl_2_ (1:1), rt, overnight; **f**) 3 M CrO_3_ in H_2_SO_4_, acetone, rt, overnight; **g**) *N*-tert-butylDOTAMA-C6-NH_2_, *N*-hydroxysuccinimide (NHS), DCC, i-PrEt_2_N, CH_2_Cl_2_, rt, overnight; **h**) trifluoroacetic acid (TFA), CH_2_Cl_2_, rt, 4 h; **i**) GdCl_3_, 1 M NaOH, H_2_O, rt, 26 h.

In 2014, the synthesis of a new dual-imaging and therapeutic agent for improved efficacy in Boron Neutron Capture Therapy (BNCT) in cancer treatment was reported [13]. The compound consists of a carborane unit (ten boron atoms) bearing a cholesterol unit on one side (to pursue incorporation into the liposome bilayer) and a Gd(III)/1,4,7,10-tetraazacyclododecane monoamide complex on the other side (as an magnetic resonance imaging (MRI) reporter to attain the quantification of the B/Gd concentration). The synthesis of the target compound Gd-B-AC01 (**37**) relied on an eight-step synthetic strategy, which ended with the complexation of **36** with Gd(III) in aqueous solution at pH 6.5 (Scheme 8). This dual probe **37** was functionalized with a polyethylene glycol (PEG)ylated phospholipid containing a folic acid residue at the end of the PEG chain. These liposomes presented interesting features such as the ability to selectively concentrate high amounts of boron in human ovarian cancer cells (IGROV-1), enough to perform efficient BNCT treatment with significantly reduced uptake by healthy cells in the surrounding regions. Furthermore, these liposomes, which can be used as nanoplatforms to deliver both Gd and B agents, can, in principle, be used for the simultaneous delivery of antitumor drugs such as DOX [13].

**Figure 3:**
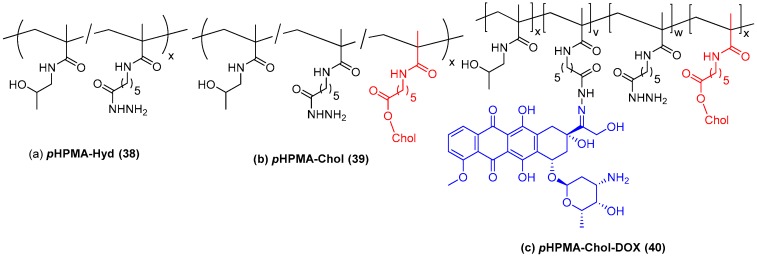
Schematic structures of **a**) cholesterol-free *p*HPMA-based copolymers (***p*HPMA-Hyd**) bearing hydrazide groups; **b**) statistical cholesterol-containing (***p*HPMA-Chol**) groups distributed along the polymer chain; and **c**) cholesterol-containing *p*HPMA-based copolymers carrying the anticancer drug doxorubicin (DOX) (***p*HPMA-Chol-DOX**).

Zhang and coworkers studied the behavior of nanoparticles (NPs) formed by self-assembly of amphiphilic poly[*N*-(2-hydroxypropyl)methacrylamide] (*p*HPMA) copolymers bearing cholesterol side groups (**39**) as potential drug carriers for solid tumor treatment (Figure 3) [14].

The behavior of such NPs in human serum albumin (HSA) protein environment was evaluated using mixed solutions of NPs from polymer conjugates with or without the anticancer drug doxorubicin bounded to them, **39** and **40**, respectively. The authors found that in the absence of DOX, a small amount of HSA molecules bind to the cholesterol groups of the NPs by diffusing through the loose *p*HPMA shell or get caught in meshes formed by the ***p*HPMA** chains. On the other hand, the presence of DOX strongly hinders these interactions, and for that reason the delivery of DOX by these NPs in the human body is not affected by the presence of HSA [14].

**Scheme 9:**
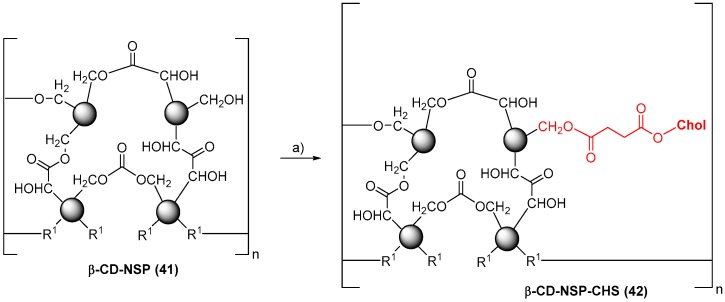
Synthesis of **β-CD-NSP-CHS**. Reagents and conditions: **a**) cholesterol hydrogen succinate (CHS), EDCl, DMAP, DMF, 45 °C, 24 h.

Recently, Singh and coworkers reported the biofunctionalization of the surface of β-cyclodextrin nanosponge **41** (**β-CD-NSP**) with cholesterol, expecting to improve its cellular binding ability. The β-CD-NSP was functionalized by grafting cholesterol hydrogen succinate (CHS) through a coupling reaction, affording **β-CD-NSP-CHS 42** (Scheme 9) [15].

The cytotoxicity assays showed that **β-CD-NSP 41** was nontoxic and that the surface biofunctionalized with CHS **42** improved both the therapeutic and drug delivery efficacy of DOX. The experimental results also demonstrated that CHS grafting may enhance DOX adsorption due to the hydrophobic charge on the surface. Therefore, the surface-engineered CD-NSP could be used as a carrier for low water-soluble small drug molecules to improve solubility and bioavailability in site-specific drug delivery systems [15].

**Scheme 10:**
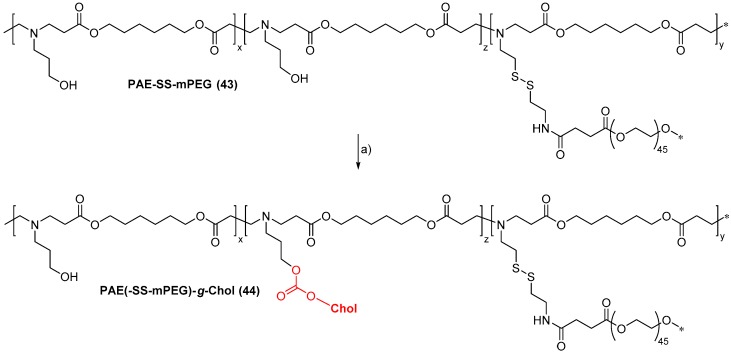
Synthesis of **PAE(-ss-mPEG)-*g*-Chol**. Reagents and conditions: **a**) cholesteryl chloroformate, DMAP, CH_2_Cl_2_, rt, 24 h. * represents only the momomeric structure of the polymer.

In attempting to develop an intelligent drug delivery for cancer chemotherapy, Li et al. synthesized dual redox/pH-sensitive amphiphilic copolymer **44** and cholesterol-modified poly(β-amino esters)-grafted disulfide poly (ethylene glycol) methyl ether [**PAE(-SS-mPEG)-*g*-Chol**]. The precursor PAE-SS-mPEG **43** was successfully synthesized via Michael-type step polymerization using disulfide linkage-containing PEG segment. Finally, cholesterol was incorporated into the hydroxy-pendant group trough an esterification reaction, affording the copolymer **PAE(-SS-mPEG)-*g*-Chol 44** (Scheme 10) [16].

The authors verified the interesting physicochemical properties of copolymer **44**, namely redox and pH sensitivity. Doxorubicin-loaded hybrid polymer-lipid NPs (DOX-HDPLNPs) were prepared, and drug-loading capacity, delivery efficacy, and redox- and pH-triggered drug release behavior in vitro were studied. The results showed that DOX-HDPLNPs enhanced loading capacity and improved cellular uptake ability, as well as serum stability. The anticancer potential in tumor-bearing mice was addressed, indicating that the DOX-HDPLNPs prepared with redox- and pH-sensitive copolymer with disulfides and PEGylated lipid could efficiently enhance therapeutic efficacy with low cytotoxicity and side effects. Both in vitro and in vivo experiments indicated that DOX-HDPLNPs enhanced therapeutic efficacy with high cellular uptake and negligible cytotoxicity compared to the free drug DOX. Therefore, HDPLNPs can be considered to be smart delivery systems for hydrophobic anticancer drug delivery [16].

**Figure 4:**
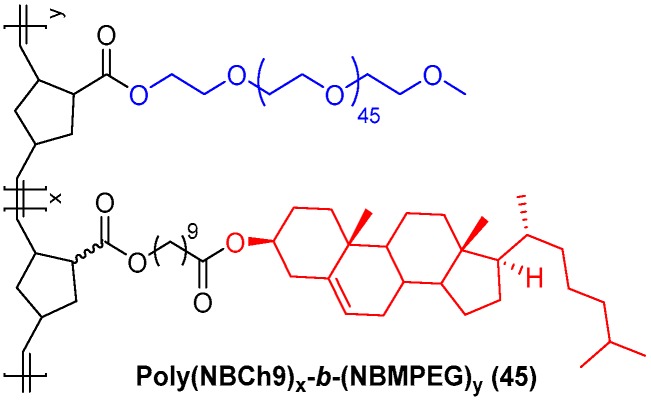
Cholesterol-based brush block copolymer **poly(NBCh9)_x_-*b*-(NBMPEG)_y_**.

Tran et al. developed a copolymer in 2014, constituted of polynorbonene-cholesterol/ poly(ethylene glycol) [**P(NBCh9-*b*-NBPEG**)] **45**, that undergoes self-assembly to form a long circulating nanostructure capable of encapsulating the anticancer drug DOX with high drug loading (Figure 4) [17].

The authors found that the doxorubicin-loaded nanoparticles (DOX-NPs) were effectively internalized by human cervical cancer cells (HeLa) and that they showed dose-dependent cytotoxicity. Moreover, the DOX-NPs showed good in vivo circulation time and preferential accumulation in tumor tissue with reduced accumulation in the heart and other vital organs, and significantly inhibited tumor growth in tumor-bearing severe combined immunodeficient (SCID) mice. Based on these results, DOX-NPs can become useful carriers in improving tumor delivery of hydrophobic anticancer drugs [17].

**Scheme 11:**
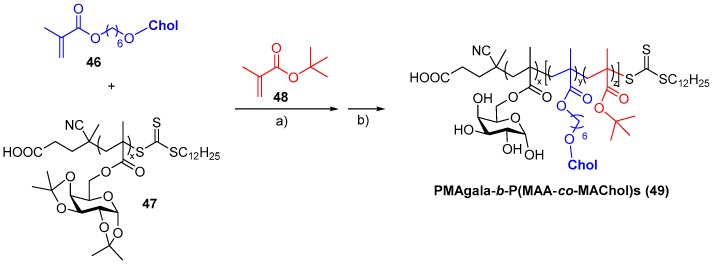
Synthesis of **PMAgala-*b*-P(MAA-*co*-MAChol)s**. Reaction conditions: **a**) 2,2′-azobis(2-methylpropionitrile) (AIBN), toluene, 80 °C, 8 h; **b)** TFA/CH_2_Cl_2_ (1:2), rt, 32 h.

A new series of amphiphilic diblock terpolymer poly(6-*O*-methacryloyl-d-galactopyranose)- -*b*-poly(methacrylic acid-*co*-6-cholesteryloxyhexyl methacrylate) bearing attached galactose and cholesterol grafts [**PMAgala-*b*-P(MAA-*co*-MAChol)s**] **49** were prepared via Reversible Addition Fragmentation chain Transfer (RAFT) copolymerization followed by deprotection of galactose in the presence of trifluoroacetic acid (TFA) (Scheme 11) [18].

The new terpolymers (**49**) were studied for in vitro DOX release, and the results revealed high stability of the DOX-loaded terpolymer micelles under neutral conditions and significantly fast responsive DOX release. In addition, the results of fluorescence microscopy revealed that the DOX encapsulated in the synthesized diblock terpolymer PMAgala_18_-*b*-P(MAA_26_-*co*-MAChol_9_)/DOX micelles could be uptaken and delivered into cell nuclei in an efficient way, and their intracellular trafficking pathway could be altered compared to the free DOX control. The new terpolymers (**49**) could therefore be strongly considered for future smart nanoplatforms toward efficient antitumor drug delivery [18].

**Scheme 12:**
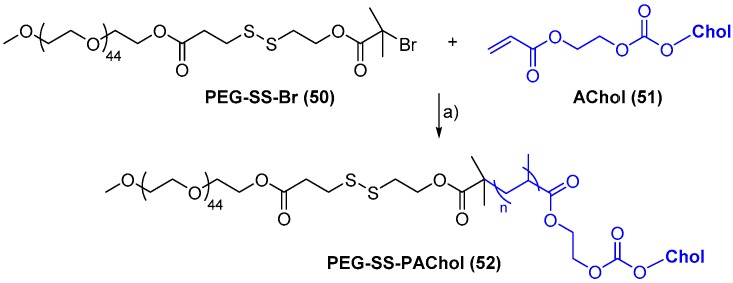
Synthesis of reduction-sensitive block copolymer **PEG-SS-PAChol**. Reagents and conditions: **a**) CuBr, *N*,*N*,*N*′,*N*″,*N*″-pentamethyldiethylenetriamine (PMDETA), toluene, 80 °C, 18 h.

In 2014, a reduction-responsive polymersome based on the amphiphilic block copolymer **PEG-SS-PAChol 52** was developed. The synthesis of **52** was achieved using **PEG-SS-Br 50**, a versatile atom transfer radical polymerization (ATRP) macroinitiator, and a cholesterol-containing acrylate **51**, using CuBr as a catalyst and *N*,*N*,*N*′,*N*″,*N*″-pentamethyldiethylenetriamine (PMDETA) as a ligand (Scheme 12) [19].

The polymersome **52** was studied to come up with robust nanocarriers able to release their content inside the cells upon contact with the intracellular reducing environment. The physical crosslinking by a smectic phase of **52** in the hydrophobic sublayer, as well as the introduction of a disulfide bridge that links the hydrophilic PEG and hydrophobic blocks present in **52**, were key features that gave stability, robustness, and reduction sensitivity to the polymersome. The results showed sensitivity of the block copolymer **52** to reduction, and the fluorescence dequenching of calcein both in glutathione (GSH) solution and in vitro with the mouse macrophage cells pretreated with GSH-OEt demonstrated the breakdown of polymersome under reduction conditions. To achieve significant calcein release, high concentrations of GSH and long incubation times were necessary. These reduction-responsive polymersomes (**52**) could be used as drug carriers with very long circulation profiles and slow release kinetics [19].

**Scheme 13:**
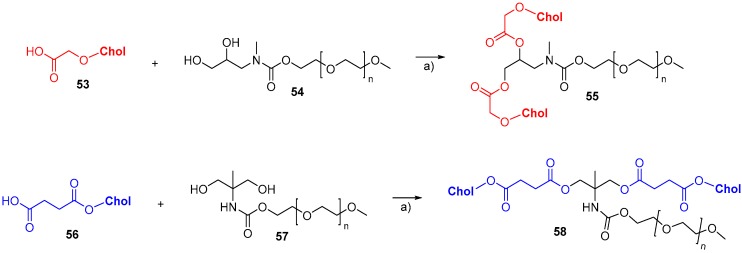
Synthesis of sterol-anchored polyethylene glycols (PEGs). Reagents and conditions: **a**) EDCl, DMAP, CHCl_3_, rt, 24 h.

Recently, two new sterol-anchored polyethylene glycols, **55** and **58**, were reported as potential alternatives to conventional phosphatidylethanolamine-PEGs. Their synthesis relied on the esterification reaction of cholesterol derivatives **53** and **56** with PEGs **54** and **57**, as depicted in Scheme 13 [20].

The authors studied the biophysical properties of liposomes containing these two sterol-anchored PEGs, **55** and **58**, which exhibited an array of canonical PEGgylated-liposome behaviors including retention of encapsulated small molecules, low serum protein adsorption, and reduced cellular uptake, yet they did not exhibit long circulation [20].

**Scheme 14:**
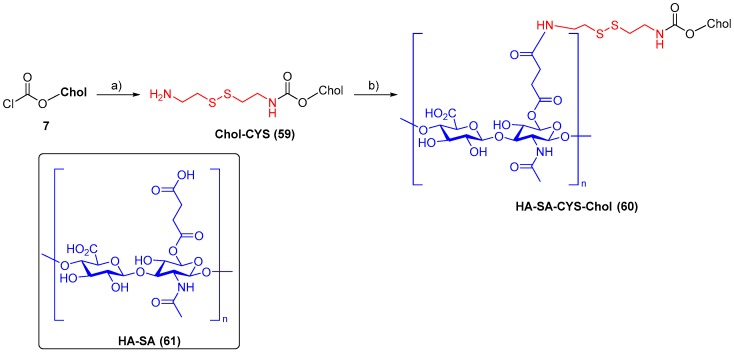
Synthesis of **HA-SA-CYS-Chol**. Reagents and conditions: **a**) Cystamine dihydrochloride, NaOH, CHCl_3_, 50 °C, 2 h; **b**) HA-SA, PyBOP, DMAP, DMSO/FM (4:1), rt, 2 days.

Polymeric micelles are known for their variety of therapeutic applications. In this field, two amphiphilic polymers were successfully synthesized using hyaluronic acid (HA), cholesterol, and octadecanoic acid as hydrophobic groups. Only the synthesis of cholesterol containing polymer **HA-SA-CYS-Chol 60** is depicted in Scheme 14, since the other hydrophobic groups do not fit in the scope of this paper. Nevertheless, the authors concluded that different properties of hydrophobic groups of the amphiphilic carrier are closely implicated in the stability and drug-loading capacity of the amphiphilic carrier and micelles. **HA-SA-CYS-Chol 60** presented a lower critical micellar concentration, producing docetaxel (DTX)-loaded micelles of a smaller particle size, higher encapsulation efficiency, and drug loading, when compared to the other hydrophobic tails [21]. Furthermore, in vivo animal studies revealed very good tumor-targeting properties and efficient antitumor effects at very low concentrations, with low systemic toxicity of **HA–SA–CYS–Chol 60** micelles [21].

**Scheme 15:**
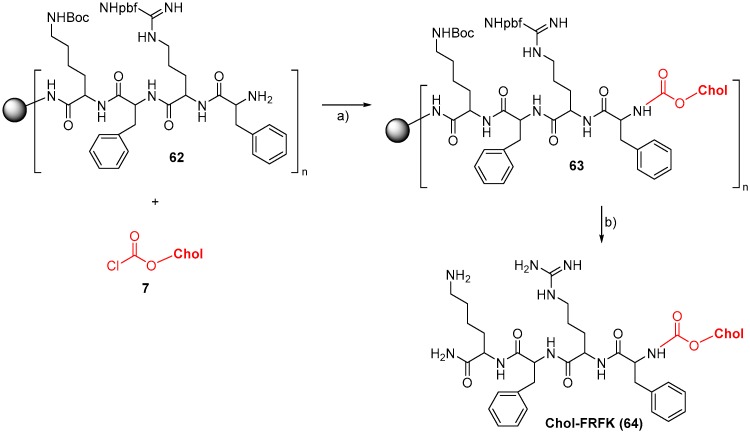
Synthesis of cholesterol-phenylalanine-arginine-phenylalanine-lysine (**Chol-FRFK**). Reagents and conditions: **a**) *N*,*N*-diisopropylethylamine (DIPEA), CH_2_Cl_2_, rt, 16 h; **b**) TFA/TIS/H_2_O (95:2.5:2.5), 28 °C, 16 h.

A new liposomal formulation for drug delivery purposes was recently developed, based on the *N*-terminal cholesterol conjugation with a mitochondria-penetrating peptide (MPP) sequence, consisting of four amino acids [phenylalanine-arginine-phenylalanine-lysine (FRFK)]. More specifically, the synthesis of cholesterol-phenylalanine-arginine-phenylalanine-lysine (**Chol-FRFK**) **64** was achieved by coupling cholesteryl chloroformate **7** with amino acid-bound resins (**62**), followed by resin cleavage using TFA and the removal of protecting groups (Scheme 15) [22]. The authors developed the liposomes using dioleoyl-*sn*-glycero-3-phosphoethanolamine (DOPE) and **Chol-FRFK 64** for delivery of the hydrophobic drug antimycin A specifically targeted toward mitochondria and lung cancer A549 cells. The results indicated that this formulation can effectively deliver the encapsulated drug to the mitochondria because of the small size and moderately cationic charge of the liposomes, enabling cellular uptake with low toxicity. The liposomes were found to be stable for long periods at room temperature, and they acted synergistically with antimycin A, leading to the complete disruption of inner membrane potential [22].

**Scheme 16:**
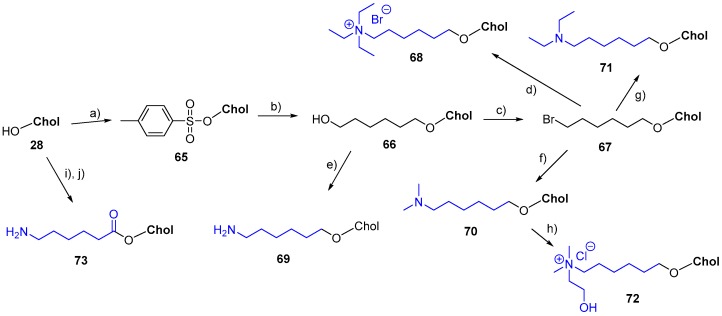
Synthesis of cholesterol-based cationic lipids. Reagents and conditions: **a**) *p*-TsCl, pyridine, CHCl_3_, rt, 23 h; **b**) hexane-1,6-diol, anhydrous 1,4-dioxane, reflux, 7 h; **c**) CBr_4_, PPh_3_, CH_2_Cl_2_, rt, 6 h; **d**) Et_3_N, MeOH/EtOAc (1:1), 90 °C in screw-top pressure tube, 48 h; **e**) HN_3_, PPh_3_, DIAD, 3 days; **f**) Me_2_N·HCl, NaOH, toluene, 90 °C in screw-top pressure tube, 72 h; **g**) Et_2_N, K_2_CO_3_, MeOH/EtOAc (1:1), 90 °C in screw-top pressure tube, 48 h; **h**) 2-chloroethanol, acetonitrile, 90 °C in screw-top pressure tube, 24 h; **i**) Boc-aminohexanoic acid, DCC/NHS, THF, 0 °C, 7 h; **j**) TFA, CH_2_Cl_2_, 0 °C, 4 h.

In 2016, six new cholesterol-derived cationic lipids, **68**–**73**, were synthesized via ether or ester linkages with different head groups (Scheme 16), which were used to create cationic liposomes for nonviral gene delivery vectors [23]. The authors studied the relationship between the structure of the synthesized lipids and the transfection efficiency and optimized gene transfection conditions of the liposomes. They found that the chemical structure of head groups and the linkage between cholesterol and head groups play important roles in gene delivery efficiency. Furthermore, lipids **69** and **73** exhibited higher transfection efficiency and lower toxicity than those of the tested commercial liposomes DC-Chol and lipofectamine 2000, even in the presence of serum [23].

**Scheme 17:**
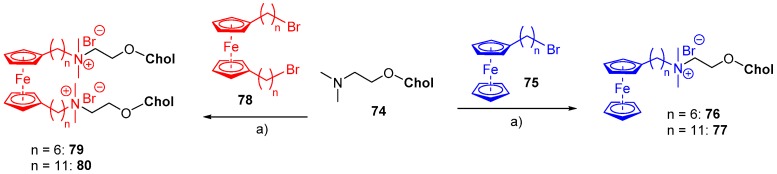
Synthesis of redox-active ferrocene containing cationic monomeric (CHM-C6F and CHM-C11F) and gemini (CHD-C6F and CHD-C11F) cholesteryl lipids. Reagents and conditions: **a**) MeOH/EtOAc (1:1), reflux, 4–6 days.

In 2015, Vulugundam and coworkers reported the design and synthesis of new redox-active monomeric **76** and **77**, and dimeric (gemini) **79** and **80**, cationic lipids based on ferrocenylated cholesterol derivatives for the development of gene delivery systems (Scheme 17). The cationic cholesterols **76** and **77**, as well as **79** and **80**, were incorporated into co-liposomes and shown to be transfection-efficient. The authors found that redox activities of co-liposomes and their lipoplexes could be regulated using the alkyl ferrocene moiety. The vesicles possessing ferrocene in the reduced state induced an efficient gene transfection capability using pEGFP-C3 plasmid DNA in three cell lines, even better than the commercial lipofectamine 2000 (Lipo 2000). This evidence suggests that these redox-driven systems could be used in gene delivery applications where transfection needs to be performed spatially or temporally [24].

**Scheme 18:**
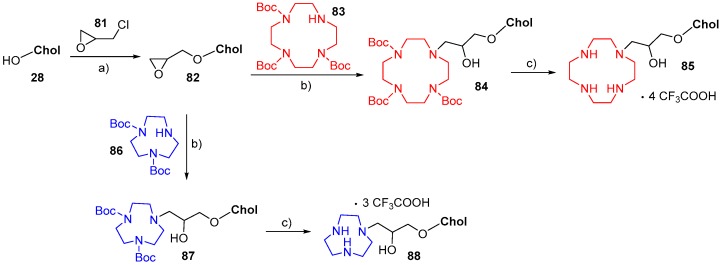
Synthesis of macrocyclic polyamine (cyclen and 1,4,7-triazacyclononane (TACN))-based cationic lipids bearing a cholesterol tail. Reagents and conditions: **a**) tetrabutylammonium bromide (TBAB), NaOH, H_2_O, 40 °C, 24 h; **b**) EtOH, reflux, 60 h; **c**) TFA, CH_2_Cl_2_, rt, 6 h.

A series of macrocycle polyamine (cyclen and 1,4,7-triazacyclononane (TACN))-based cationic lipids **85** and **88**, bearing cholesterol as a hydrophobic tail, were synthesized through ring-opening reactions (Scheme 18). These cationic lipids, **85** and **88**, were used in combination with 1,2-dioleoyl-*sn*-glycero- -3-phosphoethanolamine (DOPE) to prepare lipoplexes, which efficiently condense DNA into nanoparticles with a proper size and zeta potential [25].

Lipid **85**, containing cyclen as a headgroup, demonstrated lower toxicity and better transfection efficiency (TE) in vitro, when compared to the commercial reference lipofectamine 2000 in both 7402 and A549 cancer cells. Furthermore, the authors rationalized the good serum tolerance of **85** due to the presence of a hydroxy group in its structure. These promising results indicated that cationic-lipid **85** should be considered for nonviral gene vectors in in vivo applications [25].

**Scheme 19:**
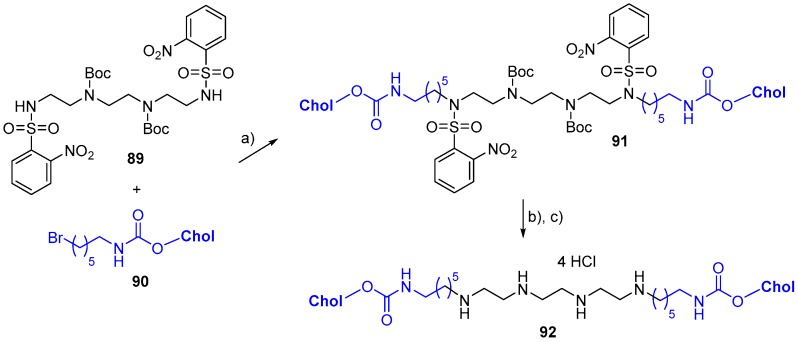
Synthesis of polycationic amphiphile based on triethylenetetramine. Reagents and conditions: **a**) Cs_2_CO_3_, DMF, 80 °C, 5 h; **b**) C_6_H_5_SH, K_2_CO_3_, DMF, rt, 1.5 h; **c**) 4 M HCl, 1,4-dioxane/CH_2_Cl_2_ (1:5), rt, 1.5 h.

Aiming to extend the existent library of polycationic amphiphiles, Puchkov et al. designed and synthesized a new molecule, **92**, based on triethylenetetramine and cholesterol (a spermine analogue containing the same number of amino groups but differing in the number of methylene units). The synthesis of the polycationic amphiphile **92** was based on the selective transformation of primary amines into secondary ones via nitrobenzenesulfonamides, and the molecule of cholesterol was incorporated through alkylation of bis(sulfonamide) **89** with bromo derivative of cholesterol **90** (Scheme 19) [26]. The authors used the triethylenetetramine-based amphiphile **92** to prepare cationic liposomes and concluded that the transfection properties of delivery nucleic acids in eukaryotic cells were inferior to those with amphiphiles based on spermine. Despite the polyamines (triethylenetetramine and spermine) having the same number of amino groups, their distribution was significantly different, which may have resulted in the difference in their transfection activity [26].

**Scheme 20:**
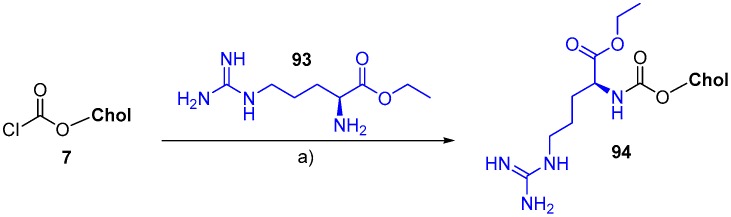
Synthesis of cholesterol-arginine conjugate. Reagents and conditions: **a**) THF, 10–15 °C, 6–7 h.

A newly designed arginine-conjugated cholesterol derivative, **94**, was recently reported for the preparation of cationic liposomes and their interaction with paclitaxel (PTX), a widely used anticancer drug. The synthesis of cholesterol-arginine ester (CAE) conjugate **94** was carried out through *N*-amidation of l-arginine ethyl ester **93** with cholesteryl chloroformate **7** (Scheme 20) [27].

The authors conducted molecular dynamic simulations as well as in vitro studies with the PTX-loaded liposomes. The results showed that these cationic liposomes enhanced loading efficiency and stability over the conventional liposomes, which can be rationalized based on the hydrogen bonding between CAE and PTX and the deeper penetration of PTX in the bilayer. Moreover, these novel liposomes demonstrated improved cytotoxicity in three different cell lines (MDA MB 231, H5V, and HDMEC) and enhanced endothelial cell migration inhibition compared to conventional liposomes. The absence of genotoxicity makes cholesterol-arginine ester **94** an interesting biocompatible cationic ligand in drug delivery applications [27].

**Scheme 21:**
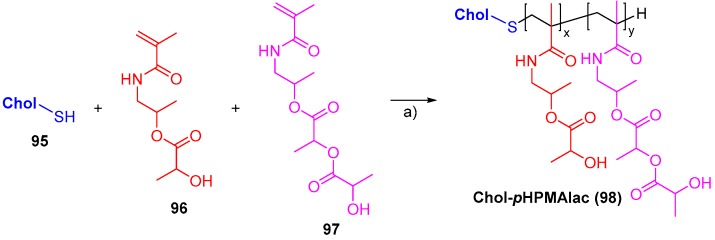
Synthesis of poly(*N*-(2-hydroxypropyl)methacrylamide mono/dilactate with a cholesterol anchor (**Chol-*p*HPMAlac**). Reagents and conditions: **a**) AIBN, 1,4-dioxane, 70 °C, 24 h.

The design and synthesis of thermosensitive polymers of *N*-(2-hydroxypropyl)methacrylamide mono/dilactate of different molecular weights and composition bearing a cholesterol anchor **98** (**Chol-*p*HPMAlac**) was reported in 2014 (Scheme 21). These new cholesterol-based polymers **98** were incorporated onto liposome formulations loaded with DOX. The authors concluded that the release of DOX from such liposome formulations was effective at low temperatures and could be adjusted according to the grafting density of **Chol-*p*HPMAlac 98**. **Chol-*p*HPMAlac 98** with a cloud point of 19.0 °C and a *M*_n_ of 10.0 kDa showed interesting releasing features because it was stable at body temperature, releasing its content only under hyperthermia conditions. These releasing features make these liposomes interesting for local drug delivery using hyperthermia [28].

**Scheme 22:**
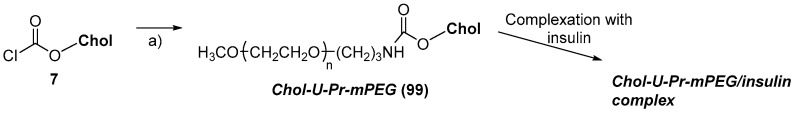
Synthesis of **Chol-U-Pr-mPEG** and complexation with insulin. Reagents and conditions: **a**) aminopropyl mPEG, Et_3_N, CHCl_3_, 40 °C, 24 h.

Recently, Asayama and coworkers reported a byproduct-free PEGylation method for the modification of insulin. The strategy involves the reaction of cholesterol chloroformate **7** with aminopropyl mPEG in the presence of triethylamine to afford the conjugate **Chol-U-Pr-mPEG 99** (Scheme 22), complexation with insulin in aqueous solution, and subsequent freeze-drying [29].

The **Chol-U-Pr-mPEG**/insulin complex not only preserved the insulin conformation, but also was shown to be effective in its protection from hydrolysis by protease and in the suppression of blood glucose levels in mice [29].

## 3. Anticancer, Antimicrobial, and Antioxidant Compounds

**Scheme 23:**
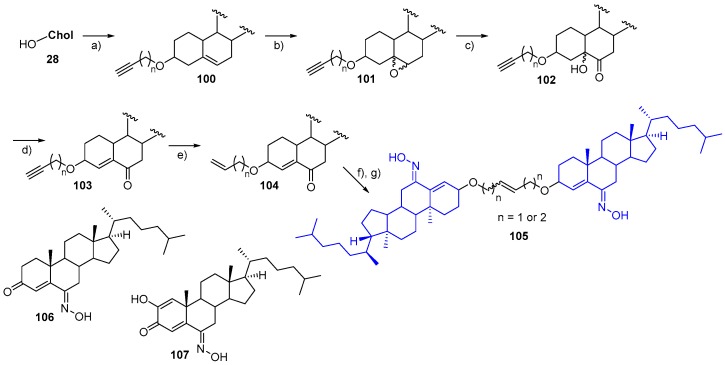
Synthesis of (6*E*)-hydroximinosteroid homodimers. Reagents and conditions: **a**) montmorillonite K-10, CHCl_3_, 55 °C, 7 days; **b**) *m*-chloroperoxybenzoic acid (*m*-CPBA), CHCl_3_, rt, 20 h; **c**) CrO_3_, H_2_O, butan-2-one, rt, 2 h; **d**) SO_2_Cl_2_, pyridine, 0 °C, 45 min; **e**) H_2_, Lindlar catalyst, pyridine, rt, 5 h; **f**) Grubbs 1st generation catalyst, CH_2_Cl_2_, 45 °C, overnight; **g**) NH_2_OH·HCl, pyridine, rt, 1 h.

Many new cholesterol derivatives bearing a wide range of bioactive scaffolds have been developed in the search for new anticancer, antimicrobial, or antioxidant agents with improved efficacy. In this context, Rodríguez et al. described an efficient synthesis of (6*E*)-hydroximinosteroid homodimers (**105**) linking two steroidal monomers at position 3 of the steroid scaffold via ruthenium-catalyzed cross-metathesis reaction (Scheme 23). The synthesis of the precursor monomers was carried out through a five-step reaction sequence starting from cholesterol **28** (Scheme 23) [30].

The cytotoxic activity of (6*E*)-hydroximinosteroid homodimers (**105**) was evaluated in vitro using human lung carcinoma A549, colon adenocarcinoma HCT-116, human Caucasian glioblastoma multiform T98G, and human pancreatic adenocarcinoma PSN1 cells. Only homodimer **105** (*n* = 2) showed selective cytotoxicity against HCT-116 cells: However, it presented no activity against the remaining cell lines. Nevertheless, the monomer counterparts **106** and **107** showed better cytotoxic activity against all cell lines when compared to homodimer **105** [30].

**Scheme 24:**
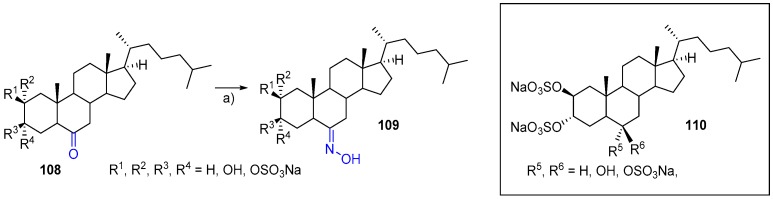
Synthesis of (6*E*)-hydroximinosteroids. Reagents and conditions: **a**) NH_2_OH·HCl, NaOAc·3H_2_O, EtOH, rt.

Richmond et al. reported the synthesis of four new (6*E*)-hydroximinosteroids (**109**), starting from the corresponding ketones (**108**) derived from cholesterol. The authors evaluated the cytotoxicity of all the prepared compounds (**109**) and compared the results to those of five polyhydroxylated sulfated analogs (**110**) (Scheme 24) [31].

Upon evaluation of the cytotoxic activity of the steroidal oxime **109** against two prostate carcinoma cell lines (PC-3 and LNCaP), the authors concluded that oxime **109** (R^1^ = R^4^ = OH, R^2^ = R^3^ = H) was the most active compound for PC-3, while for LNCaP the trisulfated analog **110** (R^5^ = H, R^6^ = OSO_3_Na) was the most active one [31].

**Scheme 25:**
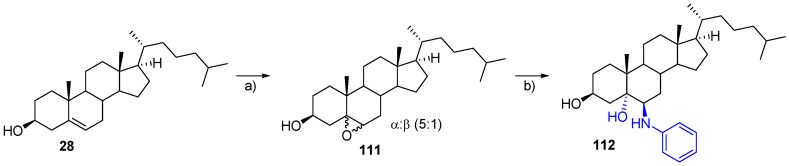
Synthesis of 6β-phenylaminocholestan-3β,5α-diol. Reagents and conditions: **a**) *m*-CPBA, CH_2_Cl_2_, 30 min; **b**) ZrO_2_/SO_4_^2−^ (50% *w*/*w*), aniline, 120 °C, 6 h.

A new greener methodology involving steroidal epoxides as intermediates for the synthesis of steroidal β-aminoalcohols was recently reported. The synthesis of β-aminoalcohol **112** involved two steps: i) Epoxidation of cholesterol **28** conducted by *m*-chloroperoxybenzoic acid (*m*-CPBA); and ii) solvent-free aminolysis of epoxide **111** mediated by sulfated zirconia (Scheme 25) [32].

The antiproliferative activity of the cholesterol-based β-aminoalcohol **112** was evaluated using MCF-7 cells, and the results showed better cytotoxic effects than cholesterol **28** itself, either by crystal violet staining (CVS) or 3-(4,5-dimethylthiazo-2-yl)-2,5-diphenyltetrazolium bromide (MTT) assays. Furthermore, cell images obtained by Harris’ hematoxylin and eosin staining protocol evidenced formation of apoptotic bodies because of the presence of cholesterol β-aminoalcohol **112** in a dose-dependent fashion [32].

**Scheme 26:**
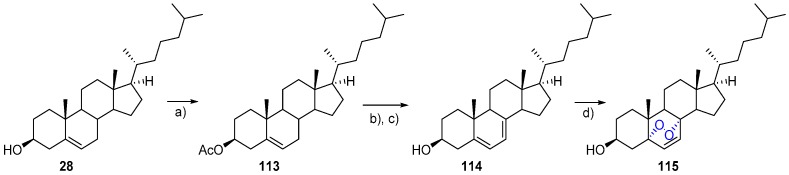
Synthesis of cholesterol-based 5α,8α-endoperoxide. Reagents and conditions: **a**) Ac_2_O, CH_2_Cl_2_, pyridine, rt, 6 h; **b**) 1. *N*-bromosuccinimide (NBS), NaHCO_3_, cyclohexane, reflux, 1 h; 2. TBAB, tetrabutylammonium fluoride (TBAF), THF, 0 °C, 4 h; **c**) NaOMe, MeOH, rt, 12 h; **d**) O_2_, eosin Y, pyridine, hv, 0 °C, 30 min.

The synthesis of new steroidal 5α,8α-endoperoxides starting from different steroids, including cholesterol, was reported, involving a four-step synthetic protocol. It involved the introduction of a diene in the cholesterol **28** structure through allylic bromination followed by elimination, and finally a photoinduced formation of the cholesterol-based 5α,8α-endoperoxide **115** (Scheme 26) [33].

The authors evaluated the in vitro antiproliferative activities of the 5α,8α-endoperoxides against human cancer cell lines derived from various human cancer types, such as human hepatocellular cancer cell lines (HepG2, SK-Hep1) and human breast cancer cell lines (MDA-MB-231, MCF-7). It was found that some compounds exhibited potent anticancer activities through inducing cancer cell apoptosis against the four tested cancer cell lines, particularly the cholesterol-based 5α,8α-endoperoxide **115**, which was the most promising derivative, presenting IC_50_ values ranging from 8.07 to 12.25 μM [33].

**Scheme 27:**
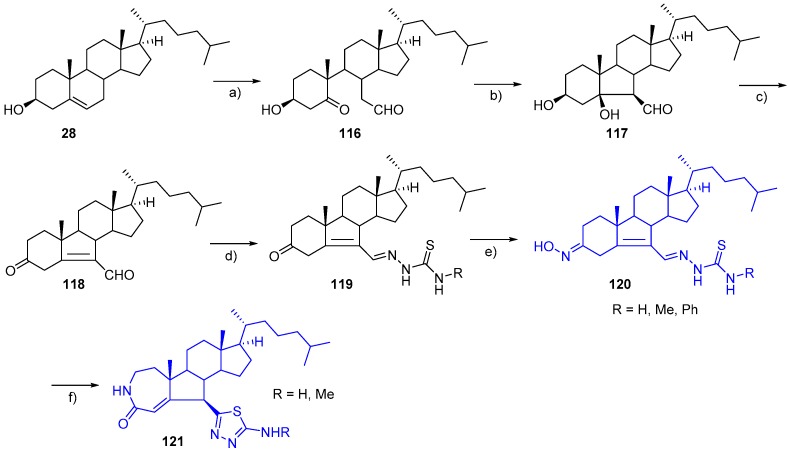
Synthesis of steroidal thiadiazoles. Reaction conditions: **a**) O_3_/(CH_3_)_2_S; **b**) Al_2_O_3_/Ph; **c**) Jones reagent, acetone, rt; **d**) NH_2_NHCSNHR, EtOH, AcOH, 80 °C; **e**) NH_2_OH·HCl, NaOAc, EtOH, 60 °C; **f**) SOCl_2_, THF, 0 °C.

A six-step synthetic route based on cholesterol **28** as a starting material was designed to prepare two new steroidal thiadiazole derivatives, **121** (R = H, Me), with an A-homo lactam and a B-norsteroidal skeleton (Scheme 27) [34]. The antiproliferative activity of compounds **118**–**121** against various cancer cell lines was evaluated, and the results showed that compounds **120** (R = Ph) and **121** (R = Me) displayed excellent selective inhibition to the A-549 (human lung carcinoma) cell line, with IC_50_ values of 7.8 and 8.0 µM, respectively [34].

**Scheme 28:**
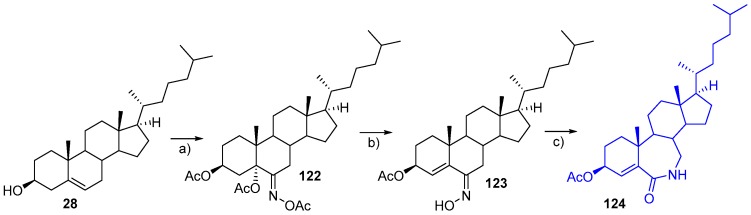
Synthesis of 6a-aza-b-homo lactams. Reagents and conditions: **a**) NaNO_2_, Ac_2_O, BF_3_OEt_2_, AcOH, rt, 1 h; **b**) Na_2_CO_3_, CH_2_Cl_2_/MeOH (1:1), reflux, 2.5 h; **c**) SOCl_2_, dioxane, rt, 20 min.

In 2017, Martínez-Pascual et al. reported a new three-step method for the synthesis of 6a-aza-b-homo steroidal lactam **124** using cholesterol **28** as a starting material. This new methodology involved the formation of a hydroximino intermediate **123** obtained in a two-step sequence from the starting cholesterol **28** (Scheme 28) [35].

The new compound **124** was evaluated as an antiproliferative agent against six human solid tumor cell lines, displaying only moderate activity against the screened cell lines [35].

**Scheme 29:**
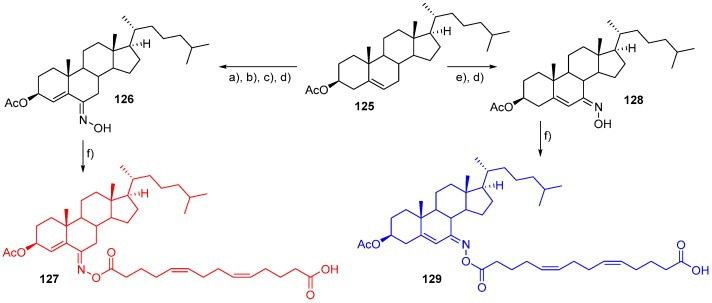
Synthesis of cholesterol-based (5*Z*,9*Z*)-dienoic acids. Reagents and conditions: **a**) *m*-CPBA, CH_2_Cl_2_; **b**) CrO_3_, H_2_O; **c**) SOCl_2_, pyridine; **d**) NH_2_OH·HCl, pyridine; **e**) celite, pyridinium dichromate (PDC), *t*-BuOH, benzene; **f**) (5*Z*,9*Z*)-tetradeca-5,9-dienedioic acid, DMAP, EDCl, CH_2_Cl_2_, rt, 12 h.

D’yakonov et al. synthesized two new hybrid compounds based on cholesterol and 1,14-tetradeca-(5*Z*,9*Z*)-dienedicarboxylic acid, **127** and **129**, which were synthetic analogues of natural (5*Z*,9*Z*)-dienoic acids. The synthetic methodology relied on the preparation of cholesterol-based oximes **126** and **128** and their further esterification using 1,14-tetradeca-(5*Z*,9*Z*)-dienedicarboxylic acid (Scheme 29) [36]. The authors evaluated the in vitro cytotoxic activities of the synthesized compounds **126**–**129** against Jurkat (leukemia), K562 (myelogenous leukemia), U937 (lung), HeLa (cervical), and Hek293 (kidney) human cell lines. The results showed that the hybrid molecules **127** and **129** efficiently induced apoptosis of the studied cell lines and were substantially more cytotoxic than their cholesterol oxime precursors **126** and **128** [36].

**Scheme 30:**
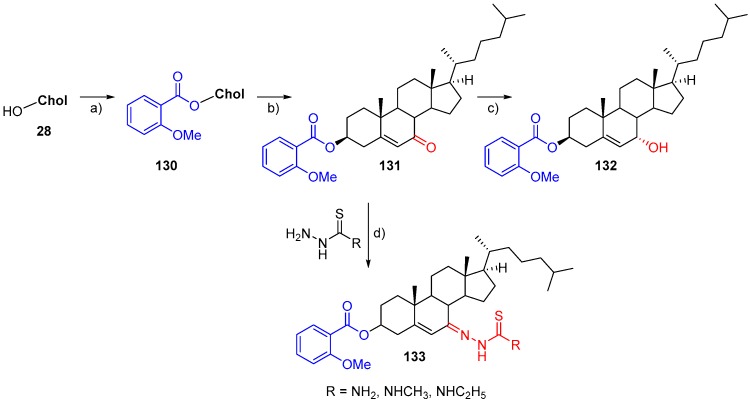
Synthesis of cholesteryl 2-methoxybenzoates. Reagents and conditions: **a**) 2-methoxybenzoyl chloride, pyridine, rt, 12 h; **b**) CrO_3_, pyridine, CH_2_Cl_2_, rt, 24 h; **c**) CeCl_2_, NaBH_4_, EtOH/CH_2_Cl_2_, rt, 30 min; **d**) EtOH, AcOH, 80 °C.

Cholesterol **28** was used as a template for the synthesis of a series of 2-methoxybenzoate analogs, bearing function groups such as carbonyl **131**, hydroxyl **132**, and thiosemicarbazones **133**, which were evaluated as potential new anticancer agents. The synthetic route involved the reaction of cholesterol **28** with 2-methoxybenzoyl chloride and the subsequent functionalization of the 7 position of the steroid core with several functional groups (Scheme 30) [37].

All of the synthesized cholesterol derivatives were evaluated for their in vitro antiproliferative activities against CNE-2 (nasopharyngeal), BEL-7402 (liver), HepG2 (liver), and Skov3 (ovarian) human cancer cells, as well as HEK-293T human kidney epithelial cells. The results demonstrated that the presence of the 7-hydroxy group (compound **132**) doubled the antiproliferative activity over the nonhydroxylated compound **130**. Furthermore, none of the evaluated compounds showed inhibitory activity on HEK-293T normal cells, making them good candidates for cancer treatment [37].

**Scheme 31:**
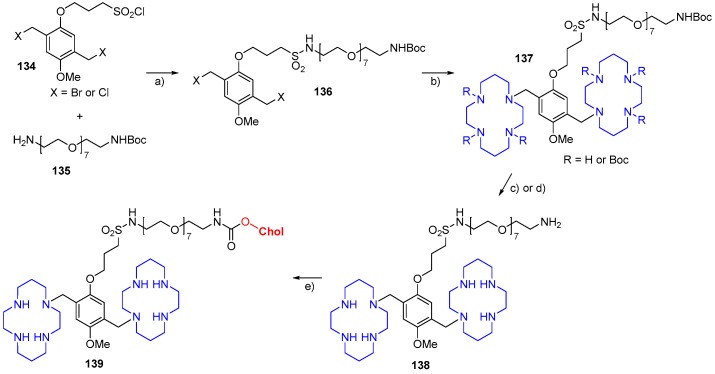
Synthesis of bis(cyclam)-capped cholesterol lipid. Reagents and conditions: **a**) DIPEA, sonication for 30 min, then DMF, rt, 30 min; **b**) (Boc)_3_cyclam or cyclam, NaHCO_3_, MeCN, reflux, 48 h; **c**) TFA, trifluoroacetic anhydride (TFAA), CH_2_Cl_2_, rt, 4 h; **d**) HCl, MeCN, rt, 25 min; **e**) cholesteryl 3β-(*N*-hydroxysuccinimidyl) carbamate, NaHCO_3_, MeCN, 75 °C, overnight.

The synthesis of a bis(cyclam)capped cholesterol lipid (**139**) was recently reported by Peters and coworkers, who also evaluated its bioactivity using primary chronic lymphocytic leukemia (CLL) cells. The synthesis of the bis(cyclam)capped cholesterol lipid relied on a four-step methodology, as depicted in Scheme 31 [38]. It was found that the bis(cyclam)capped cholesterol lipid **139** was water-soluble and self-assembled into micellar and nonmicellar aggregates in water. The authors also found that the bis(cyclam)capped cholesterol lipid **139** was as effective as the commercial drug AMD3100 in reducing chemotaxis along CXCL12 gradients, showing that **139** may be effective in disrupting the migration of CLL cells into protective niches such as the bone marrow and lymphoid organs [38].

**Scheme 32:**
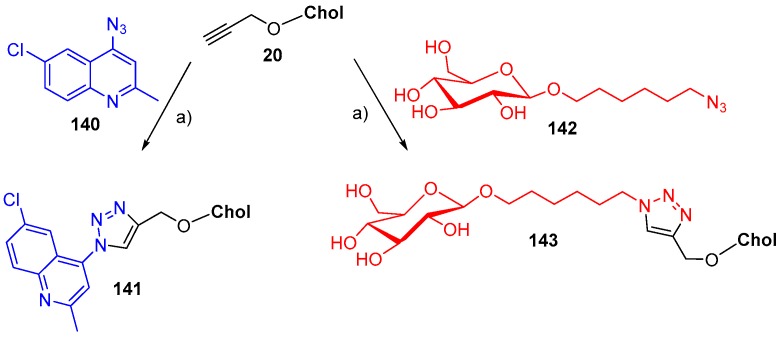
Synthesis of pharmacophoric motifs by copper-catalyzed 1,3-dipolar cycloaddition (CuAAC). Reaction conditions: **a**) CuSO_4_·5H_2_O, l-AA, THF/H_2_O (4:1), reflux.

In 2015, a paper describing the synthesis, as well as the antimicrobial and cytotoxic activities, of ten pharmacophoric motifs through CuAAC of chloroquinoline and glucose azide substrates with propargyl compounds such as chalcones, theophylline, and cholesterol was published. Within the scope of this review, only the synthesis of cholesterol-based derivatives **141** and **143** is presented (Scheme 32). Interestingly, the results from the antimicrobial evaluation showed that among the ten synthesized conjugates, triazole **143** exhibited the highest antibacterial activity against *E. coli* and *S. aureus*, and moderate antifungal activity against *A. flavus* and *C. albicans*. Furthermore, the sugar-cholesterol conjugate **143** displayed the best in vitro cytotoxic activity against the prostate cancer PC3 cell line [39].

**Scheme 33:**
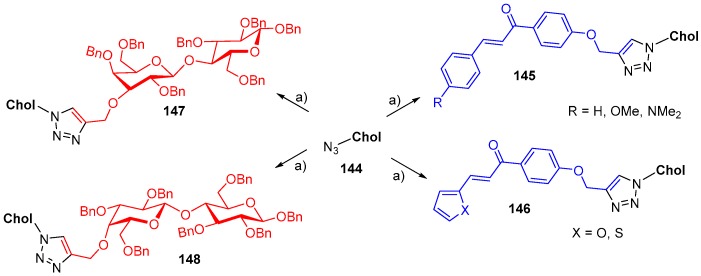
Synthesis of sugar or chalcone-triazole cholesterol conjugates. Reagents and conditions: **a**) propargyl chalcone or sugar, CuSO_4_·5H_2_O, L-AA, THF/H_2_O (5:1), reflux, 3 h.

Two cholesterol derivatives (3β-azidocholest-5-ene (**144**) and (3β)-3-(prop-2-yn-1-yloxy)- -cholest-5-ene (**20**)) were used as starting materials for the preparation of three-motif pharmacophoric conjugates including cholesterol, 1,2,3-triazole, and either a chalcone, a lipophilic residue, or a carbohydrate tag [40]. The first set of cholesterol conjugates was prepared through the reaction of 3β-azidocholest-5-ene **144** with propargylated chalcones or lactose derivatives under CuAAC conditions, affording chalcone conjugates **145** and **146** and lactose conjugates **147** and **148** (Scheme 33) [40].

**Scheme 34:**
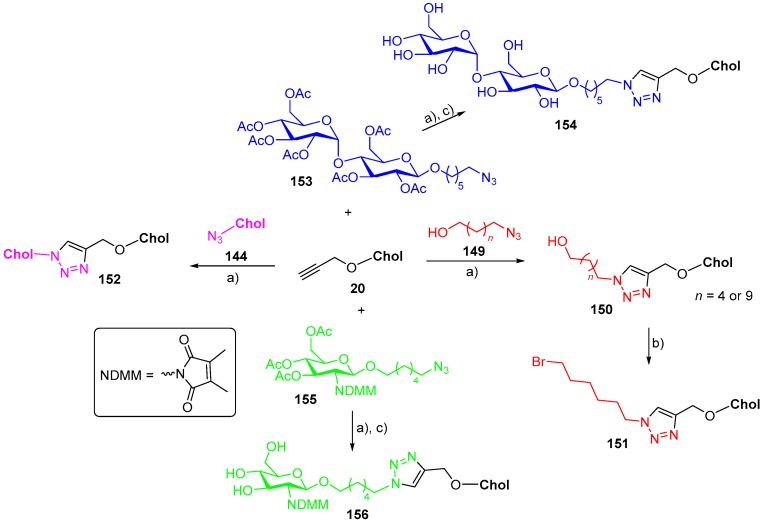
Synthesis of cholesterol-triazole dimer, cholesterol-triazole alkanes, and cholesterol-triazole carbohydrates. Reagents and conditions: **a**) CuSO_4_·5H_2_O, l-AA, THF/H_2_O (5:1), reflux, 3 h; **b**) CBr_4_, PPh_3_, CH_2_Cl_2_, rt, overnight; **c**) NaOMe, MeOH, rt, 2 h.

A second set of cholesterol conjugates was prepared once again through CuAAC of (3β)-3-(prop-2-yn-1-yloxy)cholest-5-ene (**20**) with azido alkanols (**149**) and 3β-azidocholest-5-ene (**144**), affording cholesterol-triazole alkanols (**150**) and a triazole-linked cholesterol dimer (**152**), respectively (Scheme 34) [40]. Furthermore, compound **150** was converted in the respective bromo alkane **151** through a substitution reaction in the presence of CBr_4_ (Scheme 34) [40]. A carbohydrate-tagged set of cholesterol compounds was prepared by the CuAAC reaction of (3β)-3-(prop-2-yn-1-yloxy)- -cholest-5-ene (**20**) with the appropriate glycosyl azides **153** and **155**, affording compounds **154** and **156**, respectively, upon cleavage of the acetyl protecting groups (Scheme 34) [40].

**Scheme 35:**
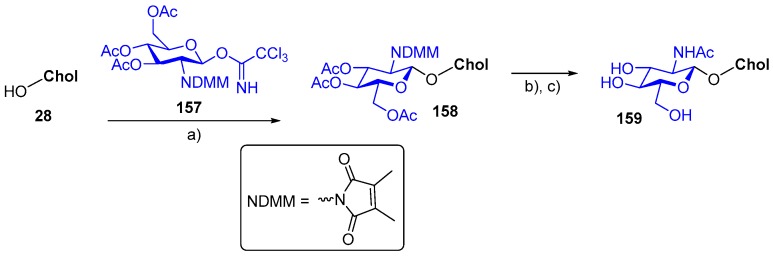
Synthesis of carbohydrate-tagged cholesterol conjugates. Reagents and conditions: **a**)TMSOTf, MeCN, rt, 15 min; **b**) NaOMe, MeOH, rt, 75 min; **c**) 1. NaOH, 1,4-dioxane/MeOH/H_2_O (6:3:1), rt, 8 h then HCl (pH 5), rt, overnight; 2. Ac_2_O/pyridine (1:2), rt, overnight; 3. NaOMe, MeOH, rt, 2 h.

Another carbohydrate-tagged compound, **159**, was synthesized through the reaction of cholesterol **28** with an appropriate glycosyl donor **157** in a three-step protocol, as depicted in Scheme 35 [40]. The authors screened all the cholesterol conjugates for their in vitro antimicrobial and anticancer activities. Among all compounds, the chalcone-triazole-cholesterol derivative **145** (R = NMe_2_) was the one with the most promising antimicrobial activity, being as active as the controls against *E. coli*, *S. aureus* and *C. albicans*. Concerning the cytotoxic potential of the cholesterol conjugates, the cholesterol-triazole-lactoside congener **147** displayed the best in vitro cytotoxic effect against the prostate cancer PC3 cell line, with similar cytotoxicity to that of DOX, used as a control [40].

**Scheme 36:**
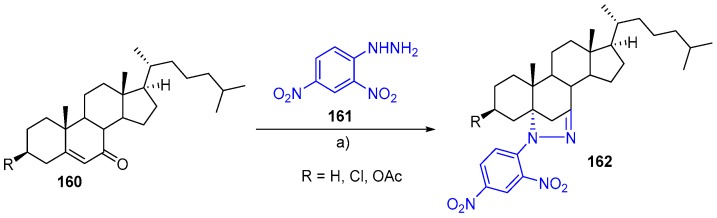
Synthesis of steroidal pyrazolines. Reagents and conditions: **a**) DMSO, AcOH, reflux, 21–35 h.

A new methodology for the synthesis of steroidal pyrazolines (**162**) through the reaction of cholest-5-en-7-ones (**160**) with 2,4-dinitrophenylhydrazine (**161**) was reported by Shamsuzzaman and coworkers in 2016 (Scheme 36) [41]. The reaction proceeded by a well-known 1,4-/1,2-addition/dehydration mechanism to an α,β-unsaturated carbonyl compound. The new steroid-based pyrazolines (**162**) were evaluated for their in vitro antibacterial activity against three different strains (*E. coli*, *Corynebacterium xerosis*, and *S. epidermidis*), in which compound **162** (R = H) was the most active against *C. xerosis* and *S. epidermidis*, with minimum inhibitory concentrations similar to the positive control gentamicin. Compound **162** (R = H) also demonstrated moderate activity against fungal strains *Mucor azygosporus*, *Claviceps purpurea*, and *A. niger*, being the most effective compound tested. The in vitro anticancer activity against five human cancer cell lines (SW480 (colon), HeLa (cervical), A549 (lung), HepG2 (hepatic), HL-60 (leukemia)) of pyrazolines (**162**) was also screened, with the chlorinated compound **162** (R = Cl) the most active [41].

**Scheme 37:**
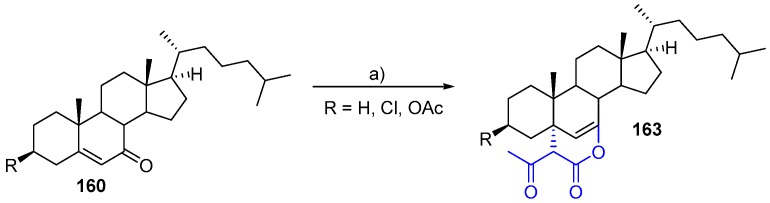
Synthesis of steroidal 2*H*-pyran-2-ones. Reaction conditions: **a**) ethyl acetoacetate, chitosan, MeOH, reflux, 13–16 h.

The same research group reported a green simple synthesis of steroidal 2*H*-pyran-2-ones (**163**), starting from 3-substituted cholest-5-en-7-ones (**160**) and ethyl acetoacetate in the presence of chitosan as an ecofriendly heterogeneous catalyst (Scheme 37) [42]. The synthesized steroidal 2*H*-pyran-2-ones (**163**) were tested in vitro against two cancer cell lines (HeLa (cervical) and Jurkat (leukemia)) and one normal cell line (PBMC: Peripheral blood mononuclear cell). All the tested compounds (**163**) exhibited moderate-to-good activity against the two human cancer cell lines and were less toxic against the noncancer cell line. Furthermore, the antioxidant potential of these new compounds (**163**) was also evaluated, exhibiting lower 2,2-diphenyl-1-picrylhydrazyl radical (DPPH) radical scavenging activity than the positive control, ascorbic acid [42].

**Scheme 38:**
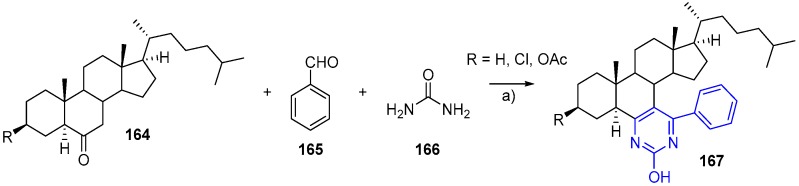
Synthesis of steroidal pyrimidines. Reagents and conditions: **a**) trimethylsilyl chloride (TMSCl), DMF/MeCN (1:2), 90 °C, 13–14.5 h.

A series of new steroidal pyrimidine derivatives (**167**) was prepared through the multicomponent reaction of cholestan-6-ones (**164**) with urea (**166**) and benzaldehyde (**165**) in the presence of trimethylsilyl chloride (TMSCl) as catalyst (Scheme 38) [43]. The antitumor activity of these steroidal pyrimidine-functionalized scaffolds (**167**) was screened against three human cancer cell lines, MDA-MB231 (breast), HeLa (cervical), and HepG2 (hepatic), and one noncancer normal cell line, PBMC, by MTT assay. All tested compounds showed cytotoxicities against the three cancer cell lines. Particularly, compound **167** (R = H) exhibited the highest cytotoxicity against the three cancer cell lines. However, all cases were lower than DOX, used as a positive control [43]. The authors also addressed the antioxidant activity of the pyrimidine compounds (**167**), concluding that these new compounds presented reduced DPPH radical, hydroxyl radical, nitric oxide radical, and H_2_O_2_ scavenging potential than l-ascorbic acid, used as a control. Moreover, the IC_50_ values pointed out that the scavenging activity of the tested compounds were in the order of nitric oxide radical < hydrogen peroxide < DPPH radical < hydroxyl radical [43].

**Scheme 39:**
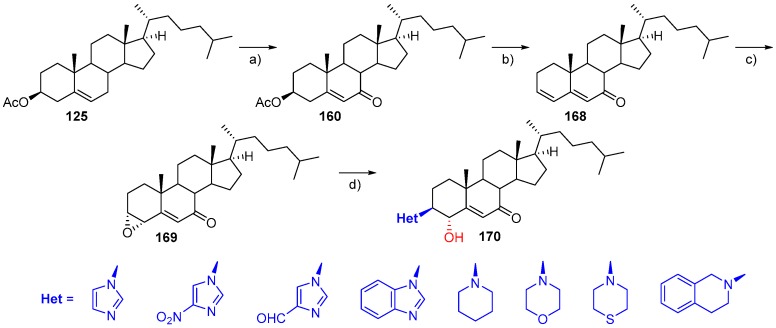
Synthesis cholesterol-based *N*-heterocycles. Reagents and conditions: **a**) *t*-BuOOH, RuCl_3_, cyclohexane, rt, 4 h; **b**) HCl, MeOH, reflux, 1 h; **c**) *m*-CPBA, CHCl_3_, rt, 5 h; **d**) *N*-heterocycle, neat, MW, 140 °C, 6–16 min.

The cases in which the attachment of a heterocycle in the steroid backbone changes the biological properties of the steroid molecule are not so rare, and often are an interesting platform for the development of new pharmacophores. In this context, Saikia et al. reported the synthesis of steroidal heterocyclic compounds (**170**) through the solvent-free microwave-assisted epoxide ring opening with nitrogen nucleophiles [44]. The first series of *N*-heterocycles was synthesized by the reaction of nitrogen nucleophiles with the epoxide **169**, which was prepared in a three-step synthetic route starting from cholesterol acetate **125** (Scheme 39) [44].

**Scheme 40:**
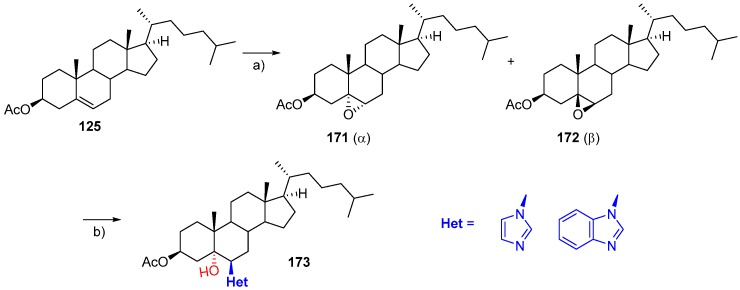
Synthesis cholesterol-based *N*-heterocycles. Reagents and conditions: **a**) *m*-CPBA, CHCl_3_, rt, 5 h; **b**) imidazole or benzimidazole, neat, MW, 140 °C, 10 min, recrystallization.

The synthesis of another set of *N*-heterocycles, **173**, was accomplished using a mixture of epoxides (**171** (α) and **172** (β) (4:1)) as starting materials, which were obtained through the direct epoxidation of cholesterol acetate **125** (Scheme 40) [44]. It is worth noticing that compound **173** was obtained as a diastereomeric mixture, which upon recrystallization in ethanol provided pure alcohol **173**.

**Scheme 41:**
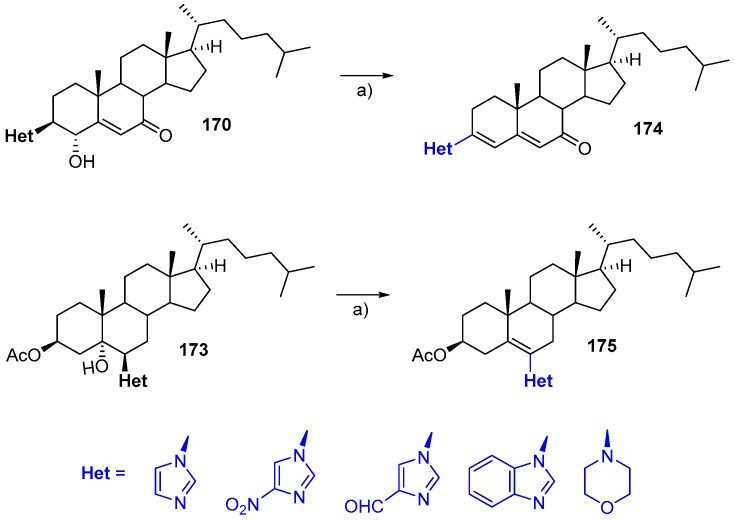
Dehydration of cholesterol-based *N*-heterocycles. **a**) H_2_SO_4_ (cat.), AcOH, 80 °C, 4–6 h.

The authors also considered the dehydration of the obtained cholesterol-based *N*-heterocycles (**170** and **173**), which was successfully accomplished using a catalytic amount of sulfuric acid in acetic acid, affording compounds **174** and **175**, respectively (Scheme 41) [44].

Finally, the in vitro antibacterial activity of all compounds was evaluated, and the *N*-heterocycles **170** (Het = 4-nitroimidazole, piperidine, morpholine, thiomorpholine, tetrahydroisoquinoline) and dehydrated *N*-heterocycles **174** (Het = 4-nitroimidazole, morpholine) demonstrated moderate effects against the tested microorganisms (*E. coli*, *P. syringae*, *B. subtilis*, *P. vulgaris* and *S. aureus*). Specifically, compound **170** (Het = piperidine, morpholine, and thiomorpholine) inhibited all the tested strains, and the **170** (Het = tetrahydroisoquinoline) derivative showed inhibition against three gram-negative bacterial strains, *E. coli*, *P. syringae*, and *P. vulgaris*. The authors also concluded that the removal of the hydroxyl group decreased the antimicrobial activity of the tested compounds [44].

**Scheme 42:**
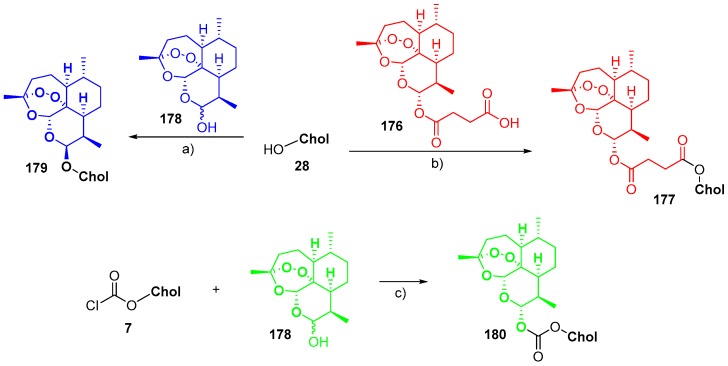
Synthesis of *O*-linked artemisinin-cholesterol conjugates. Reagents and conditions: **a**) BF_3_·OEt_2_, CH_2_Cl_2_, 0 °C to rt, overnight; **b**) DCC, DMAP, CH_2_Cl_2_, rt, 4 h; **c**) DMAP, Et_3_N, CH_2_Cl_2_, rt, 18 h.

Recently, Morake and coworkers synthesized a series of artemisinin-cholesterol conjugates, **177**, **179**, **180**, **182**, **184**, **186**, and **188**, expecting that the putative cholesterol transporters may enhance the activity of the parent drug (artemisinin) against malaria and tuberculosis [45]. The conjugates were designed to have different *O*- or *N*-linkers, such as ether, ester, and carbamate, varying the length of each linker as well. The first set of conjugates, **177**, **179**–**180**, was synthesized from cholesterol **28** or cholesteryl chloroformate **7** with dihydroartemisinin **178** or artesunate **176** (Scheme 42) [45].

**Scheme 43:**
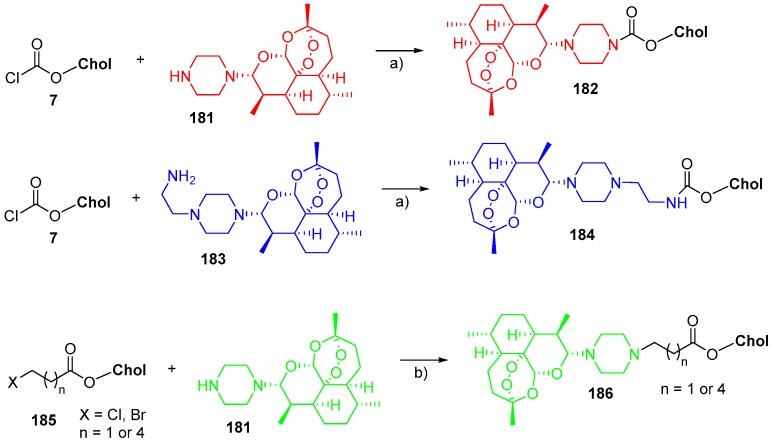
Synthesis of *N*-linked artemisinin-cholesterol conjugates. Reagents and conditions: **a**) Et_3_N, CH_2_Cl_2_, rt, overnight; **b**) Et_3_N, THF, reflux, 24 h.

A second set of conjugates, **182**, **184**, and **186**, was synthesized starting from a specific artemisinin derivative, **181** or **183**, appropriately substituted with a piperazine group at C-10, through reaction with the appropriate cholesterol derivative, **7** or **185** (Scheme 43) [45].

**Scheme 44:**
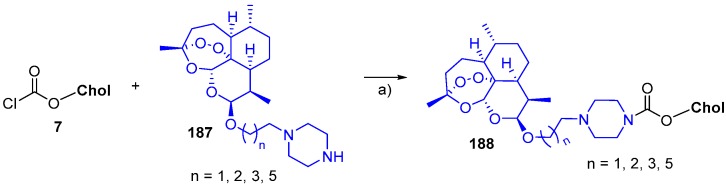
Synthesis of *O*-linked artemisinin cholesteryl carbamates: Reagents and conditions: **a**) Et_3_N, CH_2_Cl_2_, 0 °C to rt, overnight.

Furthermore, the authors designed a final set of compounds bearing a carbamate linker, **188**. The synthesis of this set of compounds was carried out through an amidation reaction of cholesteryl chloroformate **7** with the appropriate amine derivative, **187**, bearing different lengths of alkyl chains (Scheme 44) [45].

The antimalarial activity of the novel artemisinin-cholesterol conjugates **177**, **179**, **182**, **184**, **186**, and **188** were evaluated against *Plasmodium falciparum* (*Pf*) NF54, K1, and W2 strains, in which the conjugates of **186** (*N*-linked artemisinin-cholesterol conjugates) were the most active derivatives. However, the potency of these compounds was lower than the precursors artemether and artesunate. The authors rationalized these results based on the low solubility in the culture medium given by cholesterol moiety, which may have affected the efficacies of the artemisinin-cholesterol conjugates. On the other hand, concerning the activities against *Mycobacterium tuberculosis* (*Mtb*) H37Rv, the conjugates displayed enhanced efficacy over the parent drug artemisinin [45].

**Scheme 45:**
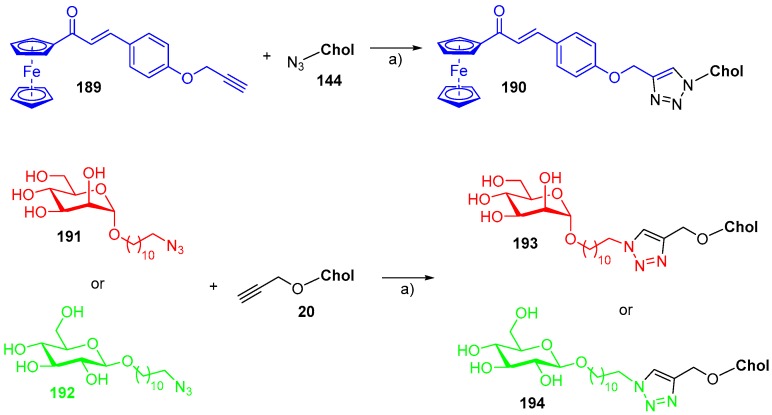
Synthesis of cholesterol-triazole conjugates bearing ferrocene-chalcone and sugar moieties. Reagents and conditions: **a**) l-AA, CuSO_4_·5H_2_O, THF/H_2_O (6:1), 60–80 °C, 4 h.

The synthesis of three new cholesterol conjugates, **190**, **193**, and **194**, via CuAAC reaction was recently reported [46]. These conjugates were prepared either to have a ferrocene-chalcone moiety **190** or sugar moieties **193** and **194** as well, both linked by a triazole group (Scheme 45) [46].

The antimicrobial activities of these cholesterol conjugates were evaluated in vitro against *E. coli*, *S. aureus*, *A. flavus*, and *C. albicans*. Surprisingly, the authors found that the cholesterol conjugate bearing ferrocene-chalcone moiety **190** was completely inactive against all the tested bacteria. On the other hand, sugar conjugates **193** and **194** showed moderate inhibitory activity against *E. coli*, *A. flavus*, and *C. albicans*, being even less potent than control compounds ampicillin and amphotericin B [46].

**Scheme 46:**
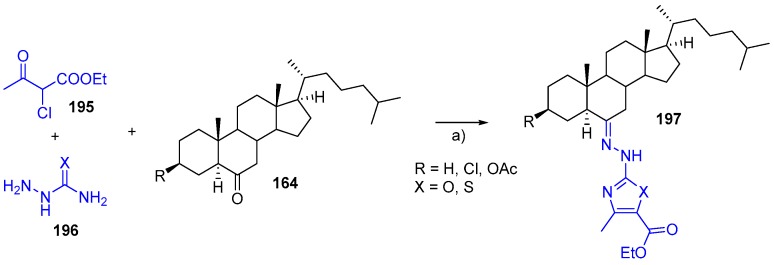
Synthesis of steroidal oxazole/thiazole derivatives. Reagents and conditions: **a**) NaOAc, EtOH, reflux, 6–10 h.

Employing a one-pot multicomponent reaction procedure using (thio)semicarbazide hydrochloride **196** and ethyl 2-chloroacetoacetate **195** allowed the preparation of a series of steroidal oxazole and thiazole derivatives (**197**) (Scheme 46) [47].

The antimicrobial activity of the new steroidal compound **197** was evaluated against two gram-negative (*E. coli* and *P. aeruginosa*) and two gram-positive bacterial strains (*S. aureus* and *L. monocytogenes*). Additionally, the bioactivity against pathogenic fungi (*C. albicans* and *C. neoformans*) was also addressed. The authors found that most of the compounds exhibited good antibacterial and antifungal activity against the tested strains. In addition, the compounds also showed interesting antibiofilm activity against *S. aureus* biofilm. Molecular docking studies showed effective binding of the steroidal compound **197** with amino acid residues of DNA gyrase and glucosamine-6-phosphate synthase through hydrogen bonding interactions [47].

**Scheme 47:**
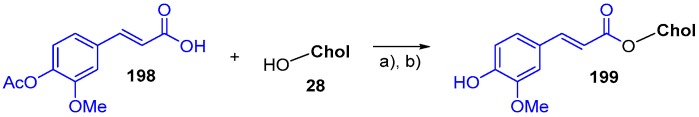
Synthesis of cholesterol-based steryl ferulate. Reagents and conditions: **a**) DCC, DMAP, CH_2_Cl_2_, MW, 100 °C, 15 min; **b**) K_2_CO_3_, CHCl_3_/MeOH (2:1), reflux, 6 h.

Given the increasing importance of steryl ferulates [3-*O*-(*trans*-4-feruloyl)sterols] in pharmaceutical applications, Begum and coworkers reported the microwave-assisted synthesis of steryl ferulates from several steroids [48]. The synthesis of cholesterol-based steryl ferulate **199** is exemplified in Scheme 47, in which microwave (MW) irradiation played a crucial role in the esterification step with *trans*-4-*O*-acetylferulic acid **198** [48].

The authors evaluated the antioxidant capacity (DPPH radical scavenging, total antioxidant capacity, and reducing power) of all synthesized steryl ferulates in comparison to equimolar mixtures of steryl ferulates and γ-oryzanol (a natural mixture of steryl ferulates, abundant in cereal bran layers). The results showed that the mixture of steryl ferulates and γ-oryzanol was a better radical scavenger than most individual ferulates, including the cholesterol-based one, **199** [48].

## 4. Cholesterol-Based Liquid Crystals

A liquid crystal is basically a state of matter that has properties between those of conventional liquids and those of solid crystals. The classification of liquid crystals was proposed in the 19th century and is based on molecular arrangement. Since then, liquid crystals have been divided into smectic (from the Greek word “smegma”, meaning soap) and nematic (from the Greek word “nema”, meaning thread) crystals. In smectic liquid crystals, molecules are arranged so that their major axes are parallel, and their centers of mass lie in one plane. There are many different smectic phases characterized by different types and degrees of positional and orientational order. The most common ones are the smectic A phase, in which the molecules are oriented along the layer normal, and the smectic C phase, in which the molecules are tilted away from it. Nematic phases are the simplest liquid crystalline phases formed, since they only have long-range orientational order (of, e.g., molecules, columns) and no degree of long-range translational order [49]. There is also a chiral variant of nematic or smectic phases, when the molecules of the liquid crystalline substance are chiral, with these phases denoted N* or Sm(A/B)* (an asterisk denotes a chiral phase), respectively. These phases are often called the cholesteric phases, because they were first observed for cholesterol derivatives [49].

**Scheme 48:**
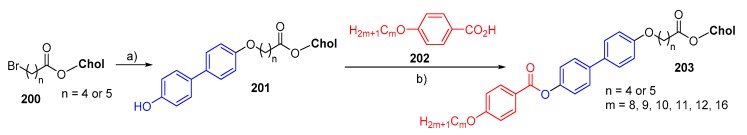
Synthesis of cholesterol-biphen-4-yl 4-(*n*-alkoxy)benzoate conjugates. Reagents and conditions: **a**) bisphenol, K_2_CO_3_, DMF, 85 °C, 24 h; **b**) DCC, DMAP, CH_2_Cl_2_, 40 °C, 24 h.

In 2014, Hiremath reported the synthesis of two new series of cholesterol-biphen-4-yl 4-(*n*-alkoxy)benzoate conjugates (**203**), linked through either odd-parity or even-parity spacers (Scheme 48) [50]. The compounds in **203** are optically active, and both series of conjugates show a frustrated liquid crystalline state, with a thermodynamically stable twist grain boundary phase with a chiral smectic C structure (TGBC*) over an exceedingly wide thermal range [50].

The author explained such behavior based on the combined effect of extended geometry (conformation), strong chirality, and the enantiomeric excess of the molecules. Furthermore, the conjugates of **203** with an odd-parity spacer show an additional phase, the blue one. The clearing transition temperatures and the associated enthalpies alternate where the odd members exhibit lower values compared to those of even members. These results clearly demonstrate that the geometry (rod-like and bent conformation) and the thermal behavior of the conjugates of **203** are greatly influenced by the spacer parity [50].

**Scheme 49:**
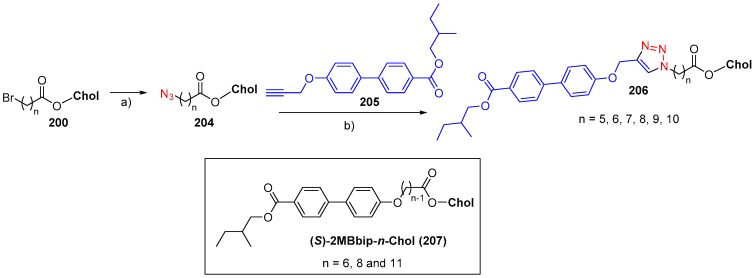
Synthesis of cholesterol-biphenylene dimers. Reagents and conditions: a) NaN_3_, DMF, 70 °C, overnight; **b**) CuI, THF, reflux, 6 h.

A series of similar conjugates of **206**, containing cholesterol, triazole, and biphenylene units, were synthesized via CuAAC chemistry (Scheme 49). Different flexible spacers were introduced in the system to evaluate the effect on the mesophase formation as well as the influence of the presence of a triazole linker [51].

The authors concluded that short (*n* = 5 and 6) and medium (*n* = 7, 8, and 9) alkyl spacers exhibit enantiotropic SmA* and monotropic SmC* phases, whereas the conjugate possessing the longest spacer (*n* = 10) favors the formation of enantiotropic SmA and N* phases. A close correlation between the transition temperatures and the increase in the length of the methylene spacer was also observed, and a higher clearing point was observed for the even spacers. Further comparison studies with **(*S*)-2MBbip-*n*-Chol 207** (Scheme 49) demonstrated that the triazole ring plays a crucial role in the mesophase formation, wherein apart from the molecular dipole, the subtle electrostatic interaction and van der Waals forces enhance the SmC* phase [51].

**Scheme 50:**
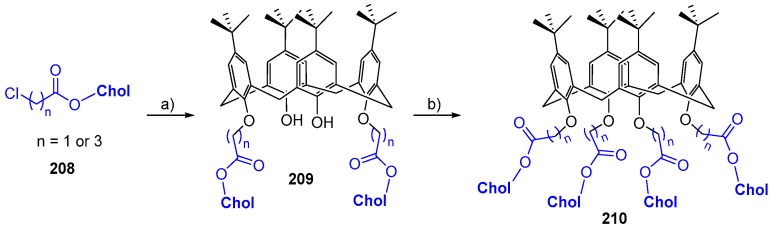
Synthesis of calix[4]arene-cholesterol derivatives. Reagents and conditions: **a**) calix[4]arene, K_2_CO_3_, KI, MeCN, reflux, 24 h; **b**) 208, K_2_CO_3_, KI, MeCN, reflux, 36 h.

A study involving the design, synthesis, and mesomorphic properties of the first examples of cholesterol-based calixarene liquid crystals was reported in 2015 by Guo and coworkers [52]. Novel cholesterol-1,3-bis-substituted calix[4]arene **209** and cholesterol-tetra-substituted calix[4]arene **210** derivatives were synthesized by reacting cholesterol-chlorinated derivatives (**208**) with calix[4]arene, as depicted in Scheme 50.

The liquid crystalline behaviors of cholesterol-calix[4]arene compounds **209** and **210** were studied, and both showed excellent mesomorphic properties of the columnar molecular arrangement of the calix[4]arene bowlic column, with cholesterol units as ancillary lateral columns. Furthermore, the authors demonstrated that compounds with longer spacers and more cholesterol units, such as **210**, are better for good mesomorphic properties [52].

**Scheme 51:**
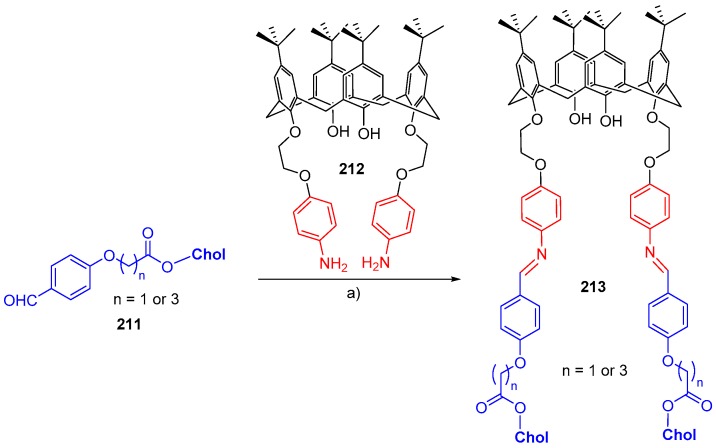
Synthesis of calix[4]arene-cholesterol derivatives with Schiff-base bridges. Reagents and conditions: **a**) AcOH, MeOH/CHCl_3_ (1:1), reflux, 8 h.

Following this study, similar calix[4]arene-cholesterol derivatives with Schiff-base bridges (**213**) were synthesized (Scheme 51), and the influence of complexation behaviors on their mesomorphic properties was investigated [53]. Like the previous cholesterol-calix[4]arene compounds (**210**), these Schiff-base bridged compounds (**213**) presented mesomorphic properties with a molecular arrangement of the calixarene bowlic column and Schiff-base cholesterol units as ancillary lateral columns as well. However, upon complexation with AgClO_4_, no mesophase was observed, suggesting that the mesomorphic properties of compound **213** could be tuned by ion-complexation behavior [53].

**Scheme 52:**
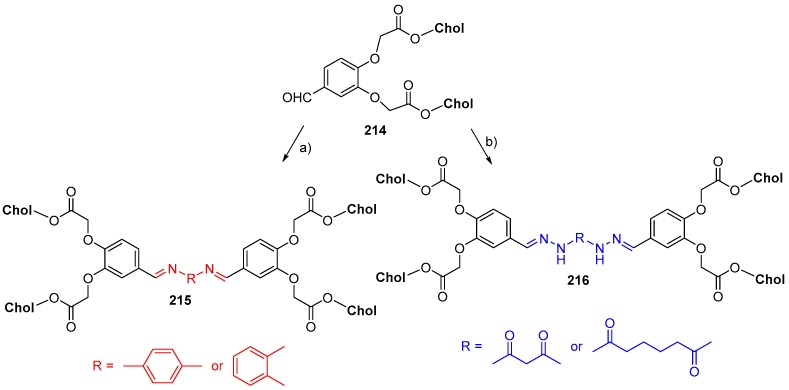
Synthesis of cholesterol tetramers with Schiff-base bridges. Reagents and conditions: **a**) *p*-phenylenediamine or *o*-phenylenediamine, AcOH, CHCl_3_/EtOH (1:4), reflux, 12 h; **b**) malonic bis-hydrazide or adipic bis-hydrazide, AcOH, CHCl_3_/EtOH (1:4), reflux, 12 h.

Recently, novel columnar liquid crystals (LCs) based on symmetric hairpin-shaped cholesterol tetramers with Schiff-base spacers were prepared, and their mesomorphic behaviors were investigated by different techniques. The new molecules were synthesized through the reaction between a cholesterol dimer, **214**, and phenylenediamines or bis-hydrazides working as spacers containing hydrogen bonds, affording compounds **215** and **216** (Scheme 52) [54].

The results indicated good hexagonal columnar liquid crystalline behaviors, with three molecules arranged as a disc of the columnar hexagonal state. In addition, the symmetric cholesterol tetramers with rigid cores or hydrogen-bonding cores strongly favored the formation of a columnar mesophase [54].

**Scheme 53:**
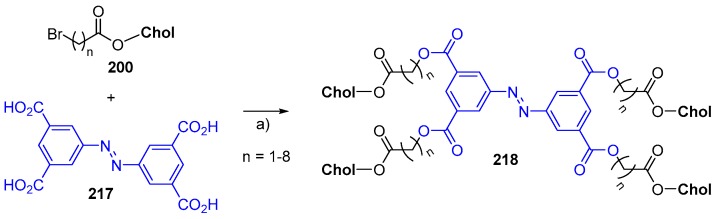
Synthesis of azobenzene-centered cholesterol-based tetramers. Reagents and conditions: **a**) KOH, H_2_O, tetraoctylammonium bromide (TOAB), reflux, 5 h.

The preparation of a series of tetramers (**218**), based on azobenzene decorated with cholesterol units, was also recently reported. These oligomeric compounds bearing different alkyl spacers were synthesized by reacting azobenzene tetracarboxylic acid (**217**) with cholesteryl derivatives (**200**) (Scheme 53) [55].

Among the synthesized compounds, it was found that oligomers with *n* = 1, 5, and 8 exhibited an enantiotropic N* phase, while the other oligomers showed a monotropic N* phase, upon cooling from an isotropic state. Interestingly, oligomers with *n* = 1 and 8 formed spherulites in their crystalline state, dispersed for hundreds of micrometers in the case of the oligomer with *n* = 1. Moreover, both oligomers (*n* = 1 and 8) had photoisomerization in dilute solutions and Langmuir monolayers, in opposition to the liquid crystalline state, in which no photoisomerization was observed [55].

**Scheme 54:**
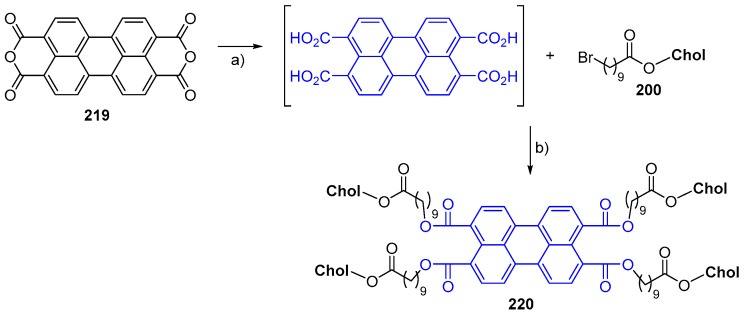
Synthesis of cholesterol-perylene-based liquid crystals (LCs). Reagents and conditions: **a**) KOH, H_2_O, 70 °C, 2 h, then HCl (aq); **b**) TOAB, reflux, 3 h.

Cholesterol-based nonconventional liquid crystals have been studied by Gupta and coworkers. They reported the synthesis of novel functional discotic oligomeric materials based on 3,4,9,10-tetrasubstituted perylene, one of which bore the cholesterol units of **220** (Scheme 54) [56].

The cholesterol derivative **220** was found to be a nonconventional LC at room temperature: However, a monotropic nematic (N*) phase on cooling was achieved. The authors also demonstrated that the combination of rod and disc-like moieties sufficiently perturbed the molecular shape to yield calamitic mesophases. Additionally, this hybrid material showed interesting fluorescence emission properties, making it suitable for a range of optoelectronic applications [56].

**Scheme 55:**
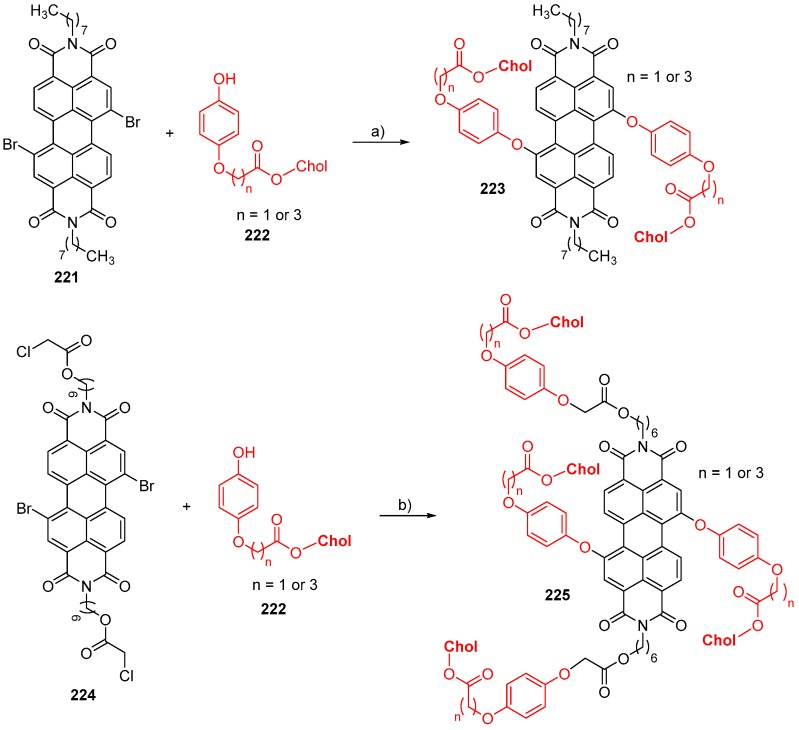
Synthesis of cholesterol-perylene liquid crystals. Reagents and conditions: **a**) K_2_CO_3_, DMF, 95 °C, 10 h; **b**) K_2_CO_3_, DMF, 105 °C, 20 h.

Recently, the synthesis of perylene derivatives with two (**223**) or four cholesterol units (**225**) at bay-position or both in bay-position and imide position, respectively, was reported (Scheme 55). The authors addressed the influence of the number as well as the position of the cholesterol units on the mesomorphic and photophysical properties of these new liquid crystals [57]. The authors concluded that more cholesterol units significantly lowered the mesophase temperature, created wider scopes of phase transfer temperatures, and increased the fluorescence. Furthermore, it was found that a longer spacer between perylene and cholesterol units was ideal for mesomorphic properties as well as to enhance the fluorescence of the compounds [57].

**Scheme 56:**
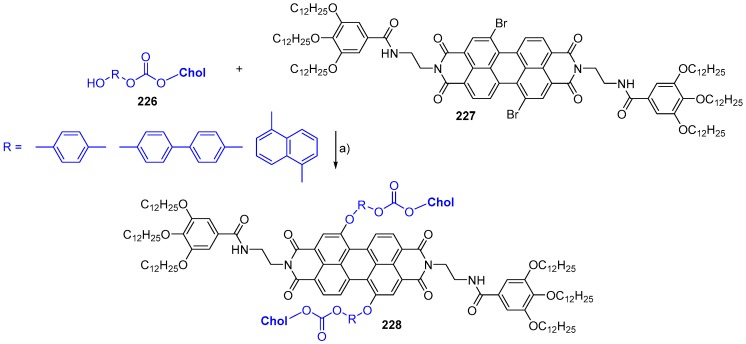
Synthesis of perylene-based liquid crystals bearing different bay-rigid spacers. Reagents and conditions: **a**) K_2_CO_3_, DMF, 100 °C, 24 h.

A year later, Chen et al. reported the synthesis of three different perylene-based liquid crystals bearing different bay-rigid spacers (**228**). These new liquid crystals were synthesized starting from a perylene derivative (**227**) with six alkyl chains on the imides positions by coupling two phenyl (biphenyl or naphthyl)-bridging cholesterol units (**226**) at bay positions (Scheme 56) [58]. Investigations addressing the mesomorphic properties of these perylene-based compounds (**228**) demonstrated that all derivatives ordered hexagonal columnar liquid crystalline behaviors, despite the functionalization of the bay positions with aromatic spacers. Derivatives with larger and rigid aromatic spacers presented higher phase transition temperatures as well as smaller scopes of mesophase temperatures. The authors also concluded that rigid and larger aromatic groups showed stronger emission and higher fluorescence quantum yield. These results suggested that by adjusting the structures of spacers on the bay position, both mesomorphic and photophysical properties are likely to be tuned depending on the purpose of the liquid crystal [58].

**Scheme 57:**
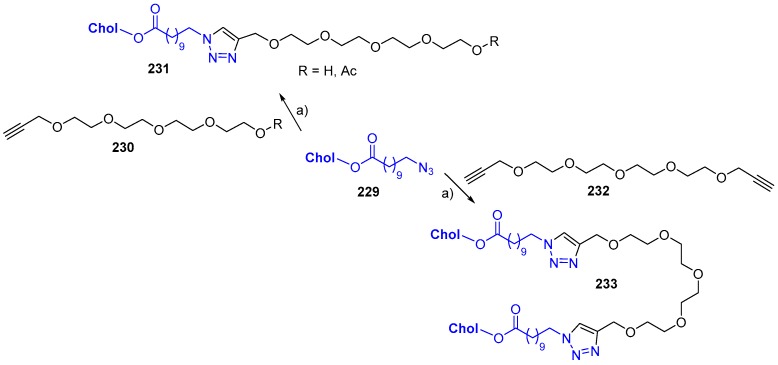
Synthesis of cholesteryl dimer and two cholesteryl monomers. Reagents and conditions: **a**) CuI, DIPEA, acetone, 55 °C, overnight.

Aiming to explore the potentially interesting mesomorphic properties of liquid crystals, Champagne and coworkers reported the synthesis of a synthetic liquid crystal dimer (**233**) and two of its monomer analogues (**231**) based on cholesterol mesogens [59]. The synthesis relied on the CuAAC reaction of a cholesteryl azide (**229**) with α,ω-di-*O*-propargyl-TEG (**232**) and *O*-monopropargylated-TEG (**230**) linkers, as depicted in Scheme 57. Several experimental studies were carried out, showing that both monomers (**231**) as well as the dimer (**233**) formed a smectic A liquid crystalline phase with comparable layer spacing. The authors explained this feature by the formation of a bilayer structure in the case of the monomers (**231**) and a monolayer structure for the dimer (**233**). Concerning the thermal stability of the self-assembled phases, the clearing temperature increased around 10 °C from **231** (R = Ac) to **231** (R = H). Molecular modeling studies rationalized the features of the liquid crystalline phases based on the different chemical functional groups present in each class of materials, allowing different kinds of intermolecular interactions, such as dipole-dipole interaction, hydrogen-bonding, as well as London dispersion forces, which greatly affected the self-assembly behavior of the three cholesterol derivatives [59].

**Scheme 58:**
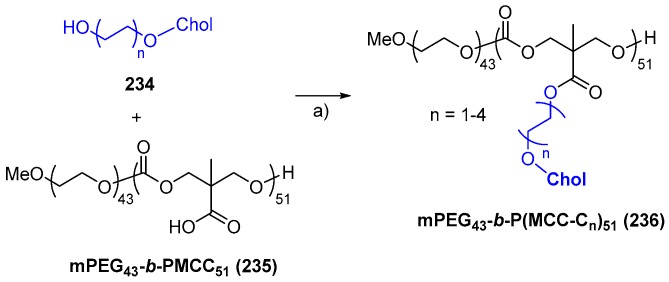
Synthesis of block copolymers **mPEG_43_-*b*-P(MCC-C_n_)_51_**. Reagents and conditions: **a**) DCC, DMAP, CH_2_Cl_2_.

The synthesis of four new aliphatic polycarbonate copolymers (**mPEG_43_-*b*-P(MCC-C_n_**)_51_ (**236**) (*n* = 1–4) containing cholesteryl groups as side chain mesogenic units was achieved through the coupling reaction between **mPEG_43_-*b*-PMCC_51_** (**235**) with a side carboxyl group and chiral cholesteryl derivatives (**234**) with different numbers of methylene groups, bearing a terminal hydroxyl group (Scheme 58) [60].

The authors studied the liquid crystal behavior of both chiral cholesteryl compounds (**234**) and the block copolymers based on cholesterol (**236**). The results demonstrated that the chiral compounds (**234**) exhibited an enantiotropic mesophase of an SmA phase and cholesteric phase except for **234** (*n* = 1), which only showed an SmA phase. The block copolymers showed an enantiotropic mesophase of an SmA phase except for **mPEG_43_-*b*-P(MCC-C_1_)_51_** (**236**) (*n* = 1), with the mesophase temperature range of the copolymers (**236**) being greater than those of the corresponding chiral compounds (**234**). It was also concluded that a longer spacer tended to stabilize the mesophase more than a shorter one and showed a wide mesophase range. These new polycarbonate copolymers with longer spacers based on cholesterol exhibited mesophase states below body temperature, which makes them good candidates for drug delivery applications [60].

**Scheme 59:**
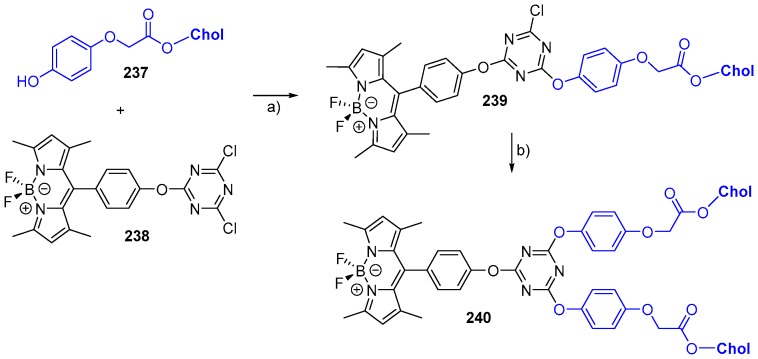
Synthesis of cholesterol-triazine-BODIPY trimers. Reagents and conditions: **a**) Na_2_CO_3_, acetone, rt, 6 h; **b**) 237, Na_2_CO_3_, THF, reflux, 10 h. BODIPY: Boron dipyrromethene.

The synthesis of the cholesterol-triazine-BODIPY trimers **239** and **240** with one or two cholesterol units involved the reaction of cyanuric chloride-substituted BODIPY derivative **238** with an esterified cholesterol derivative (**237**), using different reaction conditions (Scheme 59) [61].

The cholesterol-triazine-BODIPY trimers **239** and **240** exhibited distinct mesomorphic properties, dependent on the number of cholesterol units. The one-cholesterol unit derivative **239** showed nematic liquid crystal behavior, while the two-cholesterol unit **240** was a hexagonal columnar liquid crystal. The photophysical properties of both compounds were also addressed, and the authors concluded that both derivatives presented good fluorescence intensities with higher quantum yields and larger Stokes shifts when compared to their precursors. The authors claimed that this study reported the first examples of cholesterol-BODIPY liquid crystals, in which the introduction of a cholesterol unit was favorable for both liquid crystalline behavior and improved fluorescence [61].

**Scheme 60:**
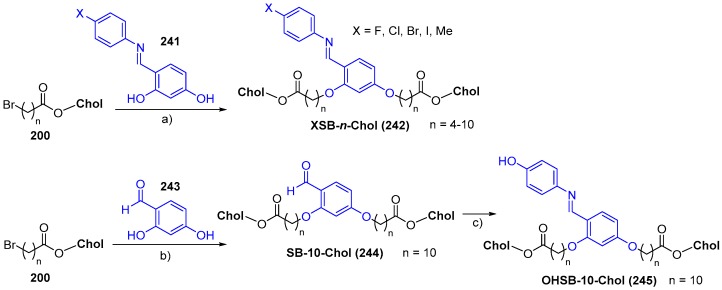
Synthesis of λ-shaped chiral liquid crystal trimers. Reagents and conditions: **a**) K_2_CO_3_, KI, DMF, 90 °C, 24 h; **b**) K_2_CO_3_, acetone, reflux, 8 h; **c**) 4-aminophenol, EtOH, AcOH, reflux, 5 h.

The synthesis of two series of λ-shaped dicholesteryl-based conjugates, **242** and **245**, containing a Schiff base core linking two cholesteryl ester units was reported. The first series of compounds was prepared based on a Williamson etherification between the Schiff-base (**241**) and cholesteryl bromo-alkanoates (**200**) to afford **XSB-*n*-Chol** (*n* = 4–10) derivatives (**242**) (Scheme 60) [62]. The synthesis of **SB-10-Chol** (**244**) was slightly different and involved the alkylation of 2,4-dihydroxybenzadehyde (**243**) by cholesteryl bromo-decanoate (**200**) followed by condensation with 4-aminophenol to afford **OHSB-10-Chol** (**245**) (Scheme 60) [62]. The study of the liquid crystal properties of the conjugates **242** and **245** indicated that the compounds had enantiotropic chiral nematic behavior, with an exception for short conjugates, which formed an additional SmA phase along with the narrow intermediary TGB phase. All compounds showed mesogenic properties, as they could form oily streaks, fan-shaped filaments, and Grandjean textures in the liquid crystalline state. The authors also found that long spacer compounds vitrified to form stable cholesteric glassy states instead of crystallization. Furthermore, the mesomorphic temperature range increased alongside the length of the spacer (from *n* = 4 to *n* = 10), showing an odd-even alternation on the clearing and transition temperatures [62].

**Scheme 61:**
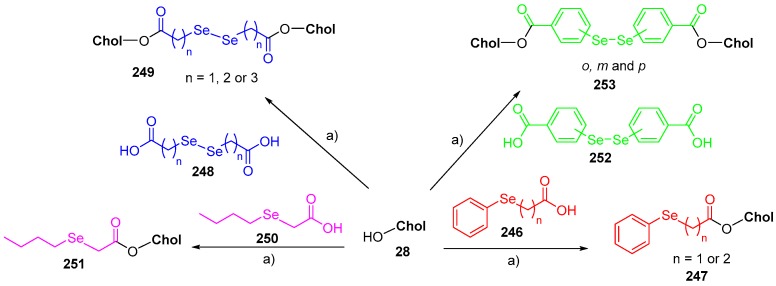
Synthesis of cholesterol-based selenides and diselenides. Reagents and conditions: (**a**) DMAP, DCC, CH_2_Cl_2_, rt, overnight.

In 2015, Frizon and coworkers described the synthesis of and preliminary studies on the thermal and photophysical properties of selenium liquid crystals containing cholesterols **247**, **249**, **251**, and **253**. The synthesis of three new series of selenide **247**/**251** and diselenide compounds **249**/**253** was accomplished via esterification of cholesterol **28** with the appropriate selenide **246**/**250** or diselenide acid **248**/**252** (Scheme 61) [63].

All synthesized compounds presented good thermal stability. Six of them showed liquid crystal properties, in which selenide **251** and alkyl diselenides **249** (*n* = 2) and **249** (*n* = 3) exhibited an SmC* mesophase, whereas aryl diselenide **253**, with higher structural rigidity, showed a chiral enantiotropic smectic A (SmA*) mesophase. Furthermore, all these new selenide-cholesterol compounds showed higher glutathione peroxidase-like activity than the standard ebselen, with selenide **249** (*n* = 2) the most active [63].

**Scheme 62:**
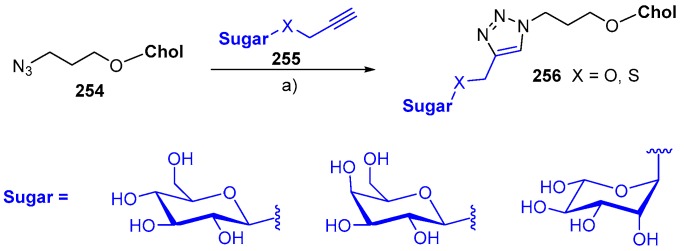
Synthesis of *O*- and *S*-glyco-triazole-cholesterol derivatives. Reagents and conditions: **a**) CuSO_4_·5H_2_O, AscONa, 1,4-dioxane/H_2_O (4:1), 80 °C, 24 h.

A series of glycosteroids (**256**) constituted by cholesterol and distinct glycosidic moieties were synthesized by coupling propargyl 1-*S*-propargyl d-glucose, d-galactose, or l-rhamnose (**255**) to cholesterol scaffold **254** through a CuAAC reaction (Scheme 62) [64]. This study aimed to analyze if the sugar structure as well as the heteroatom linked to the anomeric position had an impact on the liquid-crystalline properties of the glycosteroids (**256**). The mesomorphic temperature range found for the glycosteroids (**256**) was higher than that generally reported in the literature, but similar to that reported for other glycosteroids. All the studied glycosteroids (**256**) showed great phase stability compared to those already studied, and interestingly, glycosteroids (**256**) (sugar = d-glucose; X = S) showed no decomposition even at 200 °C. These results offer new possibilities in the development of new high-temperature captors or detectors [64].

## 5. Cholesterol-Based Gelators

Low molecular weight organic gelators (LMOGs) are small organic molecules that self-assemble in water or organic solvents, forming a 3D network that entraps the liquid phase, resulting in gel formation. In recent years, these classes of compounds have attracted much attention because of their range of applications, for example as alternative biomaterials for drug delivery or tissue engineering [65,66]. New generations of steroidal low molecular mass gelators (LMGs) are usually designed through the assembly of various building units such as a steroid derivative (S), a linker unit (L), and often an aromatic platform (A) around which the steroid units can be positioned through linkers. The good gelation ability of the steroidal LMGs led to the development of a series of steroid-based gelators commonly classified as ALS, arranged in A(LS)_2_, A(LS)_3_, LS, or LS_2_ molecular types [65].

**Scheme 63:**
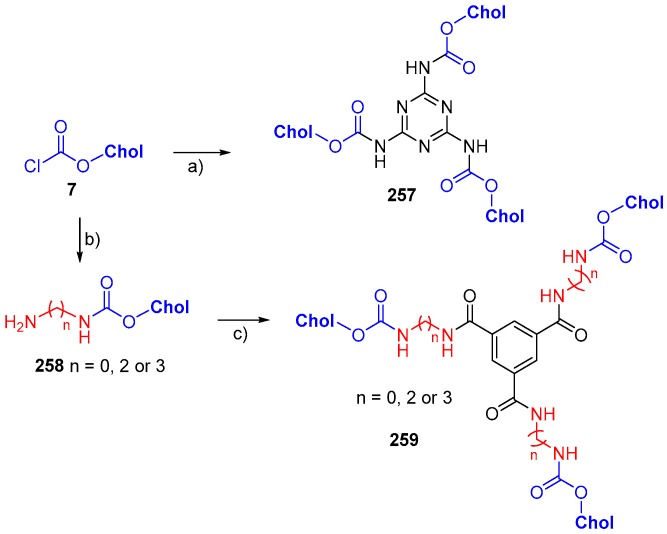
Synthesis of triangular A(LS)_3_-type cholesteryl derivatives with different aromatic platforms and linkers. Reagents and conditions: **a**) melamine, Et_3_N, MeCN, reflux, 9 days; **b**) hydrazine hydrate (*n* = 0) or ethylenediamine (*n* = 2) or 1,3-diaminopropane (*n* = 3), Et_3_N, CH_2_Cl_2_, rt, overnight; **c**) benzene-1,3,5-tricarbonyl chloride, Et_3_N, THF, rt, overnight.

In 2014, an interesting study was reported involving the design of an uncommon class of cholesteryl-based triangular A(LS)_3_-type low molecular mass gelators and the exploration of their gelation and anion-sensing applications. The design strategy was based on placing three cholesteryl derivatives using linker units around melamine or benzene-1,3,5-tricarbonyl chloride as aromatic platform precursors. The synthesis of compounds **257** and **259** involved the reaction of cholesteryl chloroformate **7** with different amines in one- or two-step procedures (Scheme 63) [67].

This study also involved the evaluation of gelation and self-assembly properties of this new class of compounds by comparing them to the existing cholesteryl-based LMGs. The results indicated that the gelation and self-assembly properties of compounds **257** and **259** could be controlled by modification of the structural features of the A(LS)_3_-type molecule. Increasing the length of the linker units, the fibrous xerogel networks assembled into more porous fiber networks. Moreover, the authors found that the compounds **257** and **259** could be used as selective sensors for F^−^, and their selectivity could be enhanced by increasing the chain length of their linker units [67].

**Scheme 64:**
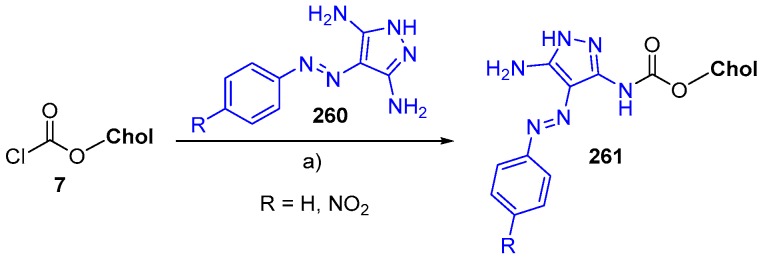
Synthesis of cholesterol-azobenzyl organogels. Reagents and conditions: **a**) Et_3_N, CHCl_3_, rt, 24 h.

Two new cholesterol-based compounds (**261**) were also reported as fluoride-responsive organogels. Their design was based on the coupling of compounds in **260**, bearing azo units as the chromophore and a pyrazole group as the anion acceptor, with the cholesteryl chloroformate **7** (Scheme 64) [68].

The authors observed that structural modifications on the benzyl core of compound **261** (R = H or NO_2_), hydrogen bonding, hydrophobic interactions, as well as π-π stacking interactions, had considerable influence on the gel-sol transition properties. Moreover, they also found that the gel was selectively fluoride-responsive among the tested anions, expressing gel-sol transition and red-purple color changes easily detected by the naked eye [68]. 

**Scheme 65:**
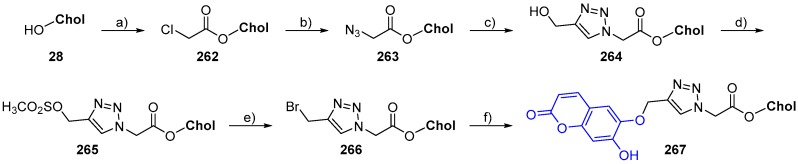
Synthesis of triazole-linked cholesterol-coumarin. Reagents and conditions: **a**) chloroacetyl chloride, pyridine, dry CH_2_Cl_2_, rt, 10 h; **b**) MeCN, NaN_3_, reflux, 5 h; **c**) propargyl alcohol, CuSO_4_, Cu turning, EtOH, reflux, 90 °C, 6 h; **d**) methanesulfonyl chloride, CH_2_Cl_2_, Et_3_N, rt, 30 min; **e**) LiBr, THF, rt, 8 h; **f**) 6,7-dihydroxycoumarin, MeCN, Cs_2_CO_3_, reflux, 36 h.

Following the purposes of the selective detection of F^−^, a new coumarin-based supramolecular gelator (**267**) was designed [69]. The reported compound **267** follows a simple architecture that bears a coumarin-appended 1,2,3-triazole coupled with cholesterol, synthesized in a six-step route as depicted in Scheme 65. The coumarin moiety acts as a fluorescence signaling unit, the 1,2,3-triazole as a linker and as an anion binding site, and cholesterol as a hydrophobic surface.

The authors concluded that cooperative hydrogen bonding between phenolic OH and a 1,2,3-triazole ring as well as hydrophobic-hydrophobic interactions of the cholesteryl groups in compound **267** played a crucial role in the formation of an organogel. Furthermore, it was demonstrated that compound **267** organogel was sensitive for F^−^ and HP_2_O_7_^3−^ detection by means of gel phase transformation as well as fluorimetrically, showing considerable changes in emission properties [69].

**Scheme 66:**
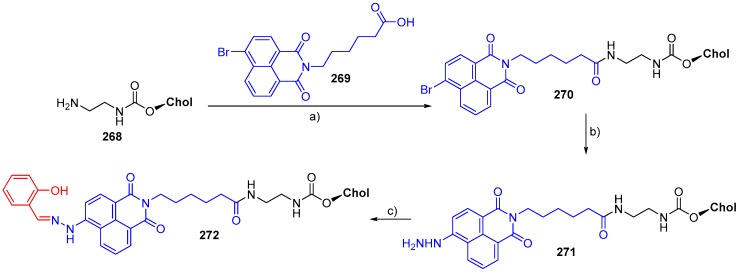
Synthesis of cholesterol naphthalimide-based gelators. Reagents and conditions: **a**) EDAC/BtOH; b) NH_2_NH_2_·H_2_O, CuCl, EtOH, reflux, 3 days;**c**) salicylaldehyde, EtOH, reflux, 24 h.

A novel cholesterol-based organogelator containing D-A (donor-acceptor) pairs (salicylaldehyde and naphthalimide units) (**272**) was synthesized [70]. The synthetic strategy relied on the introduction of the electron-rich salicylaldehyde group into a naphthalimide-based organogelator through a Schiff-base reaction (Scheme 66). This cholesterol-based organogelator (**272**) was found to form stable and chiral gels with different optical properties and morphologies in several organic solvents. An interesting feature of compound **272** was the changing of the color and emission color of the organogel in benzene, which varied from yellow-green to red during the thermoreversible sol-gel transformation, demonstrating for the first time solvent-controlled multiple color emission achieved in a monocomponent gel system. This feature makes the organogel **272** quite suitable for applications in optical switches, sensors, and smart materials [70].

**Scheme 67:**
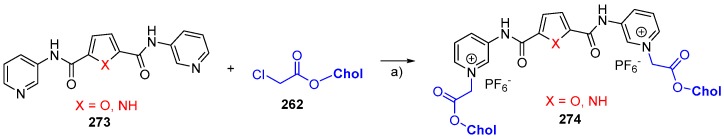
Synthesis of cholesteryl-bisamides. Reagents and conditions: **a**) 1. DMF, MeCN, reflux, 3 days; 2. NH_4_PF_6_, DMF/MeOH (1:10, *v*/*v*), H_2_O, hot, 30 min.

To develop new supramolecular gelators, Panja and coworkers synthesized pyrrole and furan-based pyridine/pyridinium bisamides containing cholesteryl units in their architecture [71]. The synthesis of cholesterol-based bisamides (**274**) was achieved through the coupling reaction of cholesteryl chloroacetate derivate **262** with the pyridine ring nitrogens in bisamide **273** (Scheme 67).

The gelation properties of both bisamide **273** and bisamides with a cholesteryl unit attached (**274**) were evaluated. In aqueous DMSO, compound **274** (X = O) exhibited nongelation properties, while compound **274** (X = NH) produced a light yellow colored gel. This suggests that the heteroatom of the aromatic linker played a crucial role in gelation. The organogel formed by compound **274** (X = NH) revealed itself to be a good anion sensor, since the gel state was selectively ruptured into solution in the presence of F^−^ and AcO^−^ anions. Interestingly, the gel rupture induced by F^−^ was recovered upon the addition of Fe^3+^. This feature is very useful in the visual distinction of F^−^ from AcO^−^ anions [71].

**Scheme 68:**
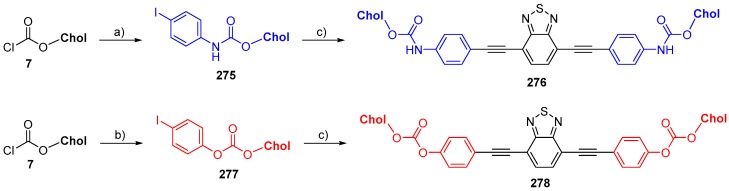
Synthesis of cholesterol-benzothiadiazole based compounds. Reagents and conditions: **a**) 4-iodoaniline, Et_3_N, CH_2_Cl_2_, rt, 12 h; **b**) 4-iodophenol, K_2_CO_3_, acetone, reflux, 24 h; **c**) 4,7-diethynyl-2,1,3-benzothiadiazole, CuI, Pd(PPh_3_)_4_, di-isopropylamine, THF, reflux, 24 h.

A different kind of fluorescent organogelator based on cholesterol containing benzothiadiazole fluorophores **276** and **278** was designed and synthesized by Sun and coworkers (Scheme 68). The authors aimed to understand the role of hydrogen bonding and π–π interactions and to study the changes of fluorescent properties in the process of gelation of cholesterol-based π-conjugated organogels [72].

The authors studied three methods of gel preparation (heating-cooling process, ultrasonic treatment, and mixed solvents, at room temperature) and found that π–π and H-bonding interactions should be the key contributors in forming gels of **276**, while in gel formations of **278**, only π–π interactions seemed to matter. The obtained results suggest that these two multiple-stimuli responsive luminescent gels, **276** and **278**, can be used as smart soft materials sensitive to temperature, solvent, ultrasound, and Hg^2+^ [72].

**Scheme 69:**
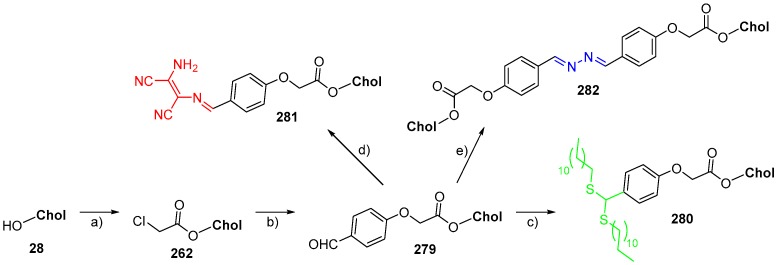
Synthesis of cholesterol-based dithioacetal. Reagents and conditions: **a**) chloroacetyl chloride, pyridine, dry CHCl_3_, rt, 10 h; **b**) 4-hydroxybenzaldehyde, K_2_CO_3_, MeCN, 5 h; **c**) 1-dodecanethiol, BF_3_·OEt_2_, dry CH_2_Cl_2_, 0 °C, 30 min; **d**) diaminomalenonitrile, dry benzene, reflux, 3 days; **e**) NH_2_NH_2_·H_2_O, dry benzene, reflux, 3 days.

Recently, Panja and Ghosh reported three related works involving cholesterol conjugates bearing three different moieties (dithioacetal **280**, diaminomalononitrile **281**, and diazine **282** functional groups) for sensing a series of cations such as Hg^2+^, Cu^2+^, Ag^2+^, and Fe^2+^ [73,74,75]. The three cholesterol conjugates were synthesized using the same three-step methodology, except for the final step, which involved the reaction of the intermediate benzaldehyde **279** with 1-dodecanethiol, diaminomalononitrile, and hydrazine to afford cholesterol conjugates **280**, **281**, and **282**, respectively (Scheme 69). The cholesterol-dithioacetal conjugate **280** was used for the detection of Hg^2+^ and incorporated two distinct components: i) A cholesterol motif to assist the self-assembly of the molecules through hydrophobic interaction; and ii) a thiol part that was used as the reaction-based recognition unit of the molecule [73].

The authors studied the sensing mechanism for Hg^2+^ of the cholesterol-dithioacetal conjugate, realizing that the specific Hg^2+^-induced deprotection of the thioacetal functionality of **280** resulted in sol-to-gel transition in DMF/H_2_O (1:1, *v*/*v*) through the formation of precursor aldehyde **279**. The authors also claimed that this was the first chemodosimeter that functions as a selective “naked-eye” Hg^2+^-detector by showing in situ sol-to-gel conversion [73].

The cholesterol-diaminomalononitrile conjugate **281** was found to form supramolecular gels in dimethylformamide (DMF)/H_2_O and 1,2-dichlorobenzene, as confirmed by rheological studies. In addition, the authors verified that the gel formed in DMF/H_2_O was more stable and robust than the one obtained from 1,2-dichlorobenzene, due to strong intermolecular forces among the gelators in DMF/H_2_O. Furthermore, it was also established that cholesterol-diaminomalononitrile **281** gel was selective for visual recognition of Hg^2+^ and Cu^2+^ ions, and for sensing hydrazine based on the dosimetric interaction of the malononitrile motif with hydrazine [74].

Concerning the cholesterol-diazine conjugate **282**, the authors demonstrated that it could form nice gels with Ag^+^ and Fe^3+^ ions in a CHCl_3_/CH_3_OH mixture solvent, using the diazine moiety as a metal ion binding site. The gelator **282** was able to distinguish Ag^+^ and Fe^3+^ with the aid of tetrabutylammonium chloride, tetrabutylammonium bromide or fluoride, and ammonium thiocyanate. Furthermore, the authors proved that there was no interference of Fe^2+^ ions in the detection of Fe^3+^ ions, as in the case of most chemosensors and gelators [75].

**Scheme 70:**
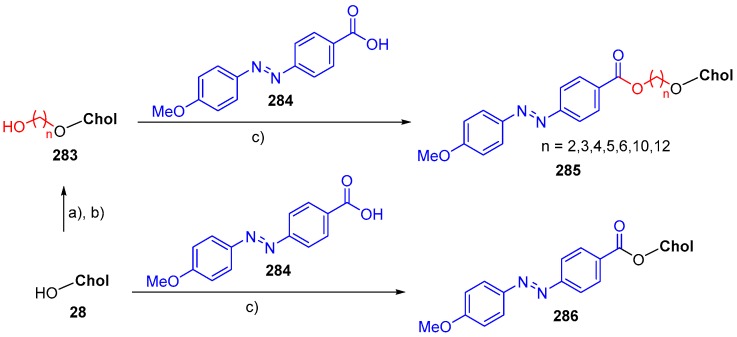
Synthesis of azobenzene-cholesterol compounds with different spacers. Reagents and conditions: **a**) TsCl, DMAP, Et_3_N, CH_2_Cl_2_, 48 °C, 12 h; **b**) HO(CH_2_)_n_OH, 1,4-dioxane, reflux, 4 h; **c**) DCC, DMAP, CH_2_Cl_2_, 24 h.

The effect of different spacer lengths containing two, three, five, six, ten, or twelve carbon atoms on cholesterol-based azobenzene organogels **285** and **286** was investigated [76]. For this purpose, a series of seven azobenzene-cholesterol compounds was synthesized through esterification reactions of cholesterol derivatives of **283** (bearing different spacer lengths) with 4′-carboxy-4-methoxyazobenzene **284** carried out in the presence of *N*,*N*′-dicyclohexylcarbodiimide (DCC) and dimethylaminopyridine (DMAP) in dichloromethane, as depicted in Scheme 70.

Typical reversible *trans*-*cis* and *cis*-*trans* isomerization of the azobenzene units was observed upon UV-Vis irradiation, giving the compounds **285** and **286** recoverable photoresponsive properties. Differential scanning calorimetry studies revealed that the spacer length plays a crucial role in the gelation phenomenon. Interestingly, among the tested compounds, only **285** (*n* = 6) could form a gel, and in specific solvents such as ethanol, isopropanol, and butan-1-ol. Furthermore, the authors concluded that the solvents, intermolecular H-bonding, and van der Waals interactions affected the aggregation mode and morphology of the gels [76].

**Scheme 71:**
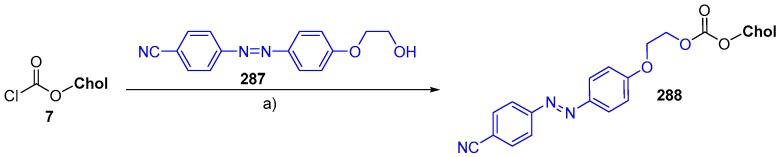
Synthesis of azobenzene-cholesterol-based dimesogen. Reagents and conditions: **a**) pyridine, toluene, 100 °C, 9 h.

In 2016, a study was reported on liquid crystal (LC) and gelation-based self-assembly, as well as the photoresponsive behavior of a new unsymmetrical azobenzene-cholesterol based dimesogen, **288** [77]. This molecule assembles a CN group at one end and a cholesterol carbonate, fixed through an oxyethylene spacer, to the opposite end of the azobenzene unit (Scheme 71).

Compound **288** presented the capacity of acting as a chiral mesogenic dye dopant to induce a high helical-twisting chiral phase in the common nematic phase of 5CB. In addition, the gels of **288** formed in organic solvents exhibited multiple stimuli-responsive behaviors upon exposure to environmental stimuli such as temperature, light, and shear forces. The photoresponsive character was also proven in solution, in LC and gel states. These properties give to compound **288** potential applications in displays, as chiral mesogenic dye dopants, photochemical molecular switches, and new versatile LMGs [77].

**Scheme 72:**
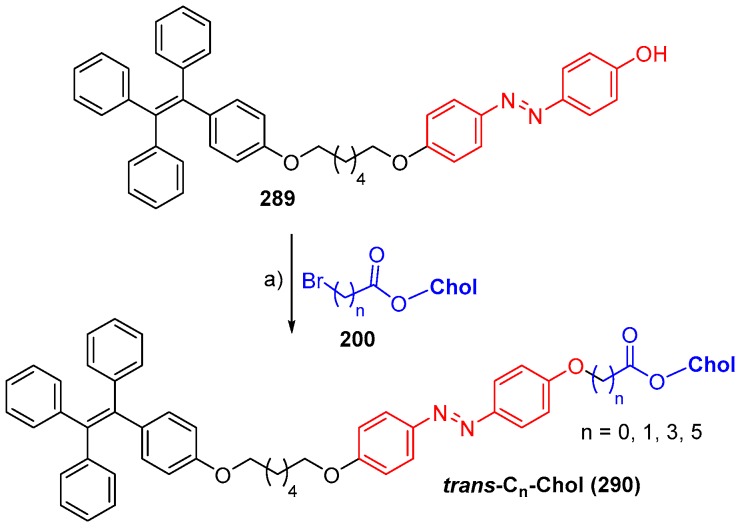
Synthesis of ***trans*-C_n_-Chol**. Reagents and conditions: **a**) K_2_CO_3_, DMF, 50 °C, 24 h.

A new series of liquid crystal gelators (**290**) with photoresponsive and aggregation-induced emission (AIE) properties was synthesized by connecting cholesterol derivatives **200** and tetraphenylethylene (an important AIEgen) to a central azobenzene moiety through esterification reaction (Scheme 72) [78]. The authors included variations in the alkyl chain spacer (*n* = 0, 1, 3, 5) to adjust the distance between cholesterol and azobenzene, while a fixed alkyl chain was placed between azobenzene and tetraphenylethylene (Scheme 72). The liquid crystal properties of compounds in **290** were assessed, and the results showed that all compounds exhibited, in pure state, smectic A LC phases, enantiotropic for **290** (*n* = 0) and (*n* = 3), but monotropic for **290** (*n* = 1) and (*n* = 5). The gelation properties of compound **290** demonstrated that **290** (*n* = 3) and (*n* = 5) form stable gels in appropriate solvents or solvent mixtures, while **290** (*n* = 0) and (*n* = 1) cannot form gels in a range of solvents. An interesting feature of both **290** (*n* = 3) and (*n* = 5) LMOGs is that they have significantly enhanced emissions induced by molecular self-assembly into fibril or ribbon-like nanostructures [78].

**Scheme 73:**
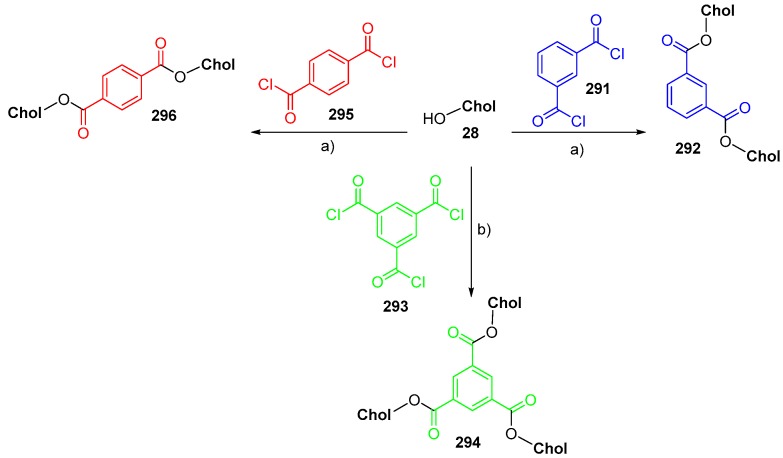
Synthesis of cholesterol-based A(LS)_n_-type gelators. Reagents and conditions: **a**) DMAP, Et_3_N, toluene, 90 °C, 2 days; **b**) DMAP, Et_3_N, toluene, 90 °C, 5 days.

Three new cholesteryl-based A(LS)_2_- and A(LS)_3_-type LMGs, **292**, **294**, and **296**, without hydrogen bond linkers, were reported in the literature, synthesized through esterification reactions of acid chlorides **291**, **293**, and **295** with cholesterol **28** in the presence of DMAP (Scheme 73) [79]. The study of the gelation properties in various organic solvents indicated that the number and position of the substituents in the cholesteryl moieties attached to a benzene ring had a great influence on the gelation as well as in the aggregation behaviors of the A(LS)_2_- and A(LS)_3_-type LMOGs. Among these three gelators, **294** and **296** showed efficient gelation abilities even without hydrogen bond linkers, in contrast with the meta-substituted **292**, which did not gelate in any tested solvent [79].

**Scheme 74:**
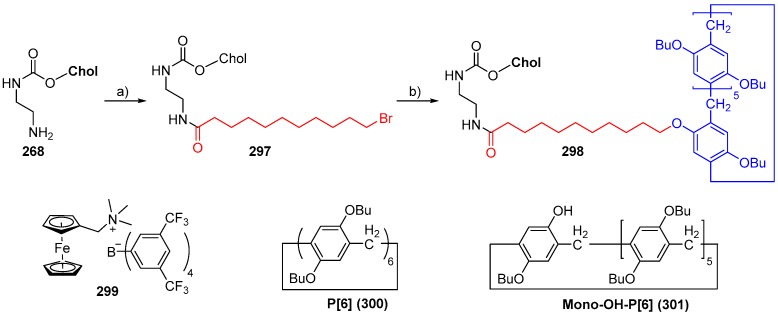
Synthesis of pillar[6]arene-functionalized cholesterol. Reagents and conditions: **a**) 11-bromoundecanoyl chloride, DIPEA, CHCl_3_, rt, 12 h; **b**) **Mono-OH-P[6]**, Cs_2_CO_3_, DMF, rt, 24 h.

Recently, the synthesis of a new pillar[6]arene-functionalized cholesterol derivative (**298**), acting as an LMG, was reported in the literature [80]. In this new compound, the host–guest pillar[6]arene **300** was linked to a cholesterol unit by the long alkyl chain, as well as amide groups (Scheme 74). This new pillar[6]arene-cholesterol **298** was found to form an organogel in cyclohexane/hexan-1-ol (10:1, *v*/*v*), which was reversibly responsive to temperature, share stress, and partially host–guest interaction introduced by ferrocenyl iminium derivative **299**. In the case of the addition of ferrocenyl iminium derivative **299**, the organogel could be tuned into a solution and tuned back into the organogel upon addition of per-butylated pillar[6]arene **300**. This interesting feature could be explained on the basis of host–guest interactions of individual **300** with cationic guest **299** that bound with pillar[6]arene-cholesterol gelator **298** [80].

**Figure 5:**
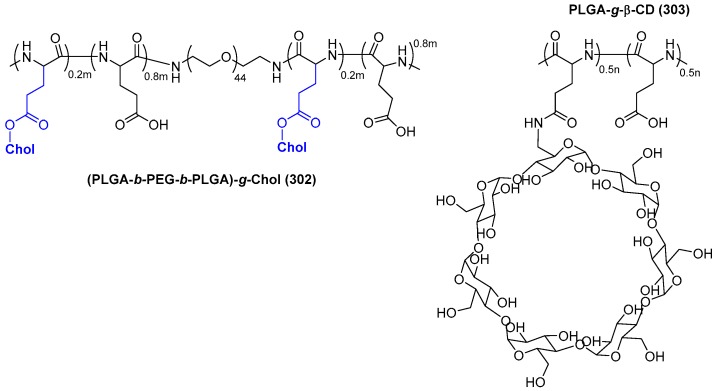
Chemical structures of (**PLGA-*b*-PEG-*b*-PLGA)-g-Chol** and **PLGA-*g*-β-CD**.

In 2015, the development of a new kind of self-healing, degradable, and biocompatible polypeptide hydrogel based on self-assembly between cholesterol-modified triblock poly(l-glutamic acid)-*block*-PEG-*block*-poly(l-glutamic acid) [(**PLGA-*b*-PEG-*b*-PLGA)-*g*-Chol**] **302** and β-cyclodextrin (β-CD)-modified poly(l-glutamic acid) (**PLGA-*g*-β-CD**) **303** (Figure 5) was reported in the literature [81].

The authors observed that the hydrogel formation was based on the host and guest linkage between β-cyclodextrin (β-CD) and cholesterol, and that their viscoelastic behavior depended on polymer concentration as well as the β-CD/Chol molar ratio. Those hydrogels showed very interesting self-healing capabilities, good cytocompatibility, excellent flexibility, and quick colorant diffusion. With all these features, it is anticipated that these self-healable hydrogels may have important applications in tissue engineering [81].

## 6. Bioimaging Applications

Imaging techniques, particularly fluorescence imaging techniques, have become powerful tools for noninvasive visualization of biological processes in real time with high spatial resolution. Methods to “see into the body” or “see into cells” are essential for the diagnosis and treatment of a disease, as well as for research into the basic processes of life. Therefore, bioimaging techniques to visualize physiological or pathophysiological changes in the body and cells have become increasingly important in biomedical sciences [82].

**Scheme 75:**
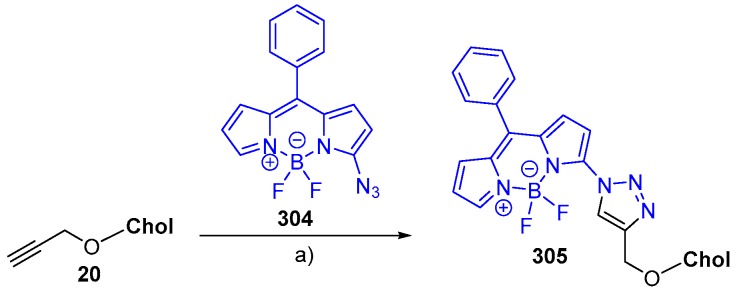
Synthesis of BODIPY-triazole-cholesterol fluorescent dye. Reagents and conditions: **a**) CuSO_4_, AscONa, CH_2_Cl_2_/H_2_O (2:1), rt.

The synthesis of a series of BODIPY-based fluorogenic dyes was reported, involving the CuAAC reaction of a nonfluorescent BODIPY-azide, **304**, with a series of nonfluorescent alkyne molecules, including *O*-propargylated cholesterol **20** (Scheme 75) [83]. The most interesting molecule was the cholesterol-linked dye **305**, which presented red-shifted absorption and emission wavelengths and displayed its preferential accumulation at the intracellular membranes over the plasma membrane of HeLa cells. This result offers potential applications of cholesterol-BODIPY conjugate **305** in the bioimaging of cholesterol trafficking in living cells and organisms [83].

**Scheme 76:**
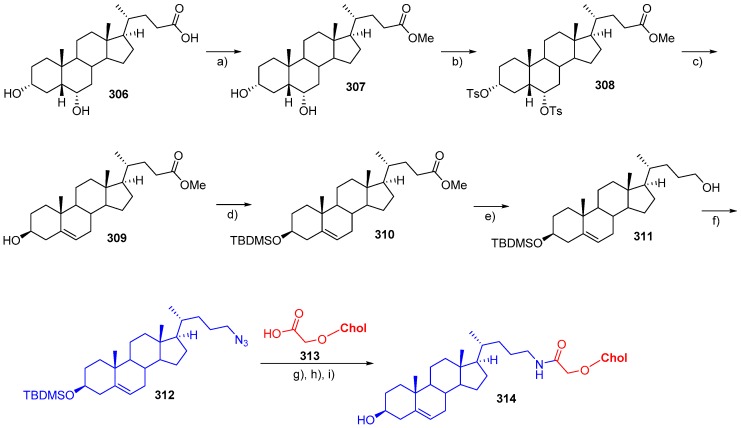
Synthesis of cholesterol-based crosslinker. Reagents and conditions: **a**) *p*-TsOH, MeOH, 25 °C, 24 h; **b**) TsCl, pyridine, 25 °C, 4 h; **c**) KOAc, DMF/H_2_O, reflux, 12 h; **d**) TBDMSCl, imidazole, pyridine/DMF, 25 °C, 1.5 h; **e**) LiAlH_4_, THF, 0 °C, 1.5 h; **f**) (PhO)_2_P(O)N_3_, DPPA, PPh_3_, DIAD, THF, 0 to 25 °C, 22 h; **g**) Ph_3_P, H_2_O, THF, reflux, 3 h; **h**) DCC, DMAP, CH_2_Cl_2_, 0 to 25 °C, 1.5 h; **i**) TBAF, THF, 25 °C, 24 h.

Byrd and coworkers reported the synthesis of a crosslinker containing two independent cholesterol units, with or without a photoaffinity label, guided by computational methods based on a model for the transfer of a cholesterol molecule between two proteins, NPC1 and NPC2 [84]. The synthesis of crosslinker **314** (without a photoaffinity label) involved several steps, especially because of the demanding six-step synthetic route of one of the portions that constitutes the crosslinker **314** (Scheme 76) [84].

**Scheme 77:**
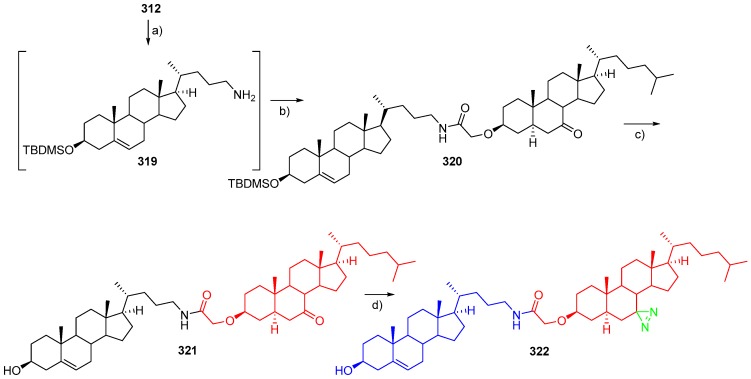
Synthesis of cholesterol-based crosslinker with photoaffinity label. Reagents and conditions: **a**) PPh_3_, THF/H_2_O, reflux, 3 h; **b**) 318, DCC, DMAP, CH_2_Cl_2_, 25 °C, 1.5 h; **c**) HF.pyridine, THF, 25 °C, 48 h; **d**) NH_3_, H_2_NOSO_3_H, I_2_, MeOH/THF, 0 to 25 °C, 15 h.

**Scheme 78:**
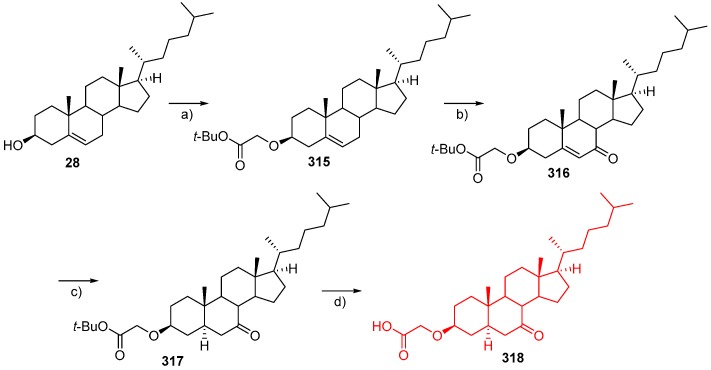
Synthesis of carboxylic acid cholesterol derivative. Reagents and conditions: **a**) *t*-BuOK, *t*-butyl bromoacetate, toluene, 25 °C, 17 h; **b**) 70% aq *t*-BuOOH, PDC, celite, benzene, 25 °C, 24 h; **c**) H_2_ (1 atm), 10% Pd/C, MeOH/CH_2_Cl_2_, 25 °C, 14 h; **d**) HCO_2_H, Et_2_O, 65 °C, 4 h.

Another cholesterol-based crosslinker (**322**) with a photoaffinity label was also synthesized (Scheme 77) [84]. The synthesis of such a compound involved two stages: i) The preparation of an appropriate carboxylic acid cholesterol moiety (**318**) (Scheme 78) [84]; and ii) the linkage between compounds **318** and **312** (previously synthesized) (Scheme 76) [84].

The authors claimed that with the appropriate connection of the two cholesterol molecules **314** and **322**, both proteins (NPC1 and NPC2) are simultaneously occupied in a manner that stabilizes the protein–protein interaction, allowing detailed structural analysis of the resulting complex. Furthermore, the introduction of a photoaffinity label in one of the cholesterol moieties, **322**, should allow the covalent attachment of one of the units into its respective protein-binding pocket. The compounds synthesized in this work may be interesting tools for studying the transfer of cholesterol between cholesterol-binding proteins [84].

**Scheme 79:**
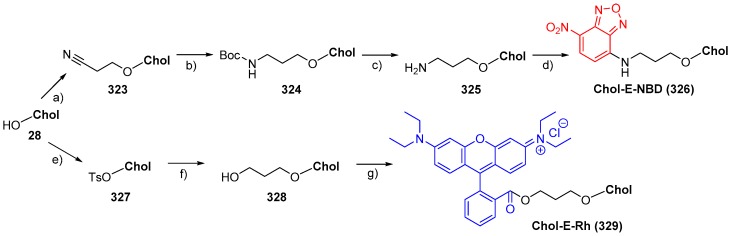
Synthesis of fluorescent lipids. Reagents and conditions: **a**) acrylonitrile, 18-crown-6, aq KOH/CH_2_Cl_2_; **b**) NiCl_2_·6H_2_O, Boc_2_O, NaBH_4_/MeOH; **c**) TFA/CH_2_Cl_2_; **d**) NBD-Cl, CHCl_3_, rt, overnight; **e**) TsCl, pyridine, CHCl_3_; **f**) propane-1,3-diol, anhydrous 1,4-dioxane; **g**) rhodamine B, DCC, DMAP, CH_2_Cl_2_, rt, 2 h.

Two cholesterol-based fluorescent lipids, **326** and **329**, were synthesized using nitrobenzoxadiazole (NBD) or rhodamine B, respectively, linked by an ether alkyl chain (Scheme 79). Compounds **326** and **329** were incorporated into liposome formulations, aiming to create and validate their use as fluorescent probes for lipoplex tracking, without interfering with green fluorescent protein (GFP) [85]. The authors concluded that both compounds **326** and **329** did not interfere with the expression of GFP plasmid, obtaining live cell images without any interference. Furthermore, microscopic observations clearly showed that these fluorescent lipids had minimal self-quenching and photobleaching effects. The results indicated that the synthesized compounds **326** and **329** may be considered for the development of fluorescent probes to trace the intracellular trafficking of cholesterol-derived cationic liposomes [85].

**Scheme 80:**
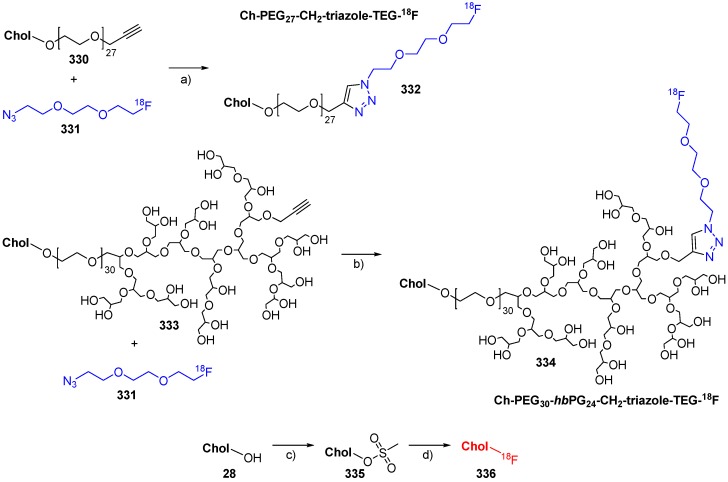
Synthesis of radioactive polymers **Ch-PEG_27_-CH_2_-triazole-TEG-^18^F**, **Ch-PEG_30_-*hb*PG_24_-CH_2_-triazole-TEG-^18^F**, and 3-[^18^F]Fluoro-cholest-5-ene. Reagents and conditions: **a**) CuSO_4_, AscONa, PBS, DMSO, 70 °C, 15 min; **b**) CuSO_4_, AscONa, PBS, EtOH, 70 °C, 15 min; **c**) methanesulfonyl chloride, Et_3_N, CH_2_Cl_2_, rt, 16 h; **d**) tetrabutylammonium hydroxide (TBAH), [^18^F]Fluoride, MeCN, 120 °C, 20 min.

Reibel et al. prepared radiolabeled-^18^F polymer compounds based on linear PEG **332** and novel linear-hyperbranched amphiphilic polyglycerol (*hb*PG) **334**, using cholesterol **28** as a lipid anchor via CuAAC chemistry of propargylated compounds **330** and **333** with radiolabeled-^18^F azide **331** (Scheme 80) [86].

The authors also carried out direct labeling of cholesterol **28** with ^18^F (Scheme 80) and performed in vivo positron emission tomography (PET) studies as well as ex vivo biodistribution studies in mice with both polymers (**Ch-PEG_27_-CH_2_-triazole-TEG-^18^F 332** and **Ch-PEG_30_-*hb*PG_24_-CH_2_-triazole-TEG-^18^F 334**) and ^18^F-cholesteryl fluoride **336**. These three new derivatives were incorporated into liposome formulations. The results showed that both polymers **332** and **334** were quickly excreted by renal function, whereas ^18^F-cholesteryl fluoride **336** showed some retention in the lung, liver, and spleen. Liposome formulations with the new polymers showed different physical properties from those of the conventional liposomes with ^18^F-cholesteryl fluoride **336**, as well as fast uptake by the liver, spleen, and lung. Furthermore, the novel *hb*PG-polymer liposomes of **334** showed similar behavior to the PEG-shielded vesicles, enhancing multifunctionality without the loss of pharmacokinetic properties. This approach opens new possibilities in the field of polymer tracking in vivo and liposome tracing in mice via PET [86].

**Scheme 81:**
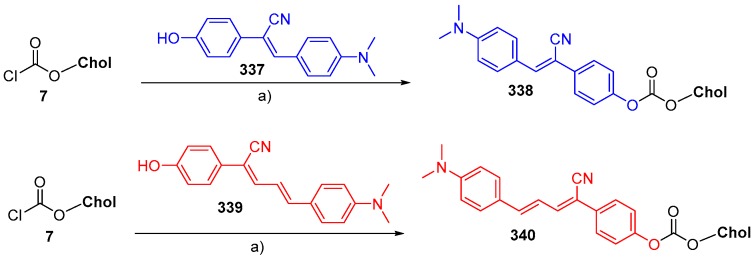
Synthesis of cholesterol-tethered aggregation-induced enhanced emission (AIEE) fluorogens. Reagents and conditions: **a**) pyridine, benzene, reflux, 12 h.

In 2015, Palakollu and Kanvah designed and synthesized cholesterol-conjugated chromophores of α-cyanostilbene/diene **338** and **340** exhibiting intramolecular charge transfer (ICT) and aggregation-induced enhanced emission (AIEE). Compounds **338** and **340** were easily prepared from the reaction of cholesterol chloroformate **7** with either a stilbene **337** or diene derivative **339** (Scheme 81) [87].

The authors carefully studied the absorption and emission properties of both cholesterol conjugates **338** and **340** and their parent chromophores **337** and **339**. An ICT behavior was observed for diene compounds **339** and **340**, whereas for stilbene compounds **337** and **338** a remarkable AIEE behavior was detected. The lack of AIEE characteristics in dienes may be explained by the competing nonradiative losses due to double bond flexibility. Nevertheless, the most interesting conclusion of the optical properties study was that the random aggregates formed by stilbene **337** in aqueous media became highly ordered upon cholesterol conjugation **338**. Furthermore, the interaction with sodium cholate stimulated the formation of self-assembled structures in nanoscale dimensions, making these conjugates the starting point for the development of several bioimaging probes [87].

**Scheme 82:**
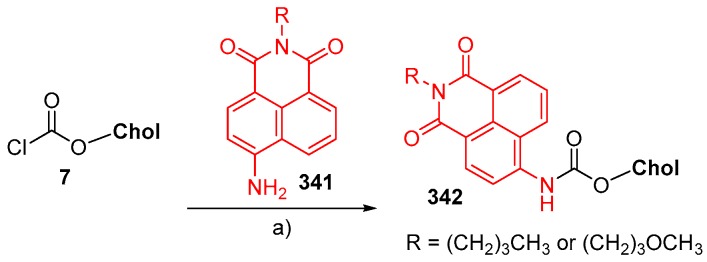
Synthesis of cholesterol-naphthalimide conjugates. Reagents and conditions: **a**) DIPEA, EtOAc, 55 °C, 5 days.

In 2016, Wercholuk and coworkers synthesized a fluorescent-labeled cholesterol molecule (**342**) by treating cholesteryl chloroformate **7** with 4-amino-1,8-naphthalimides (**341**) (Scheme 82) [88]. The authors expected that such conjugates might serve one of two roles, depending on whether the toxicity of the fluorophore was retained in the conjugates: As reporters for following in vivo uptake or catabolism of cholesterol, or as “Trojan horse” antibiotics. The results pointed out that the new compounds (**342**) emitted blue light in nonpolar solvents, and its lipid portion incorporated into liposomal membrane bilayers quickly, leaving the fluorophore exposed to the external aqueous environment. Compounds in **342** were incubated with *Mycobacterium smegmatis* mc2 155, which displayed stable integration of the fluorescent-labeled cholesterols into bacterial membranes in vivo. Although fluorophores are toxic to prokaryotic cells, the new cholesterol conjugates (**342**) are not, and therefore they could be considered for the evaluation of cholesterol uptake in prokaryotic organisms [88].

**Scheme 83:**
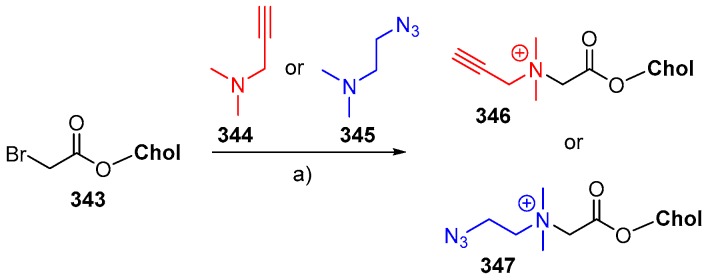
Synthesis of cholesteryl-ammonium compounds. Reagents and conditions: **a**) acetone, reflux, 3 h.

In the same year, Bernhard et al. reported an interesting paper in which they studied two strategies for the bioconjugation of bombesin (BBN), a well-known peptide, the receptor of which is overexpressed at the surface of tumor cells and which has been conjugated in several probes [89]. They used subphthalocyanines (SubPcs), which are interesting probes for optical imaging. One of these strategies involved the entrapping of SubPc into a liposome and subsequently grafting BBN to the SubPc-containing liposome to afford a biovectorized liposome. The synthesis of cholesterol derivatives **346** and **347** used in their work was achieved by the reaction of dimethylaminopropyne **344** or 3-azidodimethylpropylamine **345** with cholesterol bromo ester **343** to afford cholesteryl-ammonium species **346** (alkynyl) and **347** (azide), respectively (Scheme 83) [89].

**Scheme 84:**
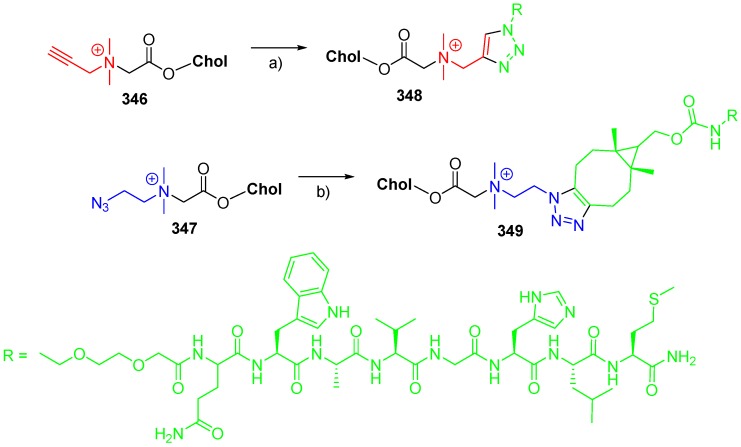
Synthesis of bombesin (BBN)-cholesterol conjugates. Reagents and conditions: **a**) *N*-2-2-[2-(*N*-azidoethoxy)ethoxy]acetyl-bombesin, CuSO_4_·5H_2_O, AscONa, DMF, rt, 8 h; **b**) *N*-2-[2-(2-(*N*-4-(((bicyclo[6.1.0]non-4-yn-9-ylmethoxy)carbonyl)amino)ethoxy)ethoxy]acetyl-bombesin, DMF, rt, 15 h.

Once the cholesteryl-ammonium species **346** and **347** were prepared, the pre-bioconjugation strategy started from grafting the biomolecule to one liposome’s component (i.e., cholesterol additive) prior to the preparation of the liposome, to afford BBN-cholesterol conjugates **348** and **349**. The conjugation of BBN-azide with cholesteryl-alkyne **346** (i.e., pre-functionalization by copper-catalyzed click chemistry) was carried out in the presence of copper sulfate and sodium ascorbate as the reducing agents (Scheme 84) [89]. Alternatively, BBN-bicyclononyne and cholesteryl-azide **347** were reacted without the Cu catalyst to afford conjugate **349** (Scheme 84) [89]. This strategy was employed using liposomes containing graftable cholesterol derivatives, revealed itself as a more suitable approach in addressing the stability of SubPcs, and was achieved by copper-free click-chemistry on the outer face of the liposome. This study demonstrated that both azido- and alkynyl-liposomes are good entry points for a bioconjugation or biovectorization approach (on the outer face of the liposome), which offers a second chance for fluorophores with no reactive functional group available on their backbone, a way of imitating bioconjugation with a biomolecule (i.e., an indirect approach offered to achieve future site-specific targeting of tumors) [89].

**Scheme 85:**
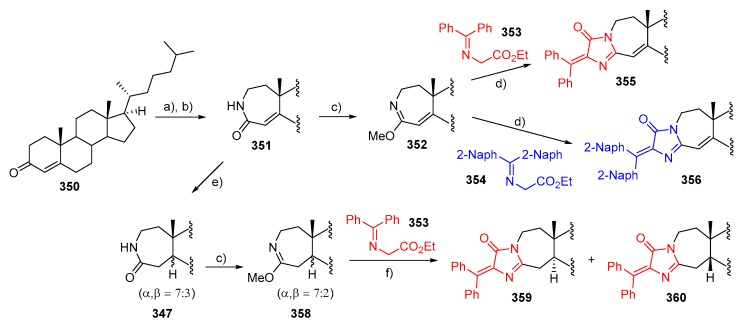
Synthesis of Ch-DAINs. Reagents and conditions: **a**) NH_2_OH·HCl, NaOAc, EtOH, reflux; **b**) phenylpropanolamine (PPA), 130 °C, 2 steps; **c**) MeOTf, CH_2_Cl_2_, rt, 24 h; **d**) AcOH, toluene, 50 °C, 48 h; **e**) Pd/C (10%), MeOH, rt, 7 days; **f**) AcOH, toluene, reflux, overnight.

A series of new hybrid compounds (Ch-DAINs), **355**, **356**, and **360**, bearing a green fluorescent protein-chromophore analogue, 4-(diarylmethylene)imidazolinone (DAIN), and a cholesten or cholestane, was recently reported as a candidate for viscosity-dependent and cholesterol-responsive fluorescent molecules [90]. The synthesis of Ch(en)-DAINs **355** and **356** was carried out through a condensation reaction of methyl imidates **352** or **358** (obtained from cholestenone through Beckmann rearrangement followed by methylation) with *N*-(diarylmethylene) glycinates **353** or **354** (Scheme 85) [90]. Likewise, Ch(an)-DAINs **359** and **360** were obtained following the same synthetic strategy with an additional double-bond hydrogenation step (Scheme 85) [90].

Among the tested compounds, cholesten DAINs **355** and **356** increased their fluorescence intensity in viscous solvents such as triglycerides. Besides, compound **355** showed good cholesterol-responsive emission, which increased linearly with the amount of cholesterol in the lipid bilayer. The responsiveness displayed by cholesten DAIN **355** to cholesterol was improved relatively to the known viscosity probes, 9-(2,2-dicyanovinyl)julolidine (DCVJ) and Laurdan [90].

## 7. Synthetic Applications

**Scheme 86:**
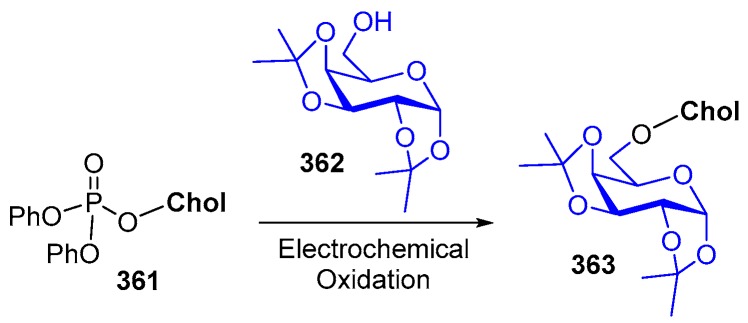
Electrochemical oxidation of cholesteryl diphenylphosphate in the presence of 1,2:3,4-di-*O*-isopropylidene-α-d-galactopyranose.

The regio- and stereoselective formation of *O*-glycosidic bonds between carbohydrates and steroids is still a demanding process, despite the considerable progress in carbohydrate chemistry in the last years. The direct electrochemical glycosylation of steroids is an alternative: However, it has several drawbacks. In attempting to solve the problem, Tomkiel et al. screened several derivatives of cholesterol as sterol donors in electrochemical reactions with sugar alcohols [91]. The authors tested sixteen cholesterol substrates in the presence of 1,2:3,4-di-*O*-isopropylidene-α-d-galactopyranose (**362**), concluding that cholesteryl diphenylphosphate **361** was the best compound for the purpose, affording 3β-*O*-(1′,2′:3′,4′-di-*O*-isopropylidene-α-d-galactopyranos-6′-yl)-cholest-5-ene (**363**) in 54% yield (Scheme 86) [91].

**Scheme 87:**
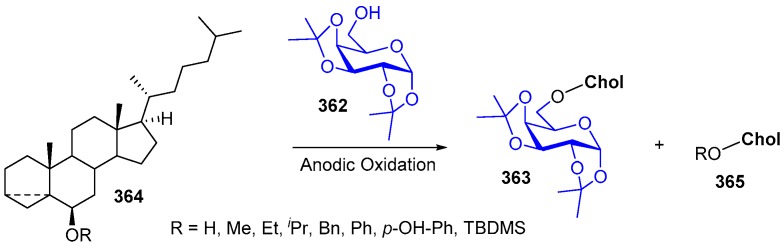
Anodic oxidation of 3α,5α-cyclocholestan-6β-yl alkyl and aryl ethers.

Following this work, the same authors reported in 2015 the use of 3α,5α-cyclocholestan-6β-yl alkyl and aryl ethers (**364**) as a cholesteryl donor in the electrochemical synthesis of glycoconjugates (**363**) (Scheme 87) [92]. The reaction worked well for all the tested compounds, but the best yields were achieved for ethyl, benzyl, phenyl, and *tert*-butyldimethylsilyl (TBDMS) ethers (51%, 50%, 58%, and 52%, respectively). Unfortunately, an isomerization side reaction was observed for the less reactive cholesteryl esters, affording the compounds in **365** (Scheme 87) [92].

**Scheme 88:**
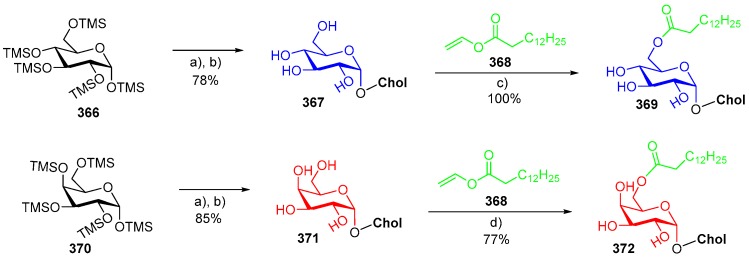
Synthesis of cholesteryl α-d-glucopyranoside and its enzymatic regioselective acylation. Reagents and conditions: **a**) iodotrimethylsilane (TMSI), OH-Chol, tetrabutylammonium iodide (TBAI), DIPEA, 4 Å molecular sieves, CH_2_Cl_2_, rt, 48 h; **b**) Dowex-50WX8-200, MeOH, 2 h, rt; **c**) Novozym 435, acetone, 40 °C, 24 h; **d**) Novozym 435, THF/pyridine (4:1), 40 °C, 96 h.

To develop step-economy syntheses of cholesteryl glycosides, Davis and coworkers reported a methodology for the synthesis of α-d-cholesteryl glycosides **369** and **372**, using a one-pot per-*O*-trimethylsilyl glycosyl iodide glycosylation (Scheme 88) [93]. The methodology relied first on the generation of glucosyl or galactosyl iodide through the reaction of per-*O*-TMS glucoside **366** or **370** with iodotrimethylsilane (TMSI), which was directly cannulated into a solution of cholesterol, tetrabutylammonium iodide (TBAI), and *N*,*N*-diisopropylethylamine (DIPEA), and the mixture was stirred for 2 days at room temperature. After that, the product was treated with methanol and Dowex-50WX8-200 acidic resin to remove the silyl protecting groups, affording compounds **367** and **371** (Scheme 88) [93]. These glycosides were subsequently esterified using regioselective enzymatic acylation of the 6-hydroxy group with tetradecanoyl vinyl ester **368** (Scheme 88) [93].

This methodology involving the glycosylation of cholesterol followed by enzymatic regioselective acylation allowed expansion of the acylated α-cholesteryl glycoside inventory to include galactose analogues. The glycosylation of per-*O*-silylated glucose provided better α-selectivity (39:1) than past syntheses (8:1 α-selectivity) and higher glycosylation yields due to the armed nature of per-*O*-silyl donors [93].

**Scheme 89:**
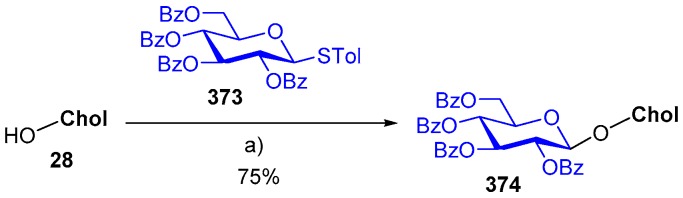
Glycosylation of cholesterol. Reagents and conditions: **a**) Cu(OTf)_2_, activated 4 Å molecular sieves, CH_2_Cl_2_, rt, 5 days.

Mao and coworkers developed a novel glycosyl coupling reaction, involving a photoinduced direct activation mechanism of thioglycosides (**373**) and subsequent *O*-glycosylation in the absence of photosensitizer [94]. In their studies, the authors used several sugars, amino acids, and cholesterol **28** (75%) as substrates (Scheme 89). The authors showed that the activation of thioglycosides upon UV irradiation followed by the oxidation of Cu(OTf)_2_ led to the in situ formation of species that could undergo glycosylation to afford glycosides without the need for a photosensitizer. The proposed mechanism involved i) homolytic cleavage of a C-S bond to generate a glycosyl radical and ii) oxidation to an oxacarbenium ion promoted by Cu(OTf)_2_, and sequential *O*-glycosylation [94].

**Scheme 90:**
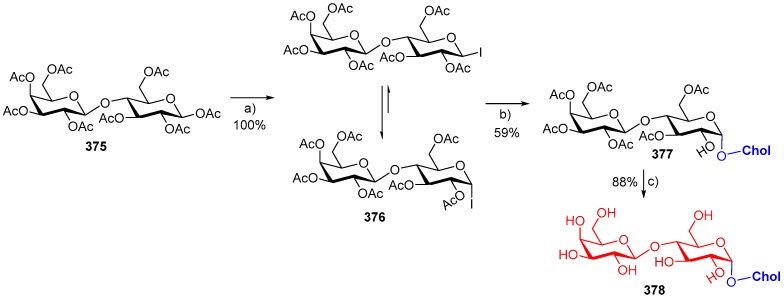
Glycosylation of cholesterol. Reagents and conditions: **a**) TMSI, CHCl_3_, 10 min; **b**) cholesterol, CHCl_3_, MW, 110 °C, 2 h; **c**) NaOMe, MeOH/CH_2_Cl_2_ (10:1), rt, 2 h.

In 2015, Davis and coworkers reported the synthesis of cholesteryl-α-d-lactoside **378** via generation and trapping of stable β-lactosyl iodide **376**. The iodide derivative **376** was prepared quantitatively under non-in situ anomerization and metal-free conditions by reacting commercially available β-per-*O*-acetylated lactose **375** with trimethylsilyl iodide [95]. The introduction of cholesterol occurred under microwave conditions to afford the corresponding glycoconjugate **377** in 59% yield (Scheme 90). Cholesterol glycoconjugate **377** was further deacetylated using sodium methoxide to afford cholesteryl α-d-lactoside **378** in 88% yield (Scheme 90). This glycosylation method can be employed on sterically demanding nucleophiles such as cholesterol and has potential applications in accessing structurally diverse cholesteryl glycoside analogs [95].

**Scheme 91:**
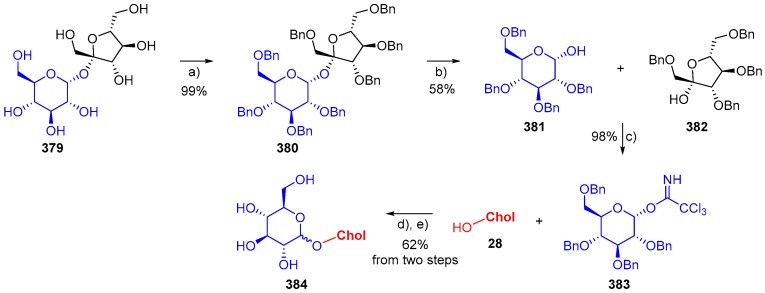
Synthesis of cholesteryl glucoside starting from sucrose. Reagents and conditions: **a**) BnBr, NaH, DMF, rt, 4.5 h; **b**) conc. HCl, acetone, reflux, 1.5 h; **c**) trichloroacetonitrile, NaH, CH_2_Cl_2_, rt, 4 h; **d**) TMSOTf, 4 Å molecular sieves, CH_2_Cl_2_, rt, 1.5 h; **e**) Pd(OH)_2_, EtOH/cyclohexene (2:1), reflux.

A new efficient method for the synthesis of cholesteryl glucosides starting from sucrose **379** was recently developed [96]. This method lays down a five-step synthetic route that involves the initial protection of disaccharide **379** hydroxy groups, and upon acidic hydrolysis at its anomeric center, the pyranosyl moiety **381** is converted into trichloroacetimidate derivative **383** (Scheme 91).

The final two steps rely on the formation of the glycosidic bond to cholesterol **28** followed by the removal of the protecting groups, affording the desired cholesteryl glucoside **384** (Scheme 91). The authors claimed that the major advantage of this strategy was the use of the readily available and cheap sucrose **379** as starting material. In addition, the methodology proved to be fast, cost-saving, and high-yielding, representing a competitive preparation method for these natural compounds [96].

**Scheme 92:**
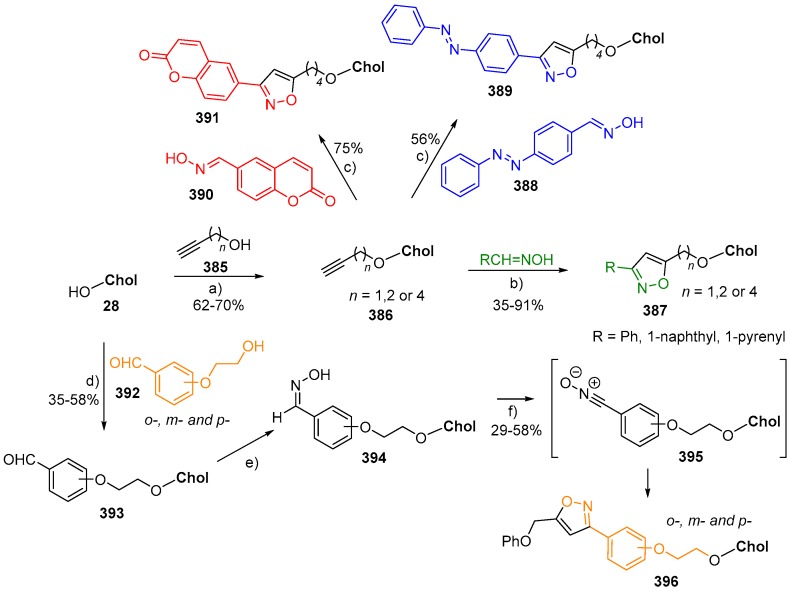
Synthesis of 3,5-disubstituted isoxazoles. Reagents and conditions: **a**) MK-10, CHCl_3_, MW, 90 °C, 17 h; **b**) Ch-T, EtOH, *n* = 1 MW at 60–100 °C for 30 min to 1 h and *n* = 2 or 4 rt for 1 h; **c**) Ch-T, EtOH, rt, 1 h; **d**) MK-10, CHCl_3_, MW, 90 °C, 8 h; **e**) NH_2_OH·HCl, pyridine, EtOH, MW, 125 °C, 1 h; **f**) (prop-2-yn-1-yloxy)benzene, Ch-T, EtOH, rt, 17 h.

In 2014, Algay and coworkers extensively explored the versatility of nitrile oxide alkyne cycloaddition (NOAC) chemistry for the formation of cholesterol conjugates anchored by way of a polar, aromatic, metabolically stable isoxazole nucleus [97]. The first series of compounds produced in this paper involved i) the microwave-assisted formation of propargyl ethers (**386**) in 62%–70% yield (Scheme 92a), and ii) the reaction of cholesterol propargyl ethers (**386**) with phenyl nitrile oxide (generated in situ from benzaldehyde oxime upon exposure to an ethanolic solution of chloramine-T) (Scheme 92b). This last reaction was carried out at room temperature or under microwave heating depending on the length of the spacing between the bulky lipid and the reacting alkyne, affording isoxazoles (**387**) in fair to excellent yields (35%–91%) [97]. The authors extended a bit further this reaction to prepare biologically relevant cholesterol fluorescent probes such as steroid–coumarin (**391**) (75%) and steroid–azobenzene conjugates (**389**) (56%) (Scheme 92). It is known that long-chain hydrophilic linkers are very attractive for bioconjugation and therefore, in this paper, the authors also synthesized three new ether-linked isoxazole-cholesterol conjugates (**396**) in 29%–58% yield (Scheme 92) [97].

**Scheme 93:**
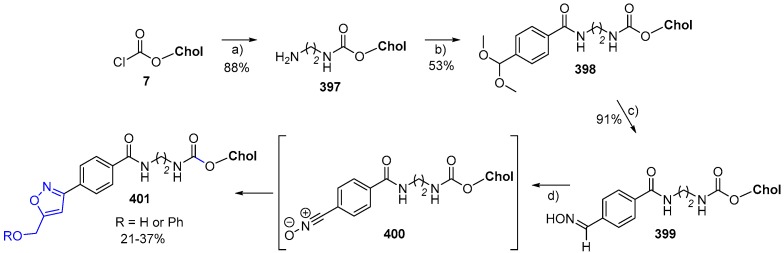
Synthesis of amidocarbamate cholesterol conjugates. Reagents and conditions: **a**) ethylenediamine, toluene, rt, 17 h; **b**) 4-(dimethoxymethyl)benzoic acid, DCC, DMAP, CH_2_Cl_2_/toluene, rt, 17 h; **c**) NH_2_OH·HCl, EtOH, MW, 125 °C, 1 h; **d**) Ch-T, propargyl alcohol, or phenyl propargyl ether, EtOH, rt, 17 h.

Another series of isoxazole-cholesterol conjugates (**401**) was also prepared, starting from cholesterol chloroformate **7** and bearing an amidocarbamate linker following the four-step synthetic route depicted in Scheme 93 [97].

**Scheme 94:**
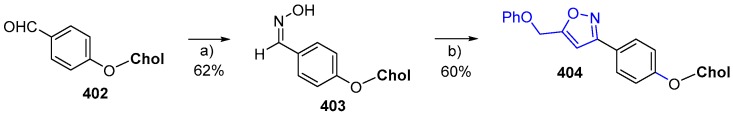
Synthesis of isoxazole-linked aryl cholesterol ether. Reagents and conditions: **a**) NH_2_OH·HCl, pyridine, EtOH, MW, 125 °C, 1 h; **b**) (prop-2-yn-1-yloxy)benzene, Ch-T, EtOH, rt, 17 h.

The nontrivial synthesis of aryl ethers of natural alcohols drove the authors to test the NOAC chemistry in the assembly of aryl ether cholesterol conjugates [97]. Therefore, isoxazole-linked aryl cholesterol ether **404** was prepared from the aldehyde-functionalized aryl ether **402** through subsequent oximation and cycloaddition reactions, as depicted in Scheme 94.

**Scheme 95:**
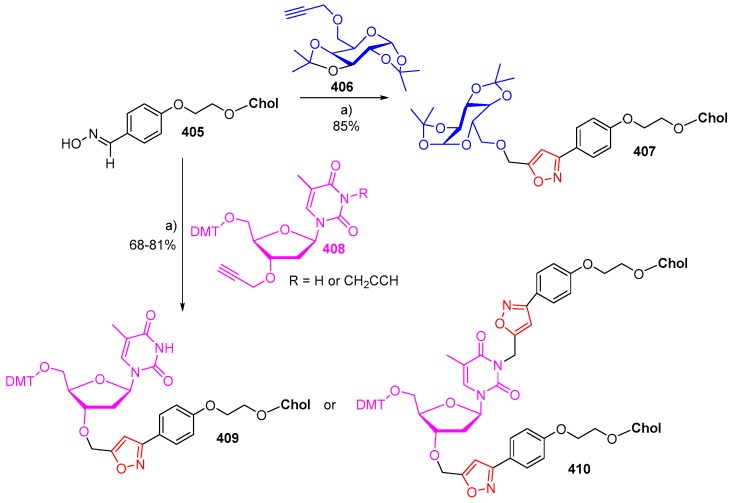
Synthesis of glycol- and thymidine-cholesterol conjugates. Reagents and conditions: **a**) Ch-T, EtOH, rt, 17 h.

Finally, the authors used the potential of NOAC chemistry to prepare a steroidal glycoconjugate, **407**, and the selective tethering of one or two cholesterol units, **409** and **410**, respectively, to a thymidine skeleton was demonstrated by trapping of the same dipole by 5′-protected mono- or bis-propargylated thymidines (Scheme 95) [97].

**Scheme 96:**
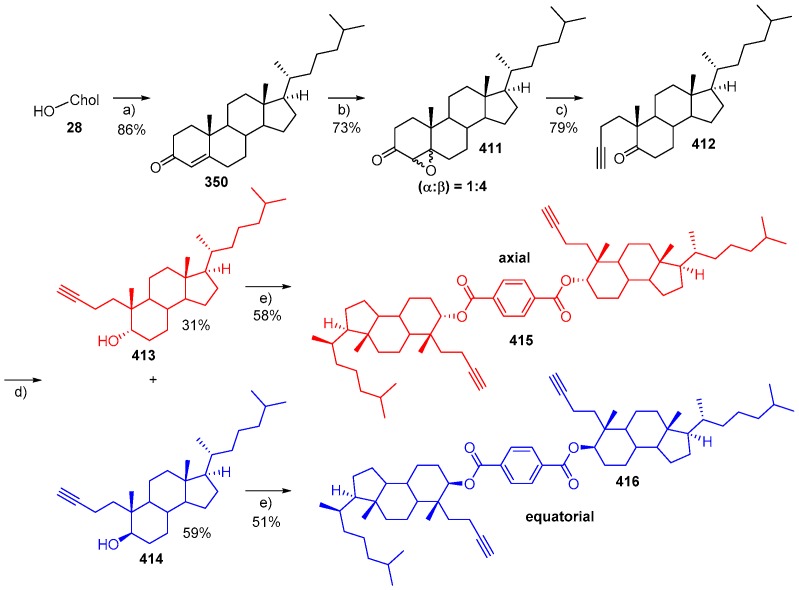
Synthesis of dimeric steroidal terephthalates. Reagents and conditions: **a**) Al(O-*i*-Pr)_3_, cyclohexanone, toluene, reflux, 1.5 h; **b**) NaOH/MeOH (10%), H_2_O_2_ (30%), CH_2_Cl_2_, rt, 72 h; **c**) TsNHNH_2_, CH_2_Cl_2_/AcOH (1:1), rt, 2.5 h; **d**) NaBH_4_, MeOH, rt, 30 min; **e**) terephthaloyl chloride, DMAP, Et_3_N, toluene, reflux, 5 h.

In 2016, Alarcón-Manjarrez and coworkers reported the synthesis of two dimeric steroidal terephthalates, **415** and **416**, from epimeric 4,5-seco-cholest-3-yn-5-ols **413** and **414**, using a five-step synthetic route with cholesterol **28** as a starting material [98]. The synthetic route first involved the Oppenauer oxidation of cholesterol **28**, followed by epoxidation, to afford a mixture of epoxides (**411**) (α:β = 1:4) (Scheme 96). Then, an Eschenmoser-Tanabe fragmentation followed by carbonyl group reduction provided the epimeric alkynols **413** and **414** in a 1:2 ratio (Scheme 96). Finally, the treatment of each one of the epimeric alkynols **413** and **414** with terephthaloyl chloride led to the symmetrical axial and equatorial dimers **415** and **416**, respectively (Scheme 96) [98].

The authors proceeded to crystallographic analysis of the compounds and concluded that the facial hydrophobicity of the steroidal skeletons had crucial influence on the crystal packing in which the dimeric molecules were forced to accommodate these fragments only with a few hydrogen-bonding interactions. This feature originated a cisoid conformation for **415** and a linear conformation for **416** [98].

**Scheme 97:**
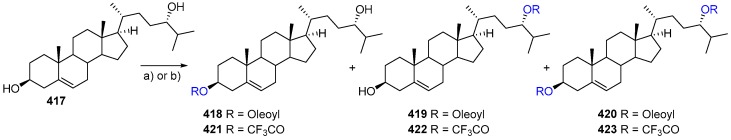
Synthesis of (24*S*)-OHChol oleates and trifluoroacetates. Reagents and conditions: **a**) *cis*-oleoyl chloride, Et_3_N, DMAP, THF, 0 °C to rt, 16 h; **b**) 2-trifluoroacetoxypyridine, CH_2_Cl_2_, rt, 20 h.

**Scheme 98:**
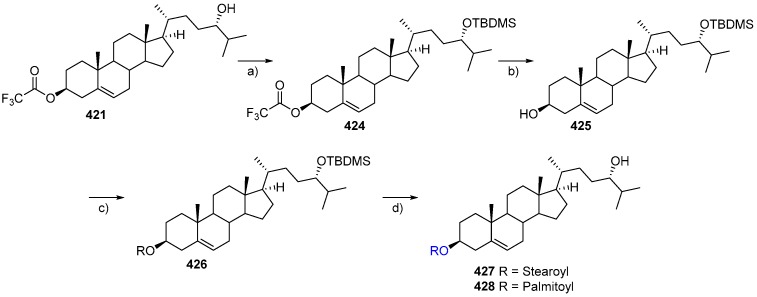
Synthesis of (24*S*)-OHChol stearoyl and palmitoyl esters. Reagents and conditions: **a**) TBDMSCl, imidazole, DMF, rt, 16 h; **b**) 7 N NH_3_ in MeOH, rt, 3 h; **c**) stearoyl chloride or palmitoyl chloride, Et_3_N, DMAP, THF, 0 °C to rt, 16 h; **d**) HF.pyridine, AcOH, 50 °C to rt, 16 h.

**Scheme 99:**
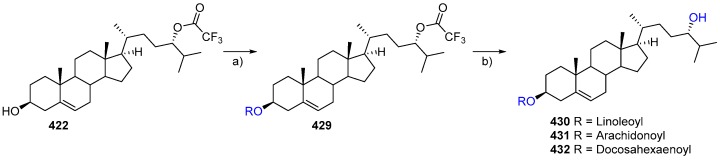
Synthesis of (24*S*)-OHChol linoleoyl, arachidonoyl and docosahexaenoyl esters. Reagents and conditions: **a**) linoleoyl chloride, arachidonoyl chloride or docosahexaenoyl chloride, Et_3_N, DMAP, THF, 0 °C to rt, 16 h; **b**) 7 N NH_3_ in MeOH, 0 °C, 3 h.

Shibuya et al. reported in 2016 the synthesis of (24*S*)-hydroxycholesterol (24*S*-OHChol) esters, which are involved in neuronal cell death, through catalysis with acyl-CoA:cholesterol acyltransferase-1 (ACAT-1) [99]. The authors studied the esterification of (24*S*)-OHChol **417** with *cis*-oleoyl chloride under basic conditions and obtained mono-oleates **418** and **419** and bis-oleate **420** in 39%, 9%, and 20% yields, respectively (Scheme 97). The protection of (24*S*)-OH with a trifluoroacetyl group was also attempted, affording mono-trifluoroacetates **421** and **422** in 33% and 14% yields, respectively, and the bis-trifluoroacetate **423** in 21% yield (Scheme 97) [99]. The authors took advantage of the mono-trifluoroacetate **421** to prepare the stearoyl and palmitoyl esters **427** and **428** in 68% and 75% yields, respectively, as depicted in Scheme 98 [99]. Finally, the authors also reported the use of esters of unsaturated long-chain fatty acids, such as linoleic (LA), arachidonic (AA), and docosahexaenoic (DHA), to react with cholesterol derivative **422** in order to prepare linoleate **430**, arachidonoate **431,** and docosahexaenoate **432** esters, in 52%, 74%, and 66% yields, respectively, in a two-step synthetic route, as depicted in Scheme 99 [99].

**Scheme 100:**
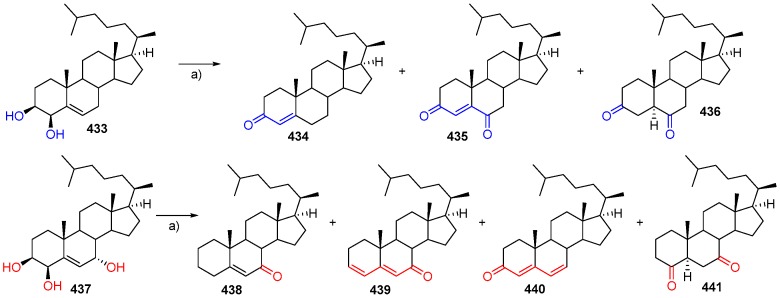
Oxo-functionalization of 4β-hydroxycholesterol. Reagents and conditions: **a**) *p*-TSA/SiO_2_, 120 °C, 5 or 10 min.

Recently, Sarkar et al. reported interesting work dealing with the preparation of diverse ring-A or ring-B oxo-functionalized steroids in a green fashion involving solvent-free solid supports [100]. The authors used cholesterol derivatives such as 4β-hydroxycholesterol **433**, which was functionalized into three different keto-steroids, **434**, **435**, and **436**, in 55%, 10%, and 10% yields, respectively, employing *p*-toluenesulfonic acid and SiO_2_ (silica 60–120 mesh) as solid support (Scheme 100) [100]. Interestingly, if the reaction was attempted in the solution phase at room temperature using either dichloromethane or ethanol as solvents, cholest-4-en-3-one **434** was obtained exclusively in 64% and 60% from dichloromethane and ethanol, respectively. The procedure on solid silica was applied to the other cholesterol derivative, namely 4β,7α-dihydroxycholesterol **437**, which was converted into four distinct keto-steroids: (i) cholest-5-en-7-one (**438**, 8%), (ii) cholesta-3,5-dien-7-one (**439**, 13%), (iii) cholesta-4,6-dien-3-one (**440**, 17%), and (iv) 5a-cholestane-4,7-dione (**441**, 7%) (Scheme 100) [100]. It is worth noticing that if the reaction of 4β,7α-dihydroxycholesterol **437** was carried out in dichloromethane as solvent, cholesta-4,6-dien-3-one (**440**, 54%) was found to be the only product formed. This was found to be a facile procedure for the synthesis of dienone **440** from cholesterol via triol **437** [100].

**Scheme 101:**
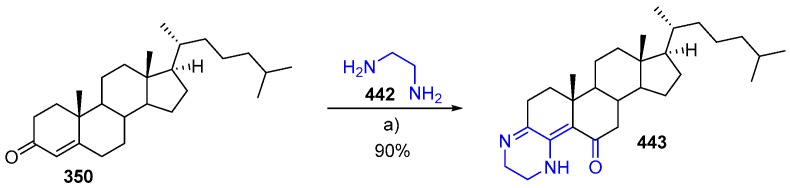
Synthesis of 4′-dehydrocholest-4-eno[3,4-*e*]piperazin-6-one. Reagents and conditions: **a**) basic Al_2_O_3_, MW, 120 °C, 5 min.

Cholesterol derivatives can also be used as starting materials for the synthesis of fused nitrogen heterocycles. This was the case for 4-cholesten-3-one **350**, which was involved in the preparation of A-ring dehydropiperazine **443** (90% yield) through a microwave-assisted annulation reaction with ethylenediamine **442** in the presence of basic alumina (Scheme 101) [101].

The proposed mechanism should encompass the initial oxidation of the allylic protons of the conjugated ketone via enolate intermediate to afford a diketo intermediate. Then, the condensation with ethylenediamine followed by a Michael addition and autoxidation reactions afforded the dehydropiperazine derivatives [101].

**Scheme 102:**
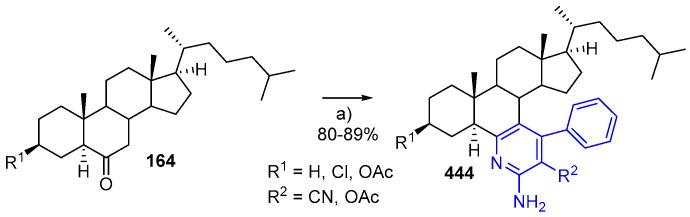
Synthesis of steroidal fused pyridines. Reagents and conditions: **a**) benzaldehyde, malononitrile or methylcyanoacetate, MgO nanoparticles (NPs), EtOH, MW, 70 °C, 20–25 min.

Recently, Ansari and coworkers reported an efficient and green synthetic method for the preparation of steroidal pyridines [102]. Such methodology relied on the utilization of MgO NPs as a heterogeneous, mild, and reusable catalyst, in a multicomponent one-pot protocol, taking advantage of the usefulness of the microwave irradiation as an alternative heating source. The series of substituted fused pyridines (**444**) were obtained in 80%–89% yield from the reaction of steroidal ketones (**164**) with malononitrile/methylcyanoacetate, benzaldehyde, and ammonium acetate in ethanol using MgO NPs as a catalyst (Scheme 102) [102].

One of the key mechanistic steps in this kind of multicomponent reaction is the standard Knoevenagel condensation of benzaldehyde and malononitrile/methyl cyanoacetate. The effect of MgO NPs can be rationalized on this basis since they are known as a highly effective heterogeneous base catalyst for Michael addition and Knoevenagel condensation reactions with Mg^2+^ (Lewis acid) and O^2−^ (Lewis base) sites along with various cationic and anionic vacancies in the lattice [102].

**Scheme 103:**
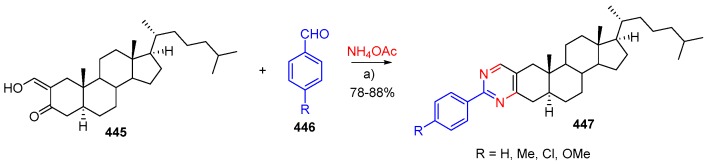
Synthesis of cholesterol-fused pyrimidines. Reagents and conditions: **a**) NH_4_OAc, silica gel (60–120 mesh), MW, 120 °C, 6 min.

The A-ring of cholesterol was also functionalized by fusing pyrimidines at the steroidal 2,3-position. These new steroidal compounds were synthesized through a microwave-assisted three-component reaction of 2-hydroxymethylene-3-ketosteroid (**445**), benzaldehydes (**446**), and ammonium acetate, affording cholesterol-fused pyrimidines (**447**) in good yields (78%–88%) (Scheme 103) [103].

The authors’ mechanism was based on: (i) microwave-assisted reaction of ammonia (released from decomposition of ammonium acetate) with 2-hydroxymethylene-3-ketosteroid to afford a β-aminoketoimine intermediate; (ii) their condensation reaction with benzaldehydes to afford a diamine intermediate; and (iii) cyclization and subsequent auto-oxidation to give the cholesterol-fused pyrimidines [103].

**Scheme 104:**
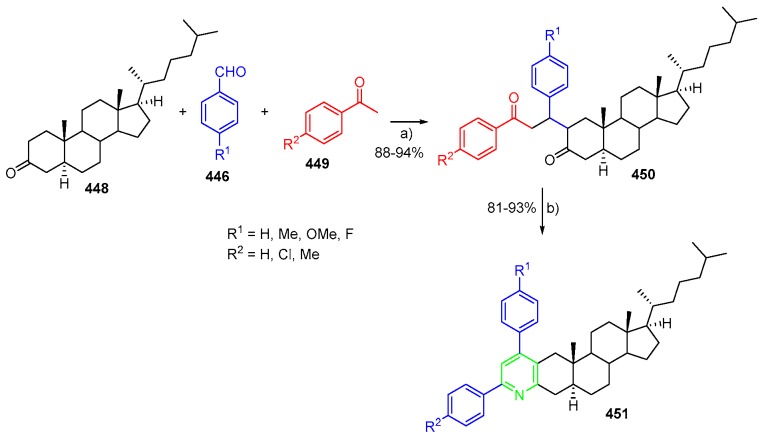
Synthesis of cholesterol-fused 4,6-diarylpyridines. Reagents and conditions: **a**) KOH, toluene, rt, 6 h; **b**) urea, BF_3_·OEt_2_, MW, 140 °C, 8 min.

A two-step method for the preparation of steroid-fused 4,6-diaryl substituted pyridines has been reported [104]. The synthetic protocol relied on the Michael addition of 5α-cholestan-3-one **448** with chalcones (generated in situ by the base-catalyzed reaction of acetophenones (**449**) and benzaldehydes (**446**)), affording 3,5-diaryl-1,5-dicarbonyl 5α-cholestan-3-one derivatives (**450**) (88%–94%) (Scheme 104). Then, the intermediates (**450**) were used as substrates in a microwave-assisted solid phase reaction with urea in the presence of BF_3_·OEt_2_ to give 4,6-diaryl substituted pyridines (**451**) in good yields (81%–93%) (Scheme 104) [104].

The authors proposed a mechanism for the formation of a pyridine ring, which may start with the release of ammonia by urea under microwave heating, which forms an imine by reaction with one carbonyl group. Next, the BF_3_·OEt_2_-promoted nucleophilic attack of the imine NH-group on the activated carbonyl functionality facilitated an aza-cyclization reaction, affording a 1,4-dihydropyrinine intermediate upon which aromatization gave the desired 4,6-diarylpyridines [104].

**Scheme 105:**
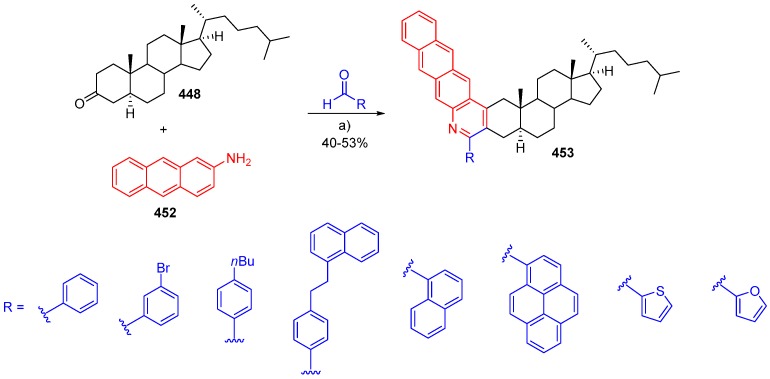
Synthesis of model asphaltene compounds. Reagents and conditions: **a**) I_2_, H_2_O/THF, reflux, 5 days.

In 2015, Schulze and coworkers developed a new method for the synthesis of model asphaltene compounds. The reported methodology was based on a multicomponent cyclocondensation reaction of 2-aminoanthracene **452** with aromatic aldehydes and 5-α-cholestan-3-one **448** (Scheme 105) [105].

The authors found that the actual catalyst for this reaction was indeed hydriodic acid, which is formed in situ from the reaction of iodine with water. Carrying the reaction under anhydrous conditions, it was proven that iodine itself did not promote the reaction, as generally assumed. Using this methodology, the authors prepared a library of optically active steroidal naphthoquinolines (**453**) in acceptable yields (40%–53%) [105].

## 8. Miscellaneous

**Scheme 106:**
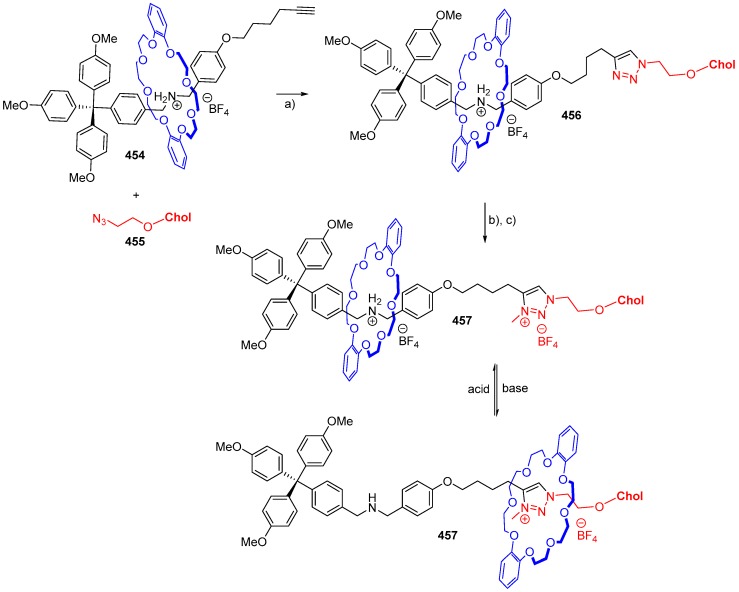
Synthesis of cholesterol-based rotaxane. Reagents and conditions: **a**) Cu(CH_3_CN)_4_BF_4_, tris[(1-benzyl-1*H*-1,2,3-triazol-4-yl)methyl]amine, CH_2_Cl_2_, rt, 48 h; **b**) MeI, rt, 72 h; **c**) NaBF_4_, CH_2_Cl_2_/acetone/H_2_O (1:1:2), rt, 18 h.

The design of (supra)molecular switches and machines has a key feature that relates to the control of mechanical motions at the molecular level. In this field, rotaxanes have attracted much attention because they offer the possibility of restricting the freedom of motion to some well-defined pathways, such as the translational motion of a rotaxane’s ring along its axis in a shuttling manner. The synthesis of a novel nonsymmetrical bistable pH-sensitive rotaxane with a cholesterol stopper at one end and a tetraphenylmethane group at the other end (**457**), has been reported [106]. The synthesis of both terminal ends was challenging, and therefore we only describe here the final step, which consisted of joining both axes of the nonsymmetrical rotaxane, the alkyne **454**, and the azide **455** through CuAAC chemistry, affording compound **456** (Scheme 106). The formation of a pH-sensitive bistable rotaxane **457** was achieved by methylation of the triazole ring using methyl iodide (Scheme 106). The authors verified that the crown ether part changed its preferred position on the axis because of the protonation state of a secondary amine. More specifically, the crown ether was located around the secondary ammonium ion as the best binding site in the protonated form. On the other hand, NMR analysis showed that upon deprotonation of the ammonium ion, the triazolium ion became the better binding site, which caused the ring to shuttle along the axis toward this position (Scheme 106) [106].

**Scheme 107:**
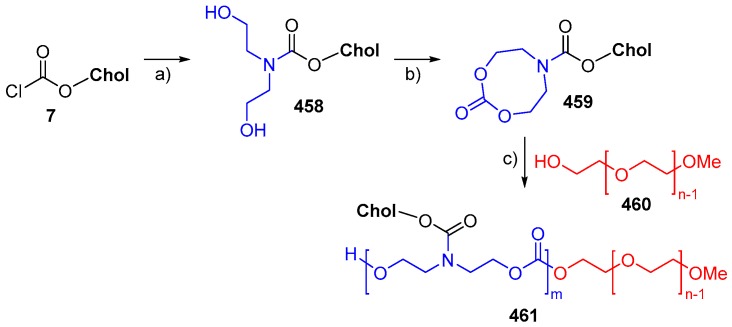
Synthesis of PEGylated amphiphilic diblock copolymers. Reagents and conditions: **a**) diethanolamine, Na_2_CO_3_, THF/H_2_O (2:1), 0 °C to rt, 16 h; **b**) ethyl chloroformate, Et_3_N, THF, 0 °C to rt, 16 h; **c**) 1,8-diazabicyclo[5.4.0]undec-7-ene (DBU), CH_2_Cl_2_, rt, 2 h.

Venkataraman and coworkers reported the two-step synthesis of cholesterol-functionalized aliphatic *N*-substituted 8-membered cyclic carbonate monomer **459** (Scheme 107) [107]. Cholesterol-based monomer **459** was employed in organocatalytic ring-opening polymerization to produce PEGylated amphiphilic diblock copolymers (using a commercially available macroinitiator), polyethylene glycol monomethyl ether (mPEG-OH) **460** (Scheme 107). The authors evaluated the behavior of these copolymers in aqueous media, concluding that they self-assembled to form unique nanostructures, including disk-like micelles. The experimental results also suggested that the prepared copolymers can be used as inexpensive steric stabilizers for liposomes, making them suitable for several biomedical applications [107].

**Scheme 108:**
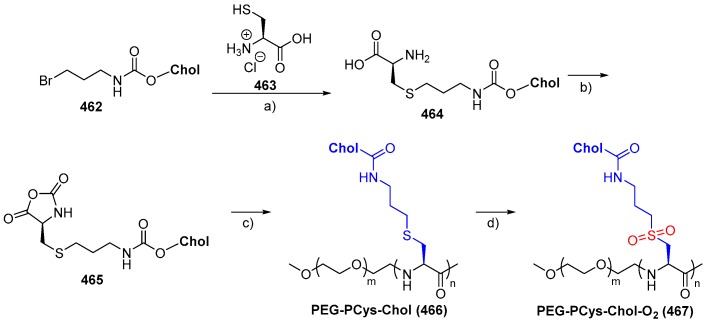
Synthesis of copolymers PEG-PCys-Chol and PEG-PCys-Chol-O_2_. Reagents and conditions: **a**) 2 N NaOH, TBAI, CHCl_3_/EtOH (7:26), rt, 48 h; **b**) triphosgene, THF, 50 °C, 4 h; **c**) PEG-NH_2_, THF, 35 °C, 72 h; **d**) 10% H_2_O_2_, 5% AcOH, 37 °C, 16 h, dialysis.

Recently, a cholesterol-modified poly(l-cysteine) copolymer, **466**, that can undergo unusual micelle-to-vesicle transformation of polypeptides triggered by oxidation, was synthesized following a three-step protocol starting from cholesteryl 3-bromopropylcarbamate **462** (Scheme 108) [108]. The thioether groups in the side chains of **466** were further oxidized to the corresponding sulfone derivative **467** (Scheme 108). The authors demonstrated that oxidation of the thioether groups in the side chains could change the packing characteristics of cholesterol groups and the peptide backbone, resulting in the transformation of a β-sheet to an α-helix conformation, combined with an interesting morphological transition from micelle-like structures to vesicles. Moreover, changing the secondary structure as well as the morphology endowed the polymer assemblies with excellent specificity for controlled payload release and improved cell interaction in response to ROS. These interesting formulations had excellent anticancer properties both in vitro and in vivo [108].

**Scheme 109:**
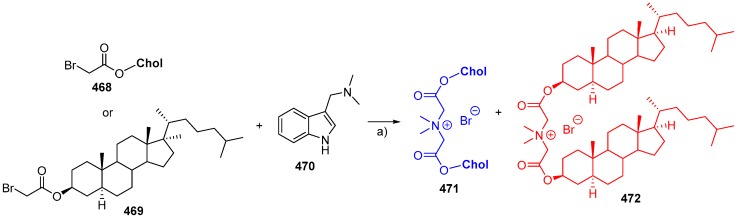
Synthesis of cholesterol and cholestanol dimers. Reagents and conditions: **a**) MeCN, reflux, 5 h.

Gramine [*N*-(1*H*-indol-3-ylmethyl)-*N*,*N*-dimethylamine] is a well-known indole derivative and is often used as synthon for the preparation of a large variety of substituted indoles with important biological activities. In this context, Kozanecka and coworkers reported the use of gramine (**470**) to synthesize cholesterol (**471**) and cholestanol dimers (**472**) consisting of two molecules of sterols connected by an N(CH_3_)_2_ group (Scheme 109) [109].

These new steroid dimers (**471** and **472**) were shown to interact in vitro with the human erythrocyte membrane, changing the discoid erythrocyte shape, which resulted in induced stomatocytosis or echinocytosis. The authors also demonstrated that these new dimers were capable of interfering with membrane phospholipid asymmetry and loosening the molecular packing of phospholipids in the bilayer at sublytic concentrations. Moreover, the dimers **471** and **472** possessed a higher capacity for changing the erythrocyte membrane structure and its permeability than steroids alone did [109].

**Scheme 110:**
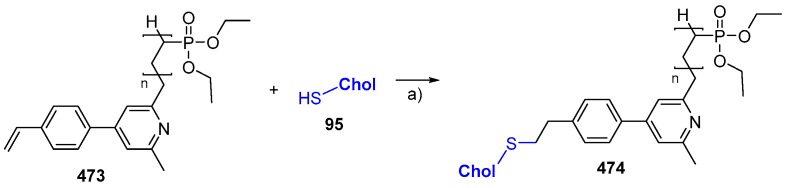
Synthesis of poly-DEVP thiocholesterol. Reagents and conditions: **a**) AIBN, 70 °C, 24 h. DEVP: Diethylvinylphosphonate.

A new multifunctional pyridine derivative was synthesized and studied as an efficient initiator for the polymerization of diethylvinylphosphonate (DEVP). The authors used a new pyridine compound (**473**) in the thiol-ene click reaction (a well-established coupling method) to link together poly-DEVP and thiocholesterol **95** (Scheme 110) [110].

Compound **474** exhibited good thermal response and low cytotoxicity against human embryonic renal cell lines (HEK-293) and immortalized human microvascular endothelial cells (HMEC-1). It was concluded that the introduction of the thiocholesterol anchor unit was advantageous regarding toxicity when compared to polymers without functionalization. The thiocholesterol conjugate **474** is interesting for many applications, since it is water-soluble, thermo-responsive, and biocompatible [110].

**Scheme 111:**
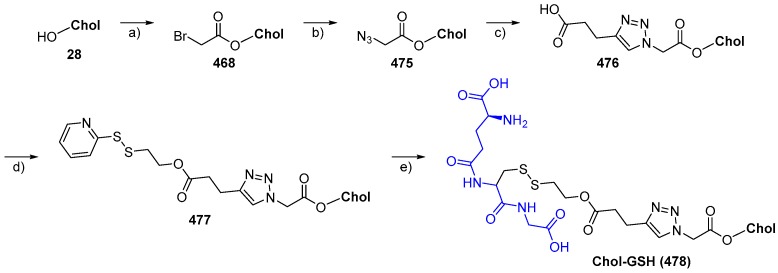
Synthesis of cholesterol-glutathione (**Ch-GSH**) bioconjugate. Reagents and conditions: **a**) 2-bromoacetyl bromide, Et_3_N, CH_2_Cl_2_, rt, overnight; **b**) NaN_3_, THF/H_2_O (4:1), 50 °C, overnight; **c**) 4-pentynoic acid, CuBr, PMDETA, THF/DMF (4:1), 35 °C, 48 h; **d**) 2-(pyridine-2-yldisulfanyl)ethanol, EDCl, DMAP, CH_2_Cl_2_, rt, 24 h; (**e**) glutathione, THF/H_2_O (3:1), rt, 6 h.

To take advantage of the important biological properties of cholesterol and glutathione for the cells, a cholesterol-glutathione (**Chol-GSH**) bioconjugate (**478**) was designed and used as a model amphiphilic biomolecule to make a co-assembly with lysozyme using a dialysis-assisted approach [111]. The synthetic route toward the **Chol-GSH** bioconjugate **478** involved a five-step reaction sequence, including esterification, 1,3-dipolar cycloaddition, and thiol‒disulfide exchange reactions (Scheme 111).

The authors applied a dialysis-assisted method of Ch-GSH and lysozyme to prepare bioactive self-assembled structures, which showed that hydrophobic cholesterol located in the walls, and hydrophilic GSH and lysozyme on the inner and outer surfaces. This result was explained based on the electrostatic interaction between GSH and lysozyme, which provided a driving force for the self-assembly, maintaining the bioactivity of lysozyme in the self-assembly process [111].

## 9. Conclusions

In this review, the role of cholesterol-based compounds in different research areas such as drug delivery, biological activities, liquid crystals, gelators, bioimaging, and purely synthetic applications was highlighted. In the drug delivery field, several examples of cholesterol derivatives were highlighted due to their applications in preclinical and clinical liposomal drug formulations to decrease membrane fluidity and provide favorable drug retention properties. Furthermore, in the last few years, some series of new cholesterol derivatives have also been developed for pharmacological applications as anticancer, antimicrobial, or antioxidant agents. In the bioimaging field, cholesterol has been used as a lipid anchor attached to fluorophores to study cellular membrane trafficking, imaging of cholesterol density, and liposome tracing, among many other bioimaging applications. The fact that cholesterol conjugates have much scientific interest in the field of materials science due to their liquid crystal phase behavior, as well as the ability to promote self-organization and hydrophobic interactions in aqueous media (gelation properties), was also demonstrated in this review. In this review, a general perspective was given of the main applications of cholesterol derivatives in several research fields, but also a concise perspective of the advances in their synthetic chemistry. Therefore, we described the synthetic pathway for different cholesterol derivatives alongside the corresponding application of the new compounds to furnish a general view from the synthetic and biological aspects of the most recently reported cholesterol-based compounds.

## Abbreviations List

Ac	acetyl
Ac_2_O	acetic anhydride
AcOH	acetic acid
AG	arabinogalactan
AIBN	2,2′-azobis(2-methylpropionitrile)
AIEE	aggregation induced enhanced emission
AL	β-alanine
AscONa	sodium ascorbate
ATRP	atom transfer radical polymerization
BBN	bombesin
Bn	benzyl
Boc_2_O	di-*tert*-butyl decarbonate
BODIPY	boron dipyrromethene
BtOH	*N*-hydroxybenzotriazole
Bz	benzoyl
CAE	cholesterol-arginine ester
β-CD	β-cyclodextrin
β-CD-NSP	β-cyclodextrin nanosponge
CDI	carbonyldiimidazole
CF	5,6-carboxyfluorescein
Chol	cholesterol
Chol-OA	oxyamine-terminated cholesterol
CHS	cholesterol hydrogen succinate
Ch-T	chloramine-T
CL	conventional liposomes
CuAAC	copper(I)-catalyzed azide-alkyne cycloaddition
CVS	crystal violet staining
Cyclen	1,4,7,10-tetraazacyclododecane
CYS	cystamine
DAIN	4-(diarylmethylene)imidazolinone
DBU	1,8-diazabicyclo[5.4.0]undec-7-ene
DCC	*N*,*N*′-dicyclohexylcarbodiimide
DCVJ	9-(2,2-dicyanovinyl)julolidine
DHPC	dihexanoylphosphatidylcholine
DIAD	diisopropyl azodicarboxylate
DIPEA	*N*,*N*-diisopropylethylamine
DMAP	dimethylaminopyridine
DMF	dimethylformamide
DMPC	dimyristoylphosphatidylcholine
DMSO	dimethyl sulfoxide
DMTAP	dimyristoyltrimethylammonium propane
DNA	deoxyribonucleic acid
DOPC	dioleoylphosphatidilcholine
DOPE	1,2-dioleoyl-*sn*-glycero-3-phosphoethanolamine
DOX	doxorubicin
DOX-NPs	doxorubicin loaded nanoparticles
DPPA	diphenylphosphoryl azide
DPPH	2,2-diphenyl-1-picrylhydrazyl radical
DTX	docetaxel
EDAC	1-ethyl-3-(3-dimethylaminopropyl)carbodiimide hydrochloride
EDCl	*N*-(3-dimethylaminopropyl)-*N*′-ethylcarbodiimide hydrochloride
Et_2_N	diethylamine
Et_3_N	triethylamine
Et_2_O	diethyl ether
EtOAc	ethyl acetate
EtOH	ethanol
GFP	green fluorescent protein
GSH	glutathione
HA	hyaluronic acid
*hb*PG	linear-hyperbranched amphiphilic polyglycerol
HOBt	*N*-hydroxybenzotriazole
HSA	human serum albumin
IC_50_	half-maximal inhibitory concentration
ICT	intramolecular charge transfer
l-AA	l-ascorbic acid
LC	liquid crystal
LMGs	low-molecular-weight gelators
MeCN	acetonitrile
MeOTf	methyl triflate
*m*-CPBA	*m*-chloroperoxybenzoic acid
min	minutes
MPP	mitochondria-penetrating peptide
MTT	3-(4,5-dimethylthiazo-2-yl)-2,5-diphenyltetrazolium bromide
MW	microwave irradiation
NaOAc	sodium acetate
NaOMe	sodium methoxide
NBD	nitrobenzoxadiazole
NBS	*N*-bromosuccinimide
NHS	*N*-hydroxysuccinimide
NOAC	nitrile oxide alkyne cycloaddition
NPs	nanoparticles
PBS	phosphate-buffered saline
PDC	pyridinium dichromate
PEG	polyethylene glycol
PET	positron emission tomography
*p*HPMA	poly[*N*-(2-hydroxypropyl)-methacrylamide]
PMDETA	*N*,*N*,*N*′,*N*″,*N*″-pentamethyldiethylenetriamine
PPA	phenylpropanolamine
PPh_3_	triphenylphosphine
*p*-TsCl	*p*-toluenesulfonyl chloride
PTX	paclitaxel
PyBOP	benzotriazole-1-yl-oxytripyrrolidinophosphonium hexafluorophosphate
RAFT	reversible addition fragmentation chain transfer
rt	room temperature
SA	succinic anhydride
SCID	severe combined immunodeficient
SML	surface-modified liposomes
SubPcs	subphthalocyanines
TACN	1,4,7-triazacyclononane
Tb	trilobolide
TBAB	tetrabutylammonium bromide
TBAF	tetrabutylammonium fluoride
TBAH	tetrabutylammonium hydroxide
TBAITBDMS	tetrabutylammonium iodide*tert*-butyldimethylsilyl
TBDMSCl	*tert**-*butyldimethylsilyl chloride
Tb-N_3_VA	trilobolide 8-*O*-azidovalerate
TBTA	tris[(1-benzyl-1*H*-1,2,3-triazol-4-yl)methyl]amine
*t*-BuOH	*tert*-butanol
*t*-BuOK	potassium *tert*-butoxide
*t*-BuOOH	*tert*-butyl hydroperoxide
TE	transfection efficiency
TEG	tetraethylene glycol
TFA	trifluoroacetic acid
TFAA	trifluoroacetic anhydride
THF	tetrahydrofuran
TIS	triisopropylsilane
TMSCl	trimethylsilyl chloride
TMSITMSOTf	iodotrimethylsilanetrimethylsilyl trifluoromethanesulfonate
TOAB	tetraoctylammonium bromide
TsCl	*p*-toluenesulfonyl chloride

## References

[1] Cerqueira N.M.F.S.A., Oliveira E.F., Gesto D.S., Santos-Martins D., Moreira C., Moorthy H.N., Ramos M.J., Fernandes P.A. (2016). Cholesterol Biosynthesis: A Mechanistic Overview. Biochemistry.

[2] Nes W.D. (2011). Biosynthesis of Cholesterol and Other Sterols. Chem. Rev..

[3] Habchi J., Chia S., Galvagnion C., Michaels T.C.T., Bellaiche M.M.J., Ruggeri F.S., Sanguanini M., Idini I., Kumita J.R., Sparr E. (2018). Cholesterol catalyses Aβ42 aggregation through a heterogeneous nucleation pathway in the presence of lipid membranes. Nat. Chem..

[4] Morzycki J.W. (2014). Recent advances in cholesterol chemistry. Steroids.

[5] Sercombe L., Veerati T., Moheimani F., Wu S.Y., Sood A.K., Hua S. (2015). Advances and Challenges of Liposome Assisted Drug Delivery. Front. Pharmacol..

[6] Vabbilisetty P., Sun X.-L. (2014). Liposome surface functionalization based on different anchoring lipids via Staudinger ligation. Org. Biomol. Chem..

[7] Saleem Q., Zhang Z., Petretic A., Gradinaru C.C., Macdonald P.M. (2015). Single Lipid Bilayer Deposition on Polymer Surfaces Using Bicelles. Biomacromolecules.

[8] Pathak P.O., Nagarsenker M.S., Barhate C.R., Padhye S.G., Dhawan V.V., Bhattacharyya D., Viswanathan C.L., Steiniger F., Fahr A. (2015). Cholesterol anchored arabinogalactan for asialoglycoprotein receptor targeting: Synthesis, characterization, and proof of concept of hepatospecific delivery. Carbohydr. Res..

[9] Crucianelli E., Bruni P., Frontini A., Massaccesi L., Pisani M., Smorlesi A., Mobbili G. (2014). Liposomes containing mannose-6-phosphate-cholesteryl conjugates for lysosome-specific delivery. RSC Adv..

[10] Silva A.T.M., Maia A.L.C., de Oliveira Silva J., de Barros A.L.B., Soares D.C.F., de Magalhaes M.T.Q., Jose Alves R., Ramaldes G.A. (2018). Synthesis of cholesterol-based neoglycoconjugates and their use in the preparation of liposomes for active liver targeting. Carbohydr. Res..

[11] Škorpilová L., Rimpelová S., Jurášek M., Buděšínský M., Lokajová J., Effenberg R., Slepička P., Ruml T., Kmoníčková E., Drašar P.B., Wimmer Z. (2017). BODIPY-based fluorescent liposomes with sesquiterpene lactone trilobolide. Beilstein J. Org. Chem..

[12] Lin Y.-K., Fang J.-Y., Wang S.-W., Lee R.-S. (2018). Synthesis and characterization of triple-responsive PNiPAAm-S-S-P(αN3CL-g-alkyne) copolymers bearing cholesterol and fluorescence monitor. React. Funct. Polym..

[13] Alberti D., Toppino A., Geninatti Crich S., Meraldi C., Prandi C., Protti N., Bortolussi S., Altieri S., Aime S., Deagostino A. (2014). Synthesis of a carborane-containing cholesterol derivative and evaluation as a potential dual agent for MRI/BNCT applications. Org. Biomol. Chem..

[14] Zhang X., Niebuur B.-J., Chytil P., Etrych T., Filippov S.K., Kikhney A., Wieland D.C.F., Svergun D.I., Papadakis C.M. (2018). Macromolecular *p*HPMA-Based Nanoparticles with Cholesterol for Solid Tumor Targeting: Behavior in HSA Protein Environment. Biomacromolecules.

[15] Singh P., Ren X., Guo T., Wu L., Shakya S., He Y., Wang C., Maharjan A., Singh V., Zhang J. (2018). Biofunctionalization of β-cyclodextrin nanosponges using cholesterol. Carbohydr. Pol..

[16] Li J., Ma Y.J., Wang Y., Chen B.Z., Guo X.D., Zhang C.Y. (2018). Dual redox/pH-responsive hybrid polymer-lipid composites: Synthesis, preparation, characterization and application in drug delivery with enhanced therapeutic efficacy. Chem. Eng. J..

[17] Tran T.-H., Nguyen C.T., Gonzalez-Fajardo L., Hargrove D., Song D., Deshmukh P., Mahajan L., Ndaya D., Lai L., Kasi R.M. (2014). Long Circulating Self-Assembled Nanoparticles from Cholesterol-Containing Brush-Like Block Copolymers for Improved Drug Delivery to Tumors. Biomacromolecules.

[18] Wang Z., Luo T., Sheng R., Li H., Sun J., Cao A. (2016). Amphiphilic Diblock Terpolymer PMAgala-*b*-P(MAA-*co*-MAChol)s with Attached Galactose and Cholesterol Grafts and Their Intracellular pH-Responsive Doxorubicin Delivery. Biomacromolecules.

[19] Jia L., Cui D., Bignon J., Di Cicco A., Wdzieczak-Bakala J., Liu J., Li M.-H. (2014). Reduction-Responsive Cholesterol-Based Block Copolymer Vesicles for Drug Delivery. Biomacromolecules.

[20] Dolor A., Kierstead P., Dai Z., Szoka F.C. (2018). Sterol-modified PEG lipids: Alteration of the bilayer anchoring moiety has an unexpected effect on liposome circulation. Chem. Commun..

[21] Zhu Z., Li D., Li Y., Yang X., Pan W. (2017). In vitro–*in vivo* evaluation of hyaluronic acid-based amphiphilic copolymers for tumour targeted delivery: The role of hydrophobic groups. RSC Adv..

[22] Mallick S., Thuy L.T., Lee S., Park J.I., Choi J.S. (2018). Liposomes containing cholesterol and mitochondria-penetrating peptide (MPP) for targeted delivery of antimycin A to A549 cells. Colloids Surf. B.

[23] Ju J., Huan M.-L., Wan N., Hou Y.-L., Ma X.-X., Jia Y.-Y., Li C., Zhou S.-Y., Zhang B.-L. (2016). Cholesterol derived cationic lipids as potential non-viral gene delivery vectors and their serum compatibility. Bioorg. Med. Chem. Lett..

[24] Vulugundam G., Kumar K., Kondaiah P., Bhattacharya S. (2015). Efficacious redox-responsive gene delivery in serum by ferrocenylated monomeric and dimeric cationic cholesterols. Org. Biomol. Chem..

[25] Wang H.-J., He X., Zhang Y., Zhang J., Liu Y.-H., Yu X.-Q. (2015). Hydroxyl-containing non-viral lipidic gene vectors with macrocyclic polyamine headgroups. RSC Adv..

[26] Puchkov P.A., Perevoshchikova K.A., Kartashova I.A., Luneva A.S., Kabilova T.O., Morozova N.G., Zenkova M.A., Maslov M.A. (2017). Polycationic amphiphiles based on triethylenetetramine and their transfection efficacy. Russ. J. Bioorg. Chem..

[27] Monpara J., Kanthou C., Tozer G.M., Vavia P.R. (2018). Rational Design of Cholesterol Derivative for Improved Stability of Paclitaxel Cationic Liposomes. Pharm. Res..

[28] van Elk M., Deckers R., Oerlemans C., Shi Y., Storm G., Vermonden T., Hennink W.E. (2014). Triggered Release of Doxorubicin from Temperature-Sensitive Poly(*N*-(2-hydroxypropyl)-methacrylamide mono/dilactate) Grafted Liposomes. Biomacromolecules.

[29] Asayama S., Nagashima K., Negishi Y., Kawakami H. (2018). Byproduct-Free Intact Modification of Insulin by Cholesterol End-Modified Poly(ethylene glycol) for in Vivo Protein Delivery. Bioconj. Chem..

[30] Rega M., Jiménez C., Rodríguez J. (2007). 6*E*-Hydroximinosteroid homodimerization by cross-metathesis processes. Steroids.

[31] Richmond V., Careaga V.P., Sacca P., Calvo J.C., Maier M.S. (2014). Synthesis and cytotoxic evaluation of four new 6*E*-hydroximinosteroids. Steroids.

[32] Soto-Castro D., Lara Contreras R.C., Pina-Canseco M.D.S., Santillan R., Hernandez-Huerta M.T., Negron Silva G.E., Perez-Campos E., Rincon S. (2017). Solvent-free synthesis of 6β-phenylamino-cholestan-3β,5α-diol and (25*R*)-6β-phenylaminospirostan-3β,5α-diol as potential antiproliferative agents. Steroids.

[33] Bu M., Cao T., Li H., Guo M., Yang B.B., Zhou Y., Zhang N., Zeng C., Hu L. (2017). Synthesis and biological evaluation of novel steroidal 5α,8α-endoperoxide derivatives with aliphatic side-chain as potential anticancer agents. Steroids.

[34] Huang Y., Yang C., Zhan J., Gan C., Liu Z., Pang C., Chen H., Cui J. (2017). Synthesis and antiproliferative activity of novel A-homo-B-norsteroid thiadiazole derivatives. Tetrahedron Lett..

[35] Martínez-Pascual R., Meza-Reyes S., Vega-Baez J.L., Merino-Montiel P., Padrón J.M., Mendoza Á., Montiel-Smith S. (2017). Novel synthesis of steroidal oximes and lactams and their biological evaluation as antiproliferative agents. Steroids.

[36] D’Yakonov V.A., Tuktarova R.A., Dzhemileva L.U., Ishmukhametova S.R., Yunusbaeva M.M., Dzhemilev U.M. (2018). Catalytic cyclometallation in steroid chemistry V: Synthesis of hybrid molecules based on steroid oximes and (5*Z*,9*Z*)-tetradeca-5,9-dienedioic acid as potential anticancer agents. Steroids.

[37] Huang Y., Wen H., Zheng J., Gan C., Pang L., Pang C., Liu X., Zhan J., Cui J. (2018). Synthesis, characterization, and biological evaluations of some steryl 2-methoxybenzoates as anticancer agents. Nat. Prod. Res..

[38] Peters A.D., McCallion C., Booth A., Adams J.A., Rees-Unwin K., Pluen A., Burthem J., Webb S.J. (2018). Synthesis and biological activity of a CXCR4-targeting bis(cyclam) lipid. Org. Biomol. Chem..

[39] Aly M.R.E.S., Saad H.A., Mohamed M.A.M. (2015). Click reaction based synthesis, antimicrobial, and cytotoxic activities of new 1,2,3-triazoles. Bioorg. Med. Chem. Lett..

[40] Aly M.R.E.S., Saad H.A., Abdel-Hafez S.H. (2015). Synthesis, antimicrobial and cytotoxicity evaluation of new cholesterol congeners. Beilstein J. Org. Chem..

[41] Shamsuzzaman, Khanam H., Dar A.M., Siddiqui N., Rehman S. (2016). Synthesis, characterization, antimicrobial and anticancer studies of new steroidal pyrazolines. J. Saudi Chem. Soc..

[42] Shamsuzzaman, Mashrai A., Khanam H., Asif M., Ali A., Sherwani A., Owais M. (2015). Green synthesis and biological evaluation of steroidal 2*H*-pyrans as anticancer and antioxidant agents. J King Saud Univ. Sci..

[43] Ali A., Asif M., Alam P., Jane Alam M., Asif Sherwani M., Hasan Khan R., Ahmad S., Shamsuzzaman (2017). DFT/B3LYP calculations, in vitro cytotoxicity and antioxidant activities of steroidal pyrimidines and their interaction with HSA using molecular docking and multispectroscopic techniques. Bioorg. Chem..

[44] Saikia P., Kaishap P.P., Goswami J., Singh A.K., Deka Boruah H.P., Gogoi S., Boruah R.C. (2014). Synthesis of steroidal and nonsteroidal vicinal heterocyclic alcohols, *N*-(1-cycloalkenyl)heterocycles and their antibacterial studies. Steroids.

[45] Morake M., Coertzen D., Ngwane A., Wentzel J.F., Wong H.N., Smit F.J., Birkholtz L.M., Pietersen R.D., Baker B., Wiid I. (2018). Preliminary Evaluation of Artemisinin-Cholesterol Conjugates as Potential Drugs for the Treatment of Intractable Forms of Malaria and Tuberculosis. ChemMedChem.

[46] Aly M.R.E.S., El Azab I.H., Gobouri A.A. (2018). Synthesis, antimicrobial and photoelectric potency of new ferrocene-based congeners. Mon. Chem..

[47] Ansari A., Ali A., Asif M., Rauf M.A., Owais M., Shamsuzzaman (2018). Facile one-pot multicomponent synthesis and molecular docking studies of steroidal oxazole/thiazole derivatives with effective antimicrobial, antibiofilm and hemolytic properties. Steroids.

[48] Begum A., Borah P., Chowdhury P. (2016). Microwave (MW) promoted high yield expedient synthesis of steryl ferulates—A class of novel biologically active compounds: A comparative study of their antioxidant activity with that of naturally occurring γ-oryzanol. Steroids.

[49] Wöhrle T., Wurzbach I., Kirres J., Kostidou A., Kapernaum N., Litterscheidt J., Haenle J.C., Staffeld P., Baro A., Giesselmann F., Laschat S. (2016). Discotic Liquid Crystals. Chem. Rev..

[50] Hiremath U.S. (2014). Synthesis and characterization of novel chiral dimers exhibiting highly frustrated liquid crystal phases. Tetrahedron.

[51] Yeap G.-Y., Alshargabi A., Mahmood W.A.K., Han C.-C., Lin H.-C., Santo M., Ito M.M. (2015). Synthesis, characterization and molecular organization for induced smectic phase of triazole ring in non-symmetric liquid crystalline dimer. Tetrahedron.

[52] Guo H., Yang F., Liu W., Lai J. (2015). Novel supramolecular liquid crystals: Synthesis and mesomorphic properties of calix[4]arene-cholesterol derivatives. Tetrahedron Lett..

[53] Zhang X., Guo H., Yang F., Yuan J. (2016). Ion complexation-controlled columnar mesophase of calix[4]arene–cholesterol derivatives with Schiff-base bridges. Tetrahedron Lett..

[54] Xiong J., Lin X., Guo H., Yang F., Lai J. (2018). Liquid crystalline oligomers derived from cholesterol: Synthesis and columnar mesomorphism. Liq. Cryst..

[55] Gupta M., Pal V., Pal S.K. (2018). Photo-responsive liquid crystals derived from azobenzene centered cholesterol-based tetramers. New J. Chem..

[56] Gupta S.K., Setia S., Sidiq S., Gupta M., Kumar S., Pal S.K. (2013). New perylene-based non-conventional discotic liquid crystals. RSC Adv..

[57] Zhu M., Guo H., Yang F., Wang Z. (2017). Synthesis, mesomorphic and photophysical properties of novel triads and pentads of perylene liquid crystals with cholesterol units at the bay-position. RSC Adv..

[58] Chen S., Hong B., Guo H., Yang F. (2018). The mesomorphic and photophysical properties of perylene liquid crystals with different bay-rigid spacers. Liq. Cryst..

[59] Champagne P.-L., Ester D., Aldosari S., Williams V.E., Ling C.-C. (2018). Synthesis and comparison of mesomorphic behaviour of a cholesterol-based liquid crystal dimer and analogous monomers. Liq. Cryst..

[60] Liu X., Guo Z., Xie Y., Chen Z., Hu J., Yang L. (2018). Synthesis and liquid crystal behavior of new side chain aliphatic polycarbonates based on cholesterol. J. Mol. Liq..

[61] Xiong Y., Zheng S., Zhu L., Guo H., Yang F. (2018). Novel liquid crystals with high fluorescence: Synthesis, mesomorphic and photophysical properties of cholesterol-triazine-BODIPY trimers. J. Mol. Struct..

[62] Ooi Y.-H., Yeap G.-Y. (2018). λ-Shaped liquid crystal trimers with dual terminal cholesteryl moieties: Synthesis and concomitant of N*, SmA and cholesteric glassy phases. Liq. Cryst..

[63] Frizon T.E., Jamal R., Sumbal S., Bechtold I.H., Gallardo H., Braga A.L. (2015). Synthesis of Functionalized Organoselenium Materials: Selenides and Diselenides Containing Cholesterol. Eur. J. Org. Chem..

[64] Beaulieu R., Gottis S., Meyer C., Grand E., Deveaux V., Kovensky J., Stasik I. (2015). Cholesteryl and diosgenyl glycosteroids: Synthesis and characterization of new smectic liquid crystals. Carbohydr. Res..

[65] Skilling K.J., Citossi F., Bradshaw T.D., Ashford M., Kellam B., Marlow M. (2014). Insights into low molecular mass organic gelators: A focus on drug delivery and tissue engineering applications. Soft Matter.

[66] Draper E.R., Adams D.J. (2017). Low-Molecular-Weight Gels: The State of the Art. Chem.

[67] Devi M., Dhir A., Dhir A., Pooja, Pradeep C.P. (2014). New triangular steroid-based A(LS)3 type gelators for selective fluoride sensing application. RSC Adv..

[68] Geng L., Feng G., Wang S., Yu X., Xu Z., Zhen X., Wang T. (2015). Fluoride-responsive organogel containing azobenzyl and cholesterol units. J. Fluorine Chem..

[69] Ghosh K., Panja S. (2015). Coumarin-based supramolecular gelator: A case of selective detection of F^−^ and HP_2_O_7_^3−^. RSC Adv..

[70] Pang X., Yu X., Xie D., Li Y., Geng L., Ren J., Zhen X. (2016). Tunable multicolor emissions in a monocomponent gel system by varying the solvent, temperature and fluoride anion. Org. Biomol. Chem..

[71] Panja S., Ghosh S., Ghosh K. (2018). Pyridine/pyridinium symmetrical bisamides as functional materials: Aggregation, selective sensing and drug release. New J. Chem..

[72] Huibin S., Shujuan L., Qiang Z., Wei H. (2015). Multiple-Stimuli Responsive Luminescent Gels Based on Cholesterol Containing Benzothiadiazole Fluorophores. Chin. J. Chem..

[73] Panja A., Ghosh K. (2018). Selective sensing of Hg^2+^ via sol–gel transformation of a cholesterol-based compound. Supramol. Chem..

[74] Panja A., Ghosh K. (2018). Diaminomalenonitrile-decorated cholesterol-based supramolecular gelator: Aggregation, multiple analyte (hydrazine, Hg^2+^ and Cu^2+^) detection and dye adsorption. New J. Chem..

[75] Panja A., Ghosh K. (2018). Cholesterol-based diazine derivative: Selective sensing of Ag^+^ and Fe^3+^ ions through gelation and the performance of metallogels in dye and picric acid adsorption from water. Mater. Chem. Front..

[76] Ren Y., Wang B., Zhang X. (2015). Synthesis of photoresponsive cholesterol-based azobenzene organogels: Dependence on different spacer lengths. Beilstein J. Org. Chem..

[77] Tan X., Li Z., Xia M., Cheng X. (2016). Reversible photoresponsive chiral liquid crystal and multistimuli responsive organogels based on a cholesterol-azobenzene dimesogen. RSC Adv..

[78] Yu X., Chen H., Shi X., Albouy P.-A., Guo J., Hu J., Li M.-H. (2018). Liquid crystal gelators with photo-responsive and AIE properties. Mater. Chem. Front..

[79] Shimasaki T., Okamiya Y., Sato R., Hara K., Nakamura T., Teramoto N., Shibata M. (2016). Synthesis, structure and properties of cholesterol-based A(LS)2- and A(LS)3-type gelators without hydrogen bond linkers. Tetrahedron.

[80] Yao C., Sun Q., Xia W., Zhang J., Lin C., Wang L. (2017). Ferrocenyl-guest tunable organogel constructed from a Pillar[6]arene-functionalized cholesterol derivative. J. Organomet. Chem..

[81] Li G., Wu J., Wang B., Yan S., Zhang K., Ding J., Yin J. (2015). Self-Healing Supramolecular Self-Assembled Hydrogels Based on Poly(l-glutamic acid). Biomacromolecules.

[82] Guo Z., Park S., Yoon J., Shin I. (2014). Recent progress in the development of near-infrared fluorescent probes for bioimaging applications. Chem. Soc. Rev..

[83] Chauhan D.P., Saha T., Lahiri M., Talukdar P. (2014). BODIPY based ‘click on’ fluorogenic dyes: Application in live cell imaging. Tetrahedron Lett..

[84] Byrd K.M., Arieno M.D., Kennelly M.E., Estiu G., Wiest O., Helquist P. (2015). Design and synthesis of a crosslinker for studying intracellular steroid trafficking pathways. Bioorg. Med. Chem..

[85] Kim B.-K., Seu Y.-B., Choi J.-S., Park J.-W., Doh K.-O. (2015). Synthesis and validation of novel cholesterol-based fluorescent lipids designed to observe the cellular trafficking of cationic liposomes. Bioorg. Med. Chem. Lett..

[86] Reibel A.T., Müller S.S., Pektor S., Bausbacher N., Miederer M., Frey H., Rösch F. (2015). Fate of Linear and Branched Polyether-Lipids In Vivo in Comparison to Their Liposomal Formulations by ^18^F-Radiolabeling and Positron Emission Tomography. Biomacromolecules.

[87] Palakollu V., Kanvah S. (2015). Cholesterol-tethered AIEE fluorogens: Formation of self-assembled nanostructures. RSC Adv..

[88] Wercholuk A.N., Thuman J.M., Stanley J.L., Sargent A.L., Anderson E.S., Allen W.E. (2016). Incorporation of fluorophore-cholesterol conjugates into liposomal and mycobacterial membranes. Bioorg. Med. Chem..

[89] Bernhard Y., Gigot E., Goncalves V., Moreau M., Sok N., Richard P., Decréau R.A. (2016). Direct subphthalocyanine conjugation to bombesin vs. indirect conjugation to its lipidic nanocarrier. Org. Biomol. Chem..

[90] Ikejiri M., Mori K., Miyagi R., Konishi R., Chihara Y., Miyashita K. (2017). A hybrid molecule of a GFP chromophore analogue and cholestene as a viscosity-dependent and cholesterol-responsive fluorescent sensor. Org. Biomol. Chem..

[91] Tomkiel A.M., Kowalski J., Płoszyńska J., Siergiejczyk L., Łotowski Z., Sobkowiak A., Morzycki J.W. (2014). Electrochemical synthesis of glycoconjugates from activated sterol derivatives. Steroids.

[92] Tomkiel A.M., Biedrzycki A., Ploszynska J., Narog D., Sobkowiak A., Morzycki J.W. (2015). 3α,5α-Cyclocholestan-6β-yl ethers as donors of the cholesterol moiety for the electrochemical synthesis of cholesterol glycoconjugates. Beilstein J. Org. Chem..

[93] Davis R.A., Fettinger J.C., Gervay-Hague J. (2014). Tandem Glycosyl Iodide Glycosylation and Regioselective Enzymatic Acylation Affords 6-*O*-Tetradecanoyl-α-d-cholesterylglycosides. J. Org. Chem..

[94] Mao R.-Z., Guo F., Xiong D.-C., Li Q., Duan J., Ye X.-S. (2015). Photoinduced C-S Bond Cleavage of Thioglycosides and Glycosylation. Org. Lett..

[95] Davis R.A., Fettinger J.C., Gervay-Hague J. (2015). Synthesis of cholesteryl-α-d-lactoside via generation and trapping of a stable β-lactosyl iodide. Tetrahedron Lett..

[96] Weiss S., Neu P.M., Ludwig C., Schober S., Mittelbach M. (2018). Novel Method for the Synthesis of Cholesteryl Glucosides starting from Disaccharides. Eur. J. Lipid Sci. Technol..

[97] Algay V., O’Sullivan J., Heaney F. (2014). C-3β-Tethered Functional Cholesterol Conjugates by Nitrile Oxide Alkyne Cycloaddition (NOAC). Eur. J. Org. Chem..

[98] Alarcon-Manjarrez C., Arcos-Ramos R., Alamo M.F., Iglesias-Arteaga M.A. (2016). Synthesis, NMR and crystal characterization of dimeric terephthalates derived from epimeric 4,5-seco-cholest-3-yn-5-ols. Steroids.

[99] Shibuya K., Watanabe T., Urano Y., Takabe W., Noguchi N., Kitagishi H. (2016). Synthesis of 24(*S*)-hydroxycholesterol esters responsible for the induction of neuronal cell death. Bioorg. Med. Chem..

[100] Sarkar A., Das J., Ghosh P. (2017). *p*-TsOH-Catalyzed one-pot transformation of di- and trihydroxy steroids towards diverse A/B-ring oxo-functionalization. New J. Chem..

[101] Borthakur M., Barthakur M.G., Boruah R.C. (2008). Microwave promoted one-pot synthesis of novel A-ring fused steroidal dehydropiperazines. Steroids.

[102] Ansari A., Ali A., Asif M. (2018). Microwave-assisted MgO NP catalyzed one-pot multicomponent synthesis of polysubstituted steroidal pyridines. New J. Chem..

[103] Barthakur M.G., Gogoi S., Dutta M., Boruah R.C. (2009). A facile three-component solid phase synthesis of steroidal A-ring fused pyrimidines under microwave irradiation. Steroids.

[104] Dutta M., Saikia P., Gogoi S., Boruah R.C. (2013). Microwave-promoted and Lewis acid catalysed synthesis of steroidal a- and d-ring fused 4,6-diarylpyridines. Steroids.

[105] Schulze M., Scott D.E., Scherer A., Hampel F., Hamilton R.J., Gray M.R., Tykwinski R.R., Stryker J.M. (2015). Steroid-Derived Naphthoquinoline Asphaltene Model Compounds: Hydriodic Acid Is the Active Catalyst in I_2_-Promoted Multicomponent Cyclocondensation Reactions. Org. Lett..

[106] Berg M., Nozinovic S., Engeser M., Lützen A. (2015). A Cholesterol Containing pH-Sensitive Bistable [2]Rotaxane. Eur. J. Org. Chem..

[107] Venkataraman S., Mineart K.P., Prabhu V.M., Hedrick J.L., Yang Y.Y. (2018). Cholesterol functionalized aliphatic *N*-substituted 8-membered cyclic carbonate. Polym. Chem..

[108] Liu H., Wang R., Wei J., Cheng C., Zheng Y., Pan Y., He X., Ding M., Tan H., Fu Q. (2018). Conformation-Directed Micelle-to-Vesicle Transition of Cholesterol-Decorated Polypeptide Triggered by Oxidation. J. Am. Chem. Soc..

[109] Kozanecka W., Mrowczynska L., Pospieszny T., Jasiewicz B., Gierszewski M. (2015). Synthesis, spectroscopy, theoretical and biological studies of new gramine-steroids salts and conjugates. Steroids.

[110] Schwarzenböck C., Schaffer A., Pahl P., Nelson P.J., Huss R., Rieger B. (2018). Precise synthesis of thermoresponsive polyvinylphosphonate-biomolecule conjugates via thiol–ene click chemistry. Polym. Chem..

[111] Tao Y., Ma X., Cai Y., Liu L., Zhao H. (2018). Coassembly of Lysozyme and Amphiphilic Biomolecules Driven by Unimer–Aggregate Equilibrium. J. Phys. Chem. B.

